# Thermo-elastodynamics of nonlinearly viscous solids

**DOI:** 10.1007/s00526-026-03282-9

**Published:** 2026-02-28

**Authors:** Stefano Almi, Rufat Badal, Manuel Friedrich, Sebastian Schwarzacher

**Affiliations:** 1 Department of Mathematics and Applications “R. Caccioppoli”, University of Naples Federico II, Via Cintia, Monte S. Angelo, 80126 Napoli, Italy; 2 Department of Mathematics, Friedrich-Alexander Universität Erlangen-Nürnberg, Cauerstr. 11, D-91058 Erlangen, Germany; 3 Department of Mathematics, Johannes Kepler Universität Linz, Altenberger-strasse 69, 4040 Linz, Austria; 4 Department of Mathematics, Uppsala University, Ångströmlaboratoriet, Lägerhyddsvägen 1, 752 37 Uppsala, Sweden; 5 Department of Mathematical Analysis, Charles University, Sokolovská 83, 186 75 Praha 8, Czech Republic

**Keywords:** 74D10, 74F05, 74H20, 35A15, 35Q74, 35Q79

## Abstract

In this paper, we study the thermo-elastodynamics of nonlinearly viscous solids in the Kelvin-Voigt rheology where both the elastic and the viscous stress tensors comply with the frame-indifference principle. The system features a force balance including inertia in the frame of nonsimple materials and a heat-transfer equation which is governed by the Fourier law in the deformed configuration. Combining a staggered minimizing movement scheme for quasi-static thermoviscoelasticity [2, 37] with a variational approach to hyperbolic PDEs developed in [5], our main result consists in establishing the existence of weak solutions in the dynamic case. This is first achieved by including an additional higher-order regularization for the dissipation. Afterwards, this regularization can be removed by passing to a weaker formulation of the heat-transfer equation which complies with a total energy balance. The latter description hinges on regularity theory for the fourth order *p*-Laplacian which induces regularity estimates of the deformation beyond the standard estimates available from energy bounds. Besides being crucial for the proof, these extra regularity properties might be of independent interest and seem to be new in the setting of nonlinear viscoelasticity, also in the static or quasi-static case.

## Introduction

Understanding the coupling between mechanical and thermal phenomena in viscoelastic solids has been a mainstay in the mathematical and physical literature over the last decades. Even at small strains, the problem is notoriously difficult since the heat-transfer equation has no obvious variational structure due to the low regularity of data. In fact, after the pioneering work of Dafermos [16–19] in one space dimension, new fundamental ideas related to the existence theory for parabolic equations with measure-valued data developed in [9, 10] were needed to obtain results in three dimensions [8, 11, 12, 47]. At large strains, the problem is still considered to be extremely difficult even in the isothermal case, due to the highly nonlinear nature of models respecting material frame indifference [1]. For some results without temperature coupling, we refer to [39, 40] for existence of global-in-time weak solutions for initial data sufficiently close to a smooth equilibrium and to a local-in-time existence result [35]. By now, more general settings can only be treated by passing to weaker solution concepts such as measure-valued solutions [20, 21, 31]. Resorting to energy densities with higher-order spatial gradients, i.e., to so-called nonsimple materials [49, 50], existence of weak solutions has been shown in [26, 37] for the quasi-static case (without inertia) and in [5] for the dynamic case (with inertia). The variational approach adopted in these papers is quite flexible and has led to various extensions in the last years, ranging from models for dimension reduction [27–29], to problems with self-contact [13, 14, 34], approximability [15], diffusion [51], or homogenization [30], to applications for fluid-structure interactions [5–7, 33].

Nonlinear frame-indifferent models in thermoviscoelasticity were analyzed only very recently [2–4, 37], again adopting the concept of nonsimple materials, yet neglecting inertial effects. The goal of this work is to extend this analysis to the setting of thermo-elastodynamics including inertia. While our work follows the Lagrangian perspective, let us mention that in the last years several works appeared in the isothermal and nonisothermal framework which employ the alternative Eulerian approach instead, see [43–46, 48]. In this context, higher-order gradients are involved rather in the dissipative than in the conservative part, which sometimes is referred to as multipolar viscous solids. Besides adopting the Lagrangian framework, a main motivation of our work is to establish an existence result *without* higher-order regularization of the dissipation.

We now introduce the large-strain model in more detail. In the Kelvin-Voigt rheology, the force balance of a nonlinearly viscoelastic material in a setting of nonsimple materials is given by the system

1.1 $$\begin{aligned} f = \rho \partial ^{2}_{tt} y - \textrm{div} \big ( \partial _{F} W ( \nabla y, \theta ) + \partial _{\dot{F}} R(\nabla y, \partial _{t} \nabla y, \theta ) - \nabla ({D H}(\Delta y)) \big ) \qquad \text {in }[0,T]\times \Omega . \end{aligned}$$

Here, [0, *T*] is a process time interval with $$T > 0$$, $$\Omega \subset \mathbb {R}^d$$ ($$d=2,3$$) denotes the reference configuration, $$y :[0, T] \times \Omega \rightarrow \mathbb {R}^d$$ is the time-dependent deformation, $$\theta :[0, T] \times \Omega \rightarrow [0,\infty )$$ denotes the temperature, and $$f :[0, T] \times \Omega \rightarrow \mathbb {R}^d$$ is a volume density of external forces acting on $$\Omega $$. The free energy density $$W :\mathbb {R}^{d\times d} \times [0, \infty ) \rightarrow \mathbb {R}\cup \{+\infty \}$$ depends on the deformation gradient $$\nabla y$$ (with placeholder $$F \in \mathbb {R}^{d\times d}$$) and respects frame indifference under rotations as well as positivity of the determinant of $$\nabla y$$. Additionally, adopting the framework of nonsimple materials, the stored energy features a contribution depending on the Laplacian $$\Delta y$$ given in terms of a convex potential $$H:\mathbb {R}^d \rightarrow \mathbb {R}$$ with *p*-growth for some $$ p>d$$. Finally, $$R :\mathbb {R}^{d \times d} \times \mathbb {R}^{d \times d} \times [0, \infty ) \rightarrow \mathbb {R}$$ denotes a (pseudo)potential of dissipative forces ($$\dot{F}$$ is the time derivative of *F*). As observed by Antman [1], *R* must comply with a time-continuous frame indifference principle meaning that *R* can be written in terms of the right Cauchy-Green tensor $$C :=F^T F$$ and its time derivative $$\dot{C} :=\dot{F}^T F + F^T \dot{F}$$, see (D.1) below for details.

The system (1.1) is coupled to a heat-transfer equation of the form

1.2 $$\begin{aligned}  &   c_V(\nabla y,\theta ) \, \partial _t \theta = {{\,\textrm{div}\,}}(\mathcal {K}(\nabla y, \theta ) \nabla \theta ) + \partial _{\dot{F}} R(\nabla y, \nabla \partial _t y, \theta ) : \nabla \partial _t y + \theta {\partial _{F \theta }^2} W (\nabla y, \theta ) : \nabla \partial _t y \nonumber \\  &   \quad \text {in }[0,T]\times \Omega , \end{aligned}$$

where $c_V(F,\theta ) = -\theta \partial ^2_{\theta \theta } W(F, \theta )$ is the heat capacity, $\mathcal {K}$ denotes the matrix of the heat-conductivity coefficients, and the last term plays the role of an adiabatic heat source. This corresponds to a heat transfer modeled by the Fourier law in the deformed configuration which is pulled back to the reference configurations and thus includes dependence on the deformation gradient. The coupled system (1.1)–(1.2) is complemented with suitable initial and boundary conditions, see (2.18)–(2.19) below.

The goal of this article is to establish an existence result for weak solutions to the nonlinear thermo-elastodynamic system (1.1)–(1.2), see Theorem 2.5. Our proof strategy heavily hinges on two recent advances in the variational analysis of nonlinearly elastic solids: we combine the staggered minimizing movement scheme for proving existence results in quasi-static thermoviscoelasticity [2, 37] with a variational approach to hyperbolic PDEs [5] which allows to include inertia.

In the following, we describe the main ingredients for the proof in more detail. The fundamental idea in [5] consists in replacing the acceleration term $\rho \partial _{tt}^2 y$ by a discrete difference $\rho \frac{\partial _t y - \partial _t y(\cdot - h)}{h}$ which allows to turn the hyperbolic problem (1.1) into a parabolic one. The latter time-delayed problem can be approximated by a time-discretized scheme as in [2, 37] with time step $\tau >0$. Then, given solutions to the discretized problems with two different length scales $\tau$ and *h* (called the velocity and the acceleration time scale, respectively), one first passes to $\tau \rightarrow 0$ and afterwards to $h \rightarrow 0$ to obtain a weak solution for (1.1). As in [37], a generalized version of Korn’s inequality [38] relying on the second-order regularization is essential in order to tame the nonlinearity arising from the frame indifference of the dissipation term. Concerning the coupling to the heat-transfer equation, the approach in [2, 37] crucially relies on the theory of parabolic equations with measure-valued right-hand side [10]. A delicate part of the proof lies in the passage to the limit $\tau \rightarrow 0$ in the dissipation term $\partial _{\dot{F}} R(\nabla y, \nabla \partial _t y, \theta ): \nabla \partial _t y$, see (1.2). For this, strong convergence of the time-discrete approximations $\nabla \partial _t y_\tau$ is indispensable which is guaranteed by exploiting the convergence of a mechanical energy balance, cf. [37, Proposition 5.1] for details.

Although all techniques mentioned above are crucial ingredients in our work, it turns out that they do not suffice in the setting with heat coupling *and* inertia. The main reason lies in missing regularity which impedes the derivation of a mechanical energy balance. To explain this issue, let as consider the simplified problem

1.3 $$\begin{aligned} \rho \partial _{tt}^2 y -\Delta \partial _t y +\Delta (|\Delta y|^{p-2} \Delta y) =f, \end{aligned}$$

which arises from (1.1) by neglecting the first Piola-Kirchhoff stress tensor $\partial _{F} W$, and considering a linear variant of $\partial _{\dot{F}} R$ as well as a *p*-homogeneous variant of *H*. In the quasi-static case $\rho = 0$ or in the time-delayed problem where $\rho \partial _{tt}^2 y$ is replaced by the discrete difference $\rho \frac{\partial _t y - \partial _t y(\cdot - h)}{h}$, a test of the time-discretized problem with $\partial _t y$ and an integration by parts (neglecting boundary terms) leads to the natural energy bounds $\Delta y\in L^\infty ([0,T]; L^p(\Omega ))$ and $\nabla \partial _t y \in L^2([0,T];L^2(\Omega ))$. Then, in the case $\rho = 0$, a mechanical energy balance is achieved by testing (1.3) with $\partial _t y$, cf. [2, Equation (4.11)]. In this context, the term $\Delta (|\Delta y|^{p-2} \Delta y)$ might in principle not have the correct duality coupling to apply the chain rule. However, since the other two terms *f* and $\Delta \partial _t y$ are in duality, also the delicate fourth-order term can be handled by comparison. In contrast, for $\rho >0$, the two terms $\rho \partial _{tt}^2 y$ and $\Delta (|\Delta y|^{p-2} \Delta y)$ are not in duality and the chain rule (and thus the mechanical energy balance) may fail.

This fundamental issue has already been observed in [37, Remark 6.6]. A possible workaround lies in adding an additional regularization for the dissipation, see (2.17a), which in the simplified setting reads as

1.4 $$\begin{aligned} \rho \partial _{tt}^2 y -\Delta \partial _t y +\Delta (|\Delta y|^{p-2} \Delta y) - \varepsilon \partial _t \Delta ^3 y =f. \end{aligned}$$

With the test $\partial _t y$, this induces the energy bound $\nabla \Delta \partial _t y \in L^2([0,T]; L^2(\Omega ))$ which is strong enough to recover the chain rule. In this case, a mechanical energy balance can be guaranteed and we can follow the strategy devised in [2, 37] and [5], see Theorem 2.2. (Note that we choose a simple higher-order regularization which does not comply with the principle of dynamical frame indifference. A frame-indifferent regularization would necessarily be very nonlinear.) This regularized setting is related to [46] where existence results under higher-order regularizations of the dissipation have been derived in a Eulerian settting. Yet, a main motivation of our work is to derive an existence result *without* such regularization.

Our strategy relies on passing to a weaker formulation of the heat-transfer equation (1.2) which is inspired by the derivation of a total energy balance (see [37, Equation (2.21)] or (2.28) below) and does not feature the delicate dissipation term $\partial _{\dot{F}} R(\nabla y, \nabla \partial _t y, \theta ): \nabla \partial _t y$, see (2.27) for details. On a formal level, the idea is to test (1.1) with $\partial _t y$ which allows to replace the dissipation term in (1.2). As discussed above, however, this test is actually not allowed in (1.3). Therefore, we perform this replacement first on the regularized level (1.4), and afterwards we pass to the limit $\varepsilon \rightarrow 0$. This procedure leads to a modified weak formulation of the system which does not guarantee a mechanical energy balance but has the essential feature that the total energy is in equilibrium with the work by external body forces and heat sources, see (2.28). Moreover, the solution concept introduced here becomes a standard weak solution or a strong solution once the necessary regularity properties for the deformation and the temperature are available.

Although curing the issue with the dissipation, the passage to the weaker modified setting causes a new problem: the resulting weak formulation features a third-order term $\nabla (D{H}(\Delta y ))$ which is not compatible with the available energy bound $\Delta y \in L^\infty ([0,T];L^p(\Omega ))$, see below (1.3). Therefore, it is necessary to improve the regularity of the deformation. Loosely speaking, this is achieved by testing (1.3) with $-\Delta y$ which after integration by parts (omitting any boundary terms) leads to an *elliptic estimate*. In fact, using $\nabla \partial _t y \in L^2([0,T];L^2(\Omega ))$, the first term $|\int \partial _{tt}^2 y \Delta y \textrm{d}t \textrm{d}x| \le C \Vert \nabla \partial _t y \Vert _{L^2([0,T];L^2(\Omega ))}^2 \le C$ is controlled. Assuming $p=2$ for simplicity here, the second and third term can be controlled as

$$\begin{aligned}&\Big |\int _0^T\int _\Omega \Delta \partial _{t} y \cdot \Delta y \textrm{d}t \textrm{d}x\Big | \le C\Vert \nabla \partial _{t} y\Vert _{ \in L^2([0,T];L^2(\Omega ))} \Vert \nabla \Delta y\Vert _{ L^2([0,T];L^2(\Omega ))}\\&\le C\Vert \nabla \Delta y\Vert _{ L^2([0,T];L^2(\Omega ))}, \\&\Big |\int _0^T\int _\Omega \Delta ( \Delta y) \cdot \Delta y\textrm{d}t \textrm{d}x\Big | \ge \tfrac{1}{C}\Vert \nabla \Delta y\Vert ^2_{ L^2([0,T];L^2(\Omega ))}. \end{aligned}$$

This allows to obtain the control $\nabla \Delta y \in L^2([0,T];L^2(\Omega ))$ which suffices to give sense to the term $\nabla (D{H}(\Delta y ))$ in the weak formulation. Again, on a rigorous level, this test is performed for the regularized problem (1.4) with $\varepsilon$-independent bounds, and then the regularity for *y* is obtained in the limit of vanishing regularization $\varepsilon \rightarrow 0$, see Proposition 3.11 and Lemma 3.12 for details. More precisely, for given $p>d$, the additional regularity reads as $(1+|\Delta y|)^\frac{p-2}{2}|\nabla \Delta y|^2\in L^2([0,T];L^2(\Omega ))$, see Theorem 2.5. Even in the nonlinear case of $p\ne 2$, the regularity estimates introduced here rely on the theory for the Laplace operator only and as such are *independent of nonlinear regularity techniques*. This seems to be a special feature of the fourth order *p*-Laplacian, which was observed by the authors very much to their surprise. Up to their knowledge, it has not been used before.

Besides being crucial for our proof, the result might be of independent interest and improves the known regularity properties also for results in the quasi-static case ($\rho =0$) [2, 37] or in the isothermal case [26]. It seems that even in the static case of elastic minimizers this extra regularity property has not been shown previously. Let us mention, however, that compared to [2, 26, 37] the regularity issues force us to impose Dirichlet conditions on the *entire* boundary $\partial \Omega$.

Let us mention that our existence result on weak solutions only guarantees nonnegativity of the temperature. We expect the third law of thermodynamics to hold as well, i.e., the temperature cannot reach absolute zero. To the best of our knowledge, results on positivity of the temperature in fully nonlinear coupled systems of thermoviscoelasticity are scant, see [4] for a first result in the setting without acceleration and references therein. In the present context, this issue is currently open and deferred to future research.

The plan of the paper is as follows. Section 2 introduces the nonlinear model and states our main results. Then, the results are proved in Sections 3–5. We start by considering the $\varepsilon$-regularized problem and introduce a discretized solution with time stepping $\tau$ of the parabolic approximation with time delay $h>0$. This introduces a three layer approximation, and we successively pass to the limits in the layers, namely first in $\tau$ (Section 3), then in *h* (Section 4), and eventually in the regularization $\varepsilon$ (Section 5). It is important to mention that all essential a priori bounds are already established on the $\tau$-level in Section 3 and transfer over to the limits $\tau \rightarrow 0$, $h \rightarrow 0$, and $\varepsilon \rightarrow 0$. Moreover, some additional higher-order bounds based on elliptic regularity theory are provided (see Lemma 3.12) that blow up in the limit $\varepsilon \rightarrow 0$. Still, they are crucial to perform the final limiting passage in the heat-transfer equation to control some $\varepsilon$-dependent terms resulting from the regularization, see Proposition 5.4 for details.

## The model and main results

### Notation

Denoting by $d \in \{2, 3\}$ the dimension, we indicate by $\Omega \subset \mathbb {R}^d$ an open bounded set with $C^5$-boundary and fix $p \in (d, 2^*)$, where $2^* = \infty$ for $d=2$ and $2^* = 6$ for $d=3$. In what follows, we use standard notation for Lebesgue, Sobolev, and Bochner spaces. By $1\!\!1_J$ we denote the indicator function of a set $J \subset \mathbb {R}$ or $J \subset \Omega$. The lower index  means nonnegative elements, i.e., $L^2_+(\Omega )$ denotes the convex cone of nonnegative functions belonging to $L^2(\Omega )$ and a similar definition is used for $H^1_+(\Omega )$. Mean integrals are denoted by . We also set $\mathbb {R}_+:=[0,+\infty )$. Let $a \wedge b:=\min \{a, b\}$ for $a,b \,\in \mathbb {R}$. Moreover, we let $\textbf{Id}\in \mathbb {R}^{d \times d}$ be the identity matrix, and $\textbf{id}(x) :=x$ stands for the identity map on $\mathbb {R}^d$. We define the subsets $SO(d) :=\{A \in \mathbb {R}^{d \times d} :A^T A = \textbf{Id}, \, \det A = 1 \}$, $GL^+(d) :=\{F \in \mathbb {R}^{d \times d} :\det (F) > 0\}$, and $\mathbb {R}^{d \times d}_\textrm{sym}:=\{A \in \mathbb {R}^{d \times d} :A^T = A\}$. Furthermore, for a matrix $F \in \mathbb {R}^{d \times d}$ we write $F^{-T} :=(F^{-1})^T=(F^T)^{-1}$, and given a tensor *G* (of arbitrary dimension and order), $|G|$ will denote its Frobenius norm. We write the scalar product between vectors and matrices as $\cdot$ and  : , respectively. The tensor product of two vectors $v_1,v_2 \in \mathbb {R}^d$ is denoted by $v_1 \otimes v_2 \in \mathbb {R}^{d \times d}$. As usual, in the proofs generic constants *C* are strictly positive and may vary from line to line. If not stated otherwise, all constants only depend on *d*, *p*, $\Omega$, and the potentials and data defined in Subsection 2.2 below. We frequently use a scaled version of Young’s inequality with constant $\lambda \in (0,1)$ by which we mean $ab \le \lambda a^q + C_\lambda b^{q'}$ for $a, b \,\ge 0$, exponents $q, \, q' > 1$ with $1/q + 1/q' = 1$, and a suitable constant $C_\lambda >0$.

For $\Omega \subset \mathbb {R}^d$ and *p* as above, we introduce the set of *admissible deformations* by

2.1 $$\begin{aligned} \mathcal {Y}_{\textbf{id}}:=\{ y \in W^{2, p}(\Omega ; \mathbb {R}^d) :y = \textbf{id}\text { on } \partial \Omega , \, \det (\nabla y) > 0 \text { in } \Omega \}, \end{aligned}$$

and we say that the *absolute temperature* $\theta$ is admissible if $\theta \in L^1_+(\Omega )$.

### Energies and their respective potentials

The variational setting described in the sequel mostly coincides with the one from [2], up to a more special choice of the strain-gradient energy. In the following, let $C_0\ge 1$ be some fixed positive constant.

**Mechanical energy and coupling energy:** The *elastic energy* $\mathcal {W}^{\textrm{el}}:\mathcal {Y}_{\textbf{id}}\rightarrow \mathbb {R}_+$ is given by

2.2 $$\begin{aligned} \mathcal {W}^{\textrm{el}}(y) :=\int _\Omega W^{\textrm{el}}(\nabla y) \textrm{d}x, \end{aligned}$$

where $W^{\textrm{el}}:GL^+(d) \rightarrow \mathbb {R}_+$ is a frame indifferent elastic energy potential with the usual assumptions in nonlinear elasticity. More precisely, we require:

1. $$W^{\textrm{el}}$$ is $$C^2$$;
2. Frame indifference: $W^{\textrm{el}}(QF) = W^{\textrm{el}}(F)$ for all $F \in GL^+(d)$ and $Q \in SO(d)$;
3. Lower bound: $W^{\textrm{el}}(F) \ge \frac{1}{C_0} \big (|F|^2 + \det (F)^{-q}\big ) - C_0$ for all $F \in GL^+(d)$, where $q \ge \frac{pd}{p-d}$.

Adopting the concept of 2nd-grade nonsimple materials, see [49, 50], we also consider a *strain-gradient energy term* $\mathcal {H}:\mathcal {Y}_{\textbf{id}}\rightarrow \mathbb {R}_+$, defined as

2.3 $$\begin{aligned} \mathcal {H}(y) :=\int _\Omega H(\Delta y) \textrm{d}x. \end{aligned}$$

Here, $H:\mathbb {R}^{d} \rightarrow \mathbb {R}_{+}$ is of the form

2.4 $$\begin{aligned} H(v) = \mathfrak {h}( |v| ) \end{aligned}$$

for $v \in \mathbb {R}^d$, where $\mathfrak {h}:\mathbb {R}_+ \rightarrow \mathbb {R}_+$ is defined as

2.5 $$\begin{aligned} \mathfrak {h}(s) :=\int _{0}^{s} \max \{2\sigma , p\sigma ^{p-1}\} \textrm{d}\sigma . \end{aligned}$$

The definition of $\mathfrak {h}$ ensures that *H* is uniformly convex and has *p*-growth. More precisely, we have

1. $H$ is uniformly convex and $C^1$;
2. Frame indifference: $H(Q \Delta y) = H(\Delta y)$ in $\Omega$ for all $y \in \mathcal {Y}_{\textbf{id}}$ and $Q \in SO(d)$;
3. $|v|^p \le H(v) \le C_0|v|^p$ and $| D H (v)| \le C_0|v|^{p-1}$ for all $v \in \mathbb {R}^{d}$,

where $DH(v) :=(\partial _{v_i} H(v))_{i=1}^d = \max \{2, p |v|^{p-2}\} v$ is the gradient of *H* with respect to *v*. The *mechanical energy* $\mathcal {M}:\mathcal {Y}_{\textbf{id}}\rightarrow \mathbb {R}_+$ is then defined as

2.6 $$\begin{aligned} \mathcal {M}(y) :=\mathcal {W}^{\textrm{el}}(y) + \mathcal {H}(y). \end{aligned}$$

Besides the mechanical energy, we introduce a *coupling energy* $\mathcal {W}^{\textrm{cpl}}:\mathcal {Y}_{\textbf{id}}\times L^1_+(\Omega ) \rightarrow \mathbb {R}$ given by

2.7 $$\begin{aligned} \mathcal {W}^{\textrm{cpl}}(y, \theta ) :=\int _\Omega W^{\textrm{cpl}}(\nabla y, \theta ) \textrm{d}x, \end{aligned}$$

where $W^{\textrm{cpl}}:GL^+(d) \times \mathbb {R}_+ \rightarrow \mathbb {R}$ describes mutual interactions of mechanical and thermal effects, and satisfies

1. $W^{\textrm{cpl}}$ is continuous, and $C^2$ in $GL^+(d) \times (0, \infty )$;
2. $W^{\textrm{cpl}}(QF, \theta ) = W^{\textrm{cpl}}(F, \theta )$ for all $F \in GL^+(d)$, $\theta \ge 0$, and $Q \in SO(d)$;
3. $$W^{\textrm{cpl}}(F, 0) = 0$$ for all $$F \in GL^+(d)$$;
4. $|W^{\textrm{cpl}}(F,\theta ) - W^{\textrm{cpl}}(\tilde{F}, \theta )| \le C_0(1 + |F| + |\tilde{F}|)|F - \tilde{F}|$ for all $F, \, \tilde{F} \in GL^+(d)$, and $\theta \ge 0$;
5. For all $F \in GL^+(d)$ and $\theta > 0$ it holds that
  $$\begin{aligned} | \partial _{FF}^2 W^\textrm{cpl}(F,\theta )|&\le C_0, |\partial _{F\theta }^2W^{\textrm{cpl}}(F, \theta )| \le \frac{C_0(1+|F|)}{\max \lbrace \theta ,1\rbrace }, \\ \frac{1}{C_0}&\le -\theta \partial _{\theta \theta }^2 W^{\textrm{cpl}}(F, \theta ) \le C_0. \end{aligned}$$

Notice that, by (C.3) and the second bound in (C.5), $\partial _F W^{\textrm{cpl}}$ can be continuously extended to zero temperatures with $\partial _F W^\textrm{cpl}(F, 0) = 0$. For $F \in GL^+(d)$ and $\theta \ge 0$, we define the *total free energy potential*

2.8 $$\begin{aligned} W(F, \theta ) :=W^{\textrm{el}}(F) + W^{\textrm{cpl}}(F, \theta ). \end{aligned}$$

**Dissipation potential:** The *dissipation functional* $\mathcal {R}:\mathcal {Y}_{\textbf{id}}\times H^1(\Omega ;\mathbb {R}^d) \times L^1_+(\Omega ) \rightarrow \mathbb {R}_+$ is defined as

2.9 $$\begin{aligned} \mathcal {R}( y, \tilde{y}, \theta ) :=\int _\Omega R(\nabla y, \nabla \tilde{y}, \theta ) \textrm{d}x, \end{aligned}$$

where $R:\mathbb {R}^{d \times d} \times \mathbb {R}^{d \times d} \times \mathbb {R}_+ \rightarrow \mathbb {R}_+$ is the *potential of dissipative forces* satisfying

1. $R(F, \dot{F}, \theta ) :=\frac{1}{2} D(C, \theta )[\dot{C}, \dot{C}] :=\frac{1}{2} \dot{C}: D(C, \theta ) \dot{C}$, where $C :=F^T F$, $\dot{C} :=\dot{F}^T F + F^T \dot{F}$, and $D \in C(\mathbb {R}^{d \times d}_\textrm{sym}\times \mathbb {R}_+; \mathbb {R}^{d \times d \times d \times d})$ with $D_{ijkl} = D_{jikl}= D_{klij}$ for $1 \le i,j,k,l \le d$;
2. $\frac{1}{C_0} |\dot{C}|^2 \le \dot{C}: D(C, \theta ) \dot{C} \le C_0|\dot{C}|^2$ for all $C, \, \dot{C} \in \mathbb {R}^{d \times d}_\textrm{sym}$ and $\theta \ge 0$.

Notice that Assumption (D.1) implies that the viscous stress $\partial _{\dot{F}} R(F, \dot{F}, \theta )$ is linear in the time derivative $\dot{C}$ as well as (see e.g. [2, (2.8)])

2.10 $$\begin{aligned} \partial _{\dot{F}} R(F, \dot{F}, \theta ) = 2 F D(C, \theta ) \dot{C}. \end{aligned}$$

We also define the associated *dissipation rate* $\xi :\mathbb {R}^{d \times d} \times \mathbb {R}^{d \times d} \times \mathbb {R}_+ \rightarrow \mathbb {R}_+$ as

2.11 $$\begin{aligned} \xi (F, \dot{F}, \theta ) :=\partial _{\dot{F}} R(F, \dot{F}, \theta ) : \dot{F} = 2 R(F, \dot{F},\theta ), \end{aligned}$$

where the second identity follows from (2.10) and Assumption (D.1), see also [2, (2.9)].

Below, for technical reasons explained in (1.4), we will also consider a *regularized version of the dissipation* $\mathcal {R}_\varepsilon :\mathcal {Y}_{\textbf{id}}\times H^3(\Omega ;\mathbb {R}^d) \times L^1_+(\Omega ) \rightarrow \mathbb {R}_+$, defined as

2.12 $$\begin{aligned} \mathcal {R}_\varepsilon ( y, \tilde{y}, \theta ) :=\int _\Omega R(\nabla y, \nabla \tilde{y}, \theta ) \textrm{d}x + \frac{\varepsilon }{2} \int _\Omega |\nabla \Delta \tilde{y}|^2 \textrm{d}x \end{aligned}$$

for a small regularization parameter $\varepsilon >0$.

**Heat conductivity:** The map $\mathbb {K}:\mathbb {R}_+ \rightarrow \mathbb {R}^{d \times d}_\textrm{sym}$ denotes the *heat conductivity tensor* of the material in the deformed configuration. We require that $\mathbb {K}$ is continuous, symmetric, uniformly positive definite, and bounded. More precisely, for all $\theta \ge 0$ it holds that

2.13 $$\begin{aligned} \frac{1}{C_0} \le \mathbb {K}(\theta ) \le C_0, \end{aligned}$$

where the inequalities are meant in the eigenvalue sense. We define the pull-back $\mathcal {K}:GL^+(d) \times \mathbb {R}_+ \rightarrow \mathbb {R}^{d \times d}_\textrm{sym}$ of $\mathbb {K}$ into the reference configuration by (see [37, (2.24)])

$$\begin{aligned} \mathcal {K}(F, \theta ) :=\det (F) F^{-1} \mathbb {K}(\theta ) F^{-T}. \end{aligned}$$

**Thermal energy and total internal energy:** Following [2, 4, 37], the *(thermal part of the) internal energy* $W^{\textrm{in}}:GL^+(d) \times (0, \infty ) \rightarrow \mathbb {R}$ is defined as

2.14 $$\begin{aligned} W^{\textrm{in}}(F, \theta ) :=W^{\textrm{cpl}}(F, \theta ) - \theta \partial _\theta W^{\textrm{cpl}}(F, \theta ). \end{aligned}$$

Using (C.3) and the third bound in (C.5), we see that $W^{\textrm{in}}$ can be continuously extended to zero temperatures by setting $W^{\textrm{in}}(F, 0) = 0$ for all $F \in GL^+(d)$. Furthermore, by the third bound in (C.5) we have that

$$\begin{aligned} \partial _{\theta } W^{\textrm{in}}(F, \theta ) = -\theta \partial _{\theta \theta }^2 W^{\textrm{cpl}}(F, \theta ) \in [C_0^{-1}, C_0] \qquad \text {for all }F \in GL^+(d)\text { and }\theta > 0. \end{aligned}$$

Along with (C.3) this shows that the internal energy is controlled by the temperature in the sense that

2.15 $$\begin{aligned} \frac{1}{C_0} \theta \le W^{\textrm{in}}(F, \theta ) \le C_0\theta . \end{aligned}$$

Finally, we define *total internal energy functional* $\mathcal {E}:\mathcal {Y}_{\textbf{id}}\times L^1_+(\Omega ) \rightarrow \mathbb {R}_+$ by

2.16 $$\begin{aligned} \mathcal {E}(y, \theta ) :=\mathcal {M}(y) + \mathcal {W}^\textrm{in}(y,\theta ) \qquad \text { with } \mathcal {W}^\textrm{in}(y,\theta ) :=\int _\Omega W^{\textrm{in}}(\nabla y, \theta ) \textrm{d}x. \end{aligned}$$

We remark that the above assumptions on the potentials coincide with the ones in [2, Section 2.1], up to the fact that, differently to [2, (2.4)], we only allow the potential of the strain-gradient energy to depend on the norm of the diagonal $\Delta y$ of $\nabla ^2 y$ (cf. formulas (2.3)–(2.4) above). Moreover, for $d=3$, the range of $p \in (3,6)$ is restricted as we need the Sobolev embedding $H^3(\Omega ;\mathbb {R}^d) \subset \subset W^{2,p}(\Omega ;\mathbb {R}^d)$. Eventually, in contrast to [2], in the definition of admissible deformations, see (2.1), we need to impose Dirichlet conditions on the *entire* boundary $\partial \Omega$ as this allows us to apply elliptic regularity results. We refer to [37, Examples 2.4 and 2.5] for a class of potentials satisfying all assumptions above.

### Equations of nonlinear thermoviscoelasticity with inertia: Existence of weak solutions

Let $I :=[0, T]$ where $T > 0$ denotes a time horizon, let $\rho > 0$ be a constant *mass density* in the reference configuration, let $\kappa \ge 0$ be a constant *heat-transfer coefficient*, let $f \in W^{1, 1}(I; L^2(\Omega ; \mathbb {R}^d))$ be a time-dependent *dead load*, and let $\theta _\flat \in W^{1, 1}(I; L^2_+(\partial \Omega ))$ be an *external temperature*. Moreover, let $\varepsilon \ge 0$ be a regularization parameter, where $\varepsilon = 0$ corresponds to the setting without regularization. In the strong form, we study the system

2.17a $$\begin{aligned} f&= \rho \partial ^{2}_{tt} y - \textrm{div} \big ( \partial _{F} W ( \nabla y, \theta ) + \partial _{\dot{F}} R(\nabla y, \partial _{t} \nabla y, \theta ) \nonumber \\&\quad - \nabla ({D H}(\Delta y)) + \varepsilon \partial _t \nabla \Delta ^{2}y \big ) \,,\end{aligned}$$

2.17b $$\begin{aligned}&-\theta \partial ^{2}_{\theta \theta } W^\textrm{cpl} (\nabla y, \theta ) \partial _{t} \theta = \textrm{div} \big ( \mathcal {K} (\nabla y, \theta ) \nabla \theta \big ) + \xi (\nabla y, \partial _{t} \nabla y, \theta ) \nonumber \\&\quad + \theta \partial ^{2}_{F \theta } W^\textrm{cpl} (\nabla y, \theta ) : \partial _{t} \nabla y + \varepsilon |\partial _t \nabla \Delta y|^2 \,, \end{aligned}$$

coupled with the boundary conditions

2.18a $$\begin{aligned} y = \textbf{id}&\qquad \text {in } I \times \partial \Omega , \end{aligned}$$

2.18b $$\begin{aligned} {D H}(\Delta y) = 0&\qquad \text {in } I \times \partial \Omega ,\end{aligned}$$

2.18c $$\begin{aligned} \varepsilon \partial _\nu \Delta y = \varepsilon \Delta ^2 y = 0&\qquad \text {in } I \times \partial \Omega , \end{aligned}$$

2.18d $$\begin{aligned} \mathcal {K}(\nabla y, \theta ) \nabla \theta \cdot \nu + \kappa \theta = \kappa \theta _\flat&\qquad \text {in } I\times \partial \Omega \end{aligned}$$

and subject to the initial conditions

2.19 $$\begin{aligned} y(0) = y_0, \qquad \partial _t y(0) = y_0', \qquad \theta (0) = \theta _0, \end{aligned}$$

for initial values $y_0 \in \mathcal {Y}_{\textbf{id}}$, $y_0' \in H^1_0(\Omega ; \mathbb {R}^d)$, and $\theta _0 \in L^2_+ (\Omega )$. We refer to [37, Section 2] for a thorough explanation of this model. We highlight that, compared to [37], we include inertial effects, i.e., the mechanical equation features the term $\rho \partial ^{2}_{tt} y$. Moreover, for $\varepsilon >0$ there are regularizing terms both in (2.17a) and (2.17b), complemented with the additional natural boundary condition (2.18c). In the regularized setting, we will assume stronger initial conditions for the deformations, namely $y_{0,\varepsilon } \in \mathcal {Y}^\textrm{reg}_{\textbf{id}}$ and $y'_{0,\varepsilon }\in H^{3}(\Omega ; \mathbb {R}^{d}) \cap H^1_0(\Omega ;\mathbb {R}^d)$, where

2.20 $$\begin{aligned} \mathcal {Y}^\textrm{reg}_{\textbf{id}}:=\big \{ y \in \mathcal {Y}_{\textbf{id}}\cap H^4(\Omega ;\mathbb {R}^d) :\partial _\nu \Delta y(t) = \Delta y(t) = 0 \mathcal {H}^{d-1}\text {-a.e. in } \partial \Omega \big \}. \end{aligned}$$

We now first treat the case $\varepsilon >0$ and afterwards we address the system without regularization.

### Existence of weak solutions for the regularized system

We introduce the notion of weak solutions related to (2.17a)–(2.18d) for $\varepsilon >0$. Following the lines of [37, (2.28)–(2.29)], one can show that (2.17a) together with (2.18a)–(2.18c) is equivalent to the weak formulation (2.21) below. Besides the regularizing term, the only difference in (2.17a) compared to [37] is the presence of the inertial term. Arguing as in [37, (2.16)–(2.17)], we can rewrite the heat-transfer equation (2.17b) in terms of the internal energy $w_\varepsilon$ as

$$\begin{aligned} \partial _{t} w_\varepsilon = \textrm{div} \big ( \mathcal {K} (\nabla y_\varepsilon , \theta _\varepsilon ) \nabla \theta _\varepsilon \big ) + \xi (\nabla y_\varepsilon , \partial _{t} \nabla y_\varepsilon , \theta _\varepsilon ) +\varepsilon |\partial _t \nabla \Delta y_\varepsilon |^2 + \partial _{F} W^\textrm{cpl} (\nabla y_\varepsilon , \theta _\varepsilon ) : \partial _{t} \nabla y_\varepsilon , \end{aligned}$$

where we have used (2.14) and the identity

$$\begin{aligned} \partial _{t} w_\varepsilon = \partial _{F} W^\textrm{cpl} (\nabla y_\varepsilon , \theta _\varepsilon ) : \partial _{t} \nabla y_\varepsilon - \theta _\varepsilon \partial _{F\theta }^2 W^\textrm{cpl} (\nabla y_\varepsilon , \theta _\varepsilon ) : \partial _{t} \nabla y_\varepsilon - \theta _\varepsilon \partial ^{2}_{\theta \theta } W^\textrm{cpl} (\nabla y_\varepsilon , \theta _\varepsilon ) \partial _{t} \theta _\varepsilon \,. \end{aligned}$$

Taking also (2.18d) into account, this yields the following weak formulation.

#### Definition 2.1

*(Weak solutions to the regularized thermo-elastodynamic system for viscous solids)* Let $y_{0,\varepsilon } \in \mathcal {Y}^\textrm{reg}_{\textbf{id}}$, $y'_{0,\varepsilon }\in H^{3}(\Omega ; \mathbb {R}^{d}) \cap H^1_0(\Omega ;\mathbb {R}^d)$, $\theta _{0} \in L^{2}_{+}(\Omega )$, $f \in W^{1, 1}(I; L^{2}(\Omega ; \mathbb {R}^{d}))$, and $\theta _\flat \in W^{1, 1}(I; L^2_+(\partial \Omega ))$. We say that a pair $(y_\varepsilon , \theta _\varepsilon )$ with

$$\begin{aligned} y_\varepsilon&\in L^{\infty }( I ; \mathcal {Y}_{\textbf{id}}) \cap H^1\big (I;H^3(\Omega ;\mathbb {R}^d) \big ) \cap H^2\big (I; (H^3(\Omega ;\mathbb {R}^d) \cap H^1_0(\Omega ;\mathbb {R}^d))^*\big ) , \\ \theta _\varepsilon&\in L^2(I; H^{1}_+(\Omega )) \end{aligned}$$

is a solution to the regularized thermo-elastodynamic system with initial conditions $(y_{0,\varepsilon }, y'_{0,\varepsilon }, \theta _{0})$ if $y_\varepsilon (0) = y_{0,\varepsilon }$, $\partial _t y_\varepsilon (0) = y_{0,\varepsilon }'$, the internal energy $w_\varepsilon :=W^\textrm{in} (\nabla y_\varepsilon , \theta _\varepsilon )$ lies in $L^2(I; H^1(\Omega )) \cap H^1(I; (H^1(\Omega ))^*)$ and satisfies $w_\varepsilon (0) = w_{0,\varepsilon } :=W^\textrm{in}(\nabla y_{0,\varepsilon }, \theta _0)$, and the following equations are satisfied for every $z \in C^{\infty } ( I \times \overline{\Omega }; \mathbb {R}^{d})$ with $z = 0$ on $I \times \partial \Omega$ and for every $\varphi \in C^{\infty } ( I\times \overline{\Omega })$:

2.21 $$\begin{aligned}&\begin{aligned} 0=&\int _I \int _{\Omega } \partial _{F} W(\nabla y_\varepsilon , \theta _\varepsilon ) : \nabla z \textrm{d}x \textrm{d}t \\&+ \int _I \int _{\Omega } {D H} ( \Delta y_\varepsilon ) \cdot \Delta z \textrm{d}x \textrm{d}t + \varepsilon \int _I \int _{\Omega } \partial _t \nabla \Delta y_\varepsilon : \nabla \Delta z \textrm{d}x \textrm{d}\\&\quad + \int _I \int _{\Omega } \partial _{\dot{F}} R(\nabla y_\varepsilon , \partial _t \nabla y_\varepsilon , \theta _\varepsilon ) : \nabla z \textrm{d}x \textrm{d}t \\&+ \rho \int _I \langle \partial ^2_{tt} y_\varepsilon , z \rangle \textrm{d}t - \int _I \int _{\Omega } f \cdot z \textrm{d}x \textrm{d}t \,, \end{aligned} \end{aligned}$$

2.22 $$\begin{aligned}&\begin{aligned} 0 =&\int _I \int _\Omega \mathcal {K}(\nabla y_\varepsilon , \theta _\varepsilon ) \nabla \theta _\varepsilon \cdot \nabla \varphi \textrm{d}x \textrm{d}t + \int _I \langle \partial _t w_\varepsilon , \varphi \rangle \textrm{d}t \\&- \kappa \int _I \int _{\partial \Omega } (\theta _\flat - \theta _\varepsilon ) \varphi \textrm{d}\mathcal {H}^{d-1} \textrm{d}t \\&\quad - \int _I \int _\Omega \Big ( \xi (\nabla y_\varepsilon , \partial _t \nabla y_\varepsilon , \theta _\varepsilon ) ) + \partial _F W^{\textrm{cpl}}(\nabla y_\varepsilon , \theta _\varepsilon ) : \partial _t \nabla y_\varepsilon + \varepsilon |\partial _t \nabla \Delta y_\varepsilon |^2 \Big ) \varphi \textrm{d}x \textrm{d}t , \end{aligned} \end{aligned}$$

where $\langle \cdot , \cdot \rangle$ denotes the duality pairing of $H^3(\Omega ;\mathbb {R}^d) \cap H^1_0(\Omega ;\mathbb {R}^d)$ and its dual or of $H^1(\Omega )$ and its dual, respectively.

By a density result, one could choose the test functions also in the larger spaces $L^2(I; H^3(\Omega ;\mathbb {R}^d) \cap H^1_0(\Omega ;\mathbb {R}^d))$ (for *z*) and $L^2(I; H^1(\Omega )\cap C^0(\Omega ))$ (for $\varphi$) The first main results of the paper read as follows.

#### Theorem 2.2

(Existence of weak solutions to the regularized system) Let $p \in (2, +\infty )$ if $d=2$ or $p \in (3, 6)$ for $d=3$. Assume that (W.1)–(W.3), (H.1)–(H.3), (C.1)–(C.5), (D.1)–(D.2), and (2.13) hold true. Let $\varepsilon >0$. Let $y_{0,\varepsilon } \in \mathcal {Y}^\textrm{reg}_{\textbf{id}}$, $y'_{0,\varepsilon }\in H^{3}(\Omega ; \mathbb {R}^{d}) \cap H^{1}_0(\Omega ; \mathbb {R}^{d})$, $\theta _{0} \in L^{2}_{+}(\Omega )$, $f \in W^{1, 1}( I; L^{2}(\Omega ; \mathbb {R}^{d}))$, and $\theta _\flat \in W^{1, 1}( I; L^2_+(\partial \Omega ))$. Then, there exists a weak solution $(y_\varepsilon , \theta _\varepsilon )$ to the regularized thermo-elastodynamic system with initial data $(y_{0,\varepsilon }, y'_{0,\varepsilon }, \theta _{0})$ in the sense of Definition 2.1.

For weak solutions, we can derive energy balances and some regularity properties. Recall (2.6) and (2.16).

#### Theorem 2.3

(Regularity of solutions and total energy balance) In the setting of Theorem 2.2, we find weak solutions $(y_\varepsilon , \theta _\varepsilon )$ satisfying $y_{\varepsilon } \in L^\infty (I; \mathcal {Y}^\textrm{reg}_{\textbf{id}})$. Moreover, for each $t \in I$, the system satisfies the mechanical energy balance

2.23 $$\begin{aligned} \mathcal {M}(y_\varepsilon (t))&+ \frac{\rho }{2} \Vert \partial _t y_\varepsilon (t) \Vert ^2_{L^2(\Omega )} + \int _0^t \int _\Omega \nonumber \\&\quad \Big ( \xi (\nabla y_\varepsilon , \partial _t \nabla y_\varepsilon , \theta _\varepsilon ) ) + \varepsilon |\partial _t \nabla \Delta y_\varepsilon |^2 \textrm{d}x + \partial _{F} W^\textrm{cpl} (\nabla y_{\varepsilon }, \theta _{\varepsilon }) : \partial _{t}\nabla y_{\varepsilon } \Big ) \textrm{d}s \nonumber \\&= \mathcal {M}(y_{0,\varepsilon }) + \frac{\rho }{2} \Vert y_{0,\varepsilon }' \Vert ^2_{L^2(\Omega )} + \int _0^t \int _\Omega f \cdot \partial _{t} y_{\varepsilon } \textrm{d}x \textrm{d}s , \end{aligned}$$

the thermal energy balance

2.24 $$\begin{aligned} \int _\Omega w_\varepsilon (t) \textrm{d}x&= \int _\Omega w_{0,\varepsilon } \textrm{d}x + \int _0^t \int _\Omega \nonumber \\&\Big ( \xi (\nabla y_\varepsilon , \partial _t \nabla y_\varepsilon , \theta _\varepsilon ) + \varepsilon |\partial _t \nabla \Delta y_\varepsilon |^2 + \partial _F W^{\textrm{cpl}}(\nabla y_\varepsilon , \theta _\varepsilon ) : \partial _t \nabla y_\varepsilon \Big ) \textrm{d}x \textrm{d}s\nonumber \\&\quad + \kappa \int _0^t \int _{\partial \Omega } (\theta _\flat - \theta _\varepsilon ) \textrm{d}\mathcal {H}^{d-1} \textrm{d}s \,, \end{aligned}$$

and the total energy balance

2.25 $$\begin{aligned} \begin{aligned}&\mathcal {E}(y_\varepsilon (t), \theta _\varepsilon (t)) + \frac{\rho }{2} \Vert \partial _t y_\varepsilon (t) \Vert ^2_{L^2(\Omega )} \\&\quad = \mathcal {E}(y_{0,\varepsilon },\theta _0) + \frac{\rho }{2} \Vert y_{0,\varepsilon }' \Vert ^2_{L^2(\Omega )} + \int _0^t \int _{\partial \Omega }\kappa (\theta _\flat -\theta _\varepsilon )\, \textrm{d}\mathcal {H}^{d-1} \textrm{d}s + \int _0^t\int _\Omega f\cdot \partial _t y_\varepsilon \textrm{d}x \textrm{d}s . \end{aligned} \end{aligned}$$

We emphasize that the energy balances are well-defined pointwise for each $t \in I$ since the regularity of the deformation and the temperature imply $y_\varepsilon \in C(I;W^{2,p}(\Omega ;\mathbb {R}^d))$, $\partial _t y_\varepsilon \in C(I;L^2(\Omega ;\mathbb {R}^d))$, and $w_\varepsilon \in C(I;L^2(\Omega ))$, where we use that $p < 2^*$ and [42, Lemma 7.3]. The total energy balance (2.25) arises by summing (2.23) and (2.24). In particular, we observe that the system is closed for $\kappa =0$ and $f = 0$. Still, an exchange of mechanical energy and thermal energy is possible due to the (regularized) *dissipation rate* $\xi (\nabla y_\varepsilon , \partial _t \nabla y_\varepsilon , \theta _\varepsilon ) ) + \varepsilon |\partial _t \nabla \Delta y_\varepsilon |^2$ and the *adiabatic heat source* $\partial _{F} W^\textrm{cpl} (\nabla y_{\varepsilon }, \theta _{\varepsilon }) : \partial _{t}\nabla y_{\varepsilon }$ which cancel out in the summation of (2.23) and (2.24).

### Existence of weak solutions for the system without regularization

Our goal is to remove the regularization by passing to the limit $\varepsilon \rightarrow 0$ for weak solutions $(y_\varepsilon ,\theta _\varepsilon )$ in the sense of Definition 2.1. Unfortunately, the available a priori bounds and compactness results yielding a limit $(y,\theta )$, see Lemma 5.1 below, are not strong enough as they guarantee convergence of all terms in (2.21)–(2.22) except for the acceleration $\partial ^2_{tt} y_\varepsilon$ in (2.21) and the dissipation rate $\xi (\nabla y_\varepsilon , \partial _t \nabla y_\varepsilon , \theta _\varepsilon )$ in (2.22). Accordingly, also the validity of the mechanical and thermal energy balances (2.23)–(2.24) cannot be expected in the limit $\varepsilon \rightarrow 0$ as

$$\begin{aligned} \liminf _{\varepsilon \rightarrow 0} \int _0^t \int _\Omega \xi (\nabla y_\varepsilon , \partial _t \nabla y_\varepsilon , \theta _\varepsilon ) \textrm{d}x \textrm{d}s > \int _0^t \int _\Omega \xi (\nabla y, \partial _t \nabla y, \theta ) \textrm{d}x \textrm{d}s \end{aligned}$$

is possible under the available compactness results. In [2, Lemma 4.5] and [37, Propositions 5.1 and 6.6], equality was guaranteed by a chain rule for the mechanical energy (see [37, Proposition 3.6]) which also allowed to derive a mechanical energy balance. Due to the presence of the inertial term, it appears to be impossible to adapt this strategy to the current setting.

We overcome this difficulty by appealing to a weaker formulation of the mechanical and the heat-transfer equation, see also [24, 41] for a similar approach. In (2.21), it suffices to perform an integration by parts in time to deal with the term $\partial ^2_{tt} y_\varepsilon$. The passage to a weaker form of (2.22) is based on the observation that the delicate dissipation term cancels in the summation of (2.23) and (2.24). More precisely, this passage is achieved by testing (2.21) with $z = \partial _{t} y \varphi$ and adding the result to (2.22). This leads to the following notion of weak solution, whose form will be explained in more detail by a formal computation in (2.29)–(2.33) below.

#### Definition 2.4

*(Weak solutions to thermo-elastodynamic system for viscous solids)* Let $y_{0} \in \mathcal {Y}_{\textbf{id}}$, $y'_{0}\in H^{1}(\Omega ; \mathbb {R}^{d})$, $\theta _{0} \in L^{2}_{+}(\Omega )$, $f \in W^{1, 1}( I; L^{2}(\Omega ; \mathbb {R}^{d}))$, and $\theta _\flat \in W^{1, 1}( I; L^2_+(\partial \Omega ))$. We say that a pair $(y, \theta )$ with

$$\begin{aligned}&y \in L^{\infty }( I ; \mathcal {Y}_{\textbf{id}}) \cap H^1(I;H^1(\Omega ;\mathbb {R}^d) ), \quad \theta \in L^1(I; W^{1, 1}_+(\Omega )),\\&\quad DH(\Delta y)\in L^2(I;W^{1,6/5}(\Omega ; \mathbb {R}^{d})) \end{aligned}$$

is a solution to the thermo-elastodynamic system with initial conditions $(y_0, y'_0, \theta _{0})$ if the following equations are satisfied for every $z \in C^{\infty } ( I\times \overline{\Omega }; \mathbb {R}^{d})$ with $z = 0$ on $I\times \partial \Omega$ and $z(T) = 0$, and for every $\varphi \in C^{\infty } ( I\times \overline{\Omega })$ with $\varphi (T) = 0$:

2.26 $$\begin{aligned} 0=&\int _I \int _{\Omega } \partial _{F} W(\nabla y, \theta ) : \nabla z \textrm{d}x \textrm{d}t + \int _I \int _{\Omega } {D H} ( \Delta y) \cdot \Delta z \textrm{d}x \textrm{d}t \nonumber \\&\quad + \int _I \int _{\Omega } \partial _{\dot{F}} R(\nabla y, \partial _t \nabla y, \theta ) : \nabla z \textrm{d}x \textrm{d}t - \rho \int _I \int _{\Omega } \partial _t y \cdot \partial _t z \textrm{d}x \textrm{d}t \nonumber \\&- \int _I \int _{\Omega } f \cdot z \textrm{d}x \textrm{d}t - \rho \int _{\Omega } y'_{0} \cdot z(0) \textrm{d}x \,, \end{aligned}$$

2.27 $$\begin{aligned} 0=&\int _I\int _\Omega \mathcal {K}(\nabla y, \theta ) \nabla \theta \cdot \nabla \varphi \textrm{d}x \textrm{d}t - \kappa \int _I \int _{\partial \Omega } (\theta _\flat -\theta ) \varphi \textrm{d}\mathcal {H}^{d-1} \textrm{d}t - \int _I\int _\Omega \varphi \, f \cdot \partial _{t} y \textrm{d}x \textrm{d}t \nonumber \\&- \int _I \int _\Omega \big ( W^{\textrm{el}}(\nabla y ) + H(\Delta y ) + w + \frac{\rho }{2} |\partial _t y|^2 \big ) \partial _{t} \varphi \textrm{d}x \textrm{d}t \nonumber \\&- \int _{\Omega } \big ( W^{\textrm{el}}(\nabla y_{0}) + H(\Delta y_{0} ) + w_0 + \frac{\rho }{2} |y_0'|^2 \big ) \varphi (0) \textrm{d}x \nonumber \\&\quad +\int _I\int _\Omega \Big ( \partial _F W (\nabla y , \theta ) + \partial _{\dot{F}} R (\nabla y , \nabla \partial _{t} y , \theta ) \Big ) : ( \partial _{t} y \otimes \nabla \varphi ) \textrm{d}x \textrm{d}t \nonumber \\&\quad - \int _I\int _\Omega D{H}(\Delta y) \cdot \partial _{t} y \Delta \varphi \, \textrm{d}x \textrm{d}t - 2 \int _I\int _\Omega \nabla (D{H}(\Delta y )) : ( \partial _{t}y \otimes \nabla \varphi ) \textrm{d}x \textrm{d}t , \end{aligned}$$

where for shorthand we set $w :=W^\textrm{in} (\nabla y, \theta )$ and $w_0 :=W^\textrm{in} (\nabla y_0, \theta _0 )$.

By a density result, one could choose the test functions also in the larger spaces $L^2(I; W^{2,p}(\Omega ;\mathbb {R}^d)\cap H^1_0(\Omega ;\mathbb {R}^d)) \cap H^1(I;L^s(\Omega ;\mathbb {R}^d))$ (for *z*) and $C^0(I;C^2(\Omega )) \cap H^{1}(I; C^0(\Omega ))$ (for $\varphi$), where $s >1$ for $d=2$ and $s =\frac{6}{5}$ for $d=3$.

In particular, we observe that, due to the lack of regularity of $\partial ^2_{tt} y$ and $\partial _{t} w$, the initial conditions of $\partial _t y$ and *w* are given implicitly in a weak form, relying on an integration by parts in time. An important aspect of the weak formulation (2.27) is that it directly guarantees the total energy balance. Indeed, for each $t \in I$ such that(and thus for a.e. $t \in I$), we can test (2.27) with $\varphi$ given by $\varphi \equiv 1$ on $(0,t-\delta )$, $\varphi \equiv 0$ on $(t+\delta ,T)$ and $\varphi ' \equiv -\frac{1}{2\delta }$ on $(t-\delta , t + \delta )$. In the limit $\delta \rightarrow 0$, after rearrangement, this yields

2.28 $$\begin{aligned} \int _\Omega&\big ( W^{\textrm{el}}(\nabla y(t) ) + H(\Delta y(t) ) + w(t) + \frac{\rho }{2} |\partial _t y(t)|^2 \big ) \textrm{d}x \nonumber \\&= \int _{\Omega } \big ( W^{\textrm{el}}(\nabla y_{0}) + H(\Delta y_{0} ) + w_0 + \frac{\rho }{2} |y_0'|^2 \big ) \textrm{d}x + \kappa \int _0^t \int _{\partial \Omega } (\theta _\flat -\theta ) \textrm{d}\mathcal {H}^{d-1} \textrm{d}s \nonumber \\&+ \int _0^t \int _\Omega f \cdot \partial _{t} y \textrm{d}x \textrm{d}s \,, \end{aligned}$$

which is exactly the total energy balance, cf. also (2.25). Whereas a total energy balance still holds, the respective form of (2.23) and (2.24) may become *inequalities*. In some sense, this weaker form based on a replacement is inspired by fluid-mechanics for compressible heat conduction fluids, where the conservation of the total energy is guaranteed by transferring the heat equation into an inequality for the entropy [22, 23], see also [24, 41] for a similar approach.

#### Theorem 2.5

(Existence and regularity of weak solutions) Let $p \in (2, +\infty )$ if $d=2$ or $p \in (3, 6)$ for $d=3$. Assume that (W.1)–(W.3), (H.1)–(H.3), (C.1)–(C.5), (D.1)–(D.2), and (2.13) hold true. Let $y_{0} \in \mathcal {Y}_{\textbf{id}}$, $y'_{0}\in H^{1}_{0}(\Omega ; \mathbb {R}^{d})$, $\theta _{0} \in L^{2}_{+}(\Omega )$, $f \in W^{1, 1}( I ; L^{2}(\Omega ; \mathbb {R}^{d}))$, and $\theta _\flat \in W^{1, 1}( I ; L^2_+(\partial \Omega ))$. Then, there exists a weak solution $(y, \theta )$ to the thermo-elastodynamic system with initial data $(y_{0}, y'_{0}, \theta _{0})$ in the sense of Definition 2.4. The weak solution satisfies $y\in L^2(I; H^3(\Omega ;\mathbb {R}^d))$ and $(1+|\Delta y|)^\frac{p-2}{2}|\nabla \Delta y|^2\in L^2(I\times \Omega )$.

Note that (2.28) holds and (2.18b) is satisfied in the sense of traces.

### Formal derivation of the weak formulation

Let us close this section with a formal derivation of equation (2.27) which will be made precise below in Proposition 5.3 for the regularized system. Assuming sufficient regularity for *y* and $\theta$, let us check that the formulations in (2.27) and (2.22) (for $\varepsilon = 0$) coincide. First, an integration by parts in time shows that, for $\varepsilon = 0$, (2.22) is equivalent to

2.29 $$\begin{aligned} \begin{aligned} 0&= \int _I\int _\Omega \mathcal {K} (\nabla y, \theta ) \nabla \theta \cdot \nabla \varphi \, \textrm{d}x \textrm{d}t - \int _I\int _\Omega \big ( \xi (\nabla y, \partial _{t} \nabla y, \theta ) + \partial _{F} W^\textrm{cpl} (\nabla y, \theta ) : \partial _{t} \nabla y\big ) \varphi \, \textrm{d}x \textrm{d}t \\&\phantom {=}\quad + \kappa \int _{ I} \int _{\partial \Omega } (\theta - \theta _\flat ) \varphi \, \textrm{d}\mathcal {H}^{d-1} \textrm{d}t - \int _I\int _\Omega w \partial _{t} \varphi \textrm{d}x \textrm{d}t - \int _{\Omega } w_0 \varphi (0) \textrm{d}x \end{aligned} \end{aligned}$$

for every $\varphi \in C^{\infty } ( I \times \overline{\Omega })$ with $\varphi (T) = 0$. Now, we test (2.21) with $z:=\partial _{t} y \varphi$ for $\varphi \in C^{\infty } (I\times \overline{\Omega })$ with $\varphi (T) = 0$. Using (2.8), (2.11), expanding $\nabla ( \partial _{t} y \varphi )$, and rearranging the terms, we obtain

2.30 $$\begin{aligned} \begin{aligned}&- \int _I\int _\Omega \xi (\nabla y, \partial _{t} \nabla y, \theta ) \varphi \textrm{d}x \textrm{d}t - \int _I\int _\Omega \partial _{F} W^{\textrm{cpl}}(\nabla y, \theta ) : \partial _{t} \nabla y \varphi \textrm{d}x \textrm{d}t \\&\quad = \int _I\int _\Omega \partial _{F} W^{\textrm{el}}(\nabla y, \theta ) : \partial _{t} \nabla y \varphi \textrm{d}x \textrm{d}t + \int _I\int _\Omega \partial _{F} W(\nabla y, \theta ) : (\partial _{t} y \otimes \nabla \varphi ) \textrm{d}x \textrm{d}t \\&\phantom {=}\quad + \int _I\int _\Omega \partial _{\dot{F}} R(\nabla y, \partial _{t} \nabla y, \theta ) : (\partial _{t} y \otimes \nabla \varphi ) \textrm{d}x \textrm{d}t + \int _I\int _\Omega {D H}(\Delta y) \cdot \Delta (\partial _{t} y \varphi ) \textrm{d}x \textrm{d}t \\&\phantom {=}\quad - \int _I\int _\Omega f \cdot \partial _{t} y \varphi \textrm{d}x \textrm{d}t + \rho \int _I \int _\Omega \varphi \partial ^2_{tt} y \cdot \partial _t y \textrm{d}x \textrm{d}t. \end{aligned} \end{aligned}$$

By the chain rule, the fundamental theorem of calculus, and $\varphi (T) = 0$ we get

2.31 $$\begin{aligned} \int _I \int _\Omega \varphi \partial ^2_{tt} y \cdot \partial _t y \textrm{d}x \textrm{d}t&= \frac{1}{2}\int _I \int _\Omega \frac{\textrm{d}}{\textrm{d}t} \big ( \varphi | \partial _t y|^2 \big )\textrm{d}x \textrm{d}t - \frac{1}{2}\int _I \int _\Omega \partial _t \varphi | \partial _t y |^2 \textrm{d}x \textrm{d}t \nonumber \\  &= - \frac{1}{2} \int _{\Omega } |y'_{0}|^{2} \varphi (0) - \frac{1}{2}\int _I \int _\Omega \partial _t \varphi | \partial _t y |^2 \textrm{d}x \textrm{d}t. \end{aligned}$$

Moreover, by integration by parts in $\Omega$ and since $\partial _{t} y = 0$ in $I \times \partial \Omega$ (recall that $y (t) \in \mathcal {Y}_{\textbf{id}}$), we have that

2.32 $$\begin{aligned}&\int _I\int _\Omega {D H}(\Delta y) \cdot \Delta (\partial _{t} y \varphi ) \textrm{d}x \textrm{d}t = \int _I \int _\Omega {D H}(\Delta y) : \big ( \varphi \partial _{t}\Delta y + 2 \partial _{t} \nabla y \nabla \varphi + \partial _{t} y \Delta \varphi \big ) \textrm{d}x \textrm{d}t \nonumber \\&\quad = \int _I \int _\Omega {D H}(\Delta y) : \partial _{t}\Delta y \varphi \textrm{d}x \textrm{d}t - \int _I \int _\Omega {D H}(\Delta y) : \partial _{t} y \Delta \varphi \textrm{d}x \textrm{d}t \nonumber \\&\quad - 2 \int _I\int _\Omega \nabla ({D H}(\Delta y)) : (\partial _{t} y \otimes \nabla \varphi ) \textrm{d}x \textrm{d}t . \end{aligned}$$

Eventually, by the chain rule and by integration by parts with $\varphi (T) = 0$ we get

2.33 $$\begin{aligned}&\int _I\int _\Omega \varphi \big ( \partial _{F} W^\textrm{el} (\nabla y) : \partial _{t} \nabla y + {D H}(\Delta y) \cdot \partial _{t} \Delta y \big ) \textrm{d}x \textrm{d}t \nonumber \\&\quad `= \int _I\int _\Omega \varphi \frac{\textrm{d}}{\textrm{d}t} \big ( W^\textrm{el} ( \nabla y) + H(\Delta y) \big ) \textrm{d}x \textrm{d}t \nonumber \\&\quad = - \int _{\Omega } \varphi (0) \big ( W^\textrm{el} ( \nabla y_{0}) + H(\Delta y_{0}) \big )\textrm{d}x - \int _I\int _\Omega \partial _{t} \varphi \big ( W^\textrm{el} ( \nabla y) + H(\Delta y) \big ) \textrm{d}x \textrm{d}t \,. \end{aligned}$$

Combining (2.29)–(2.33) we infer (2.27).

### Outline

The rest of the paper is structured as follows. In Section 3 we consider a time-delayed parabolic system for a time-delay $h >0$, whose existence is established by a minimizing movement scheme with time discretization $\tau >0$. In Section 4 we pass to the limit $h \rightarrow 0$ and prove existence of solutions to the regularized system, see Theorem 2.2. Eventually in Section 5 we pass to the limit $\varepsilon \rightarrow 0$ and show Theorem 2.5. Importantly, all relevant a priori estimates are established already on the level $\tau >0$ in Section 3 and immediately transfer to the limits $\tau \rightarrow 0$, $h\rightarrow 0$, and $\varepsilon \rightarrow 0$.

## Minimizing movements for time-delayed parabolic system

We fix a *regularization parameter* $\varepsilon > 0$ and a *time-delay* $h > 0$. For convenience, without further notice, we assume that $T / h \in \mathbb {N}$. In this section, we include the $\varepsilon$-dependent regularizing term in (2.17) and we suppose more regular initial conditions $y_0$, $y_0'$, denoted by $y_{0,\varepsilon }$, $y_{0,\varepsilon }'$. As done in [5], by replacing the acceleration term $\rho \partial _{tt}^2 y$ by a discrete difference $\rho \frac{\partial _t y - \partial _t y(\cdot - h)}{h}$, we turn the hyperbolic problem (2.17a) into a parabolic one. The main goal of this section is to prove the following existence result for the resulting problem, where for convenience we use the notation $I_h:=[-h,T]$.

### Theorem 3.1

(Weak solutions of the time-delayed regularized problem) Let $T, \, h, \, \varepsilon > 0$, $y_{0,\varepsilon } \in \mathcal {Y}^\textrm{reg}_{\textbf{id}}$, $y_{0,\varepsilon }' \in H^{ 3}(\Omega ; \mathbb {R}^{d}) \cap H^1_{0}(\Omega ; \mathbb {R}^d)$, and $\theta _0 \in L^2_+(\Omega )$. Then, there exist $y_h \in L^\infty ( I; \mathcal {Y}^\textrm{reg}_{\textbf{id}}) \cap H^1(I_h; H^3(\Omega ; \mathbb {R}^d))$ with $y_h(t) = y_{0,\varepsilon } + t y_{0,\varepsilon }'$ for all $t \in [-h, 0]$ and $\theta _h \in L^2(I; H^1_+(\Omega ))$ such that $w_h :=W^{\textrm{in}}(\nabla y_h, \theta _h) \in L^2(I; H^1(\Omega )) \cap H^1(I; (H^1(\Omega ))^*)$ with $w_h(0) = w_{0,\varepsilon } :=W^{\textrm{in}}(\nabla y_{0,\varepsilon }, \theta _0)$ and the following holds true: For all $z \in C^\infty ( I \times \overline{\Omega }; \mathbb {R}^d)$ satisfying $z = 0$ on $I \times \partial \Omega$ we have

3.1a $$\begin{aligned} \begin{aligned}&\int _I\int _\Omega \Big ( \partial _F W(\nabla y_h, \theta _h) + \partial _{\dot{F}} R(\nabla y_h, \partial _t \nabla y_h, \theta _h) \Big ) : \nabla z + DH(\Delta y_h) \cdot \Delta z \\&+ \varepsilon \partial _t \nabla \Delta {y}_h : \nabla \Delta z \textrm{d}x \textrm{d}t \\&\quad = \int _I\int _\Omega f \cdot z \textrm{d}x \textrm{d}t - \frac{\rho }{h} \int _I \int _\Omega (\partial _t y_h(t) - \partial _t y_h(t - h)) \cdot z \textrm{d}x \textrm{d}t, \end{aligned} \end{aligned}$$

and for all $\varphi \in C^\infty ( I \times \overline{\Omega })$ it holds that

3.1b $$\begin{aligned} \begin{aligned}&\int _I \int _\Omega \mathcal {K}(\nabla y_h, \theta _h) \nabla \theta _h \cdot \nabla \varphi -\left( \xi (\nabla y_h, \partial _t \nabla y_h, \theta _h) + \partial _F W^{\textrm{cpl}}(\nabla y_h, \theta _h) : \partial _t \nabla y_h \right) \varphi \textrm{d}x \textrm{d}t \\&\quad - \varepsilon \int _{0}^{T} \int _{\Omega } | \partial _{t} \nabla \Delta {y}_h |^{2} \varphi \textrm{d}x \textrm{d}t + \int _I \langle \partial _t w_h , \varphi \rangle \textrm{d}t = \kappa \int _I \int _{\partial \Omega } (\theta _\flat - \theta _h) \varphi \textrm{d}\mathcal {H}^{d-1} \textrm{d}t , \end{aligned} \end{aligned}$$

where $\langle \cdot , \cdot \rangle$ denotes the duality pairing between $H^1(\Omega )$ and $(H^1(\Omega ))^*$.

A similar notion of weak solutions has already been considered in [2, 37]. The main differences of the above equations (3.1a)–(3.1b) to, e.g., [2, (2.19)-(2.20)] is the presence of the additional *h*-dependent terms arising from the time-discretization of the acceleration as well as the regularizing terms depending on $\varepsilon$, which induce better regularity properties of the solutions. The solutions also depend on $\varepsilon$, which we however do not include in the notation for simplicity. The proof of existence follows along the lines of the reasoning from [2, Sections 3 and 4] and is based on a minimizing movement scheme. To keep the presentation concise, many proofs in this section will only be sketched, highlighting primarily the differences to the arguments in [2].

### Staggered minimizing movement scheme and its well-definedness

We introduce a *discrete time-step* $\tau \in (0,h)$ and without further notice we assume that $h/\tau \in \mathbb {N}$. This also implies $T/\tau \in \mathbb {N}$. If not stated otherwise, all constants encountered in this section are independent of $\tau$, *h*, and $\varepsilon$. Given any sequence $(a_k)_{ k \in \mathbb {Z}}$, we introduce the notation for discrete differences as

3.2 $$\begin{aligned} \delta _\tau a_k :=\frac{a_k - a_{k-1}}{\tau }, \qquad k \in \mathbb {Z}. \end{aligned}$$

Theorem 3.1 will be shown via a *staggered* minimizing movements scheme. Let $y_{0,\varepsilon } \in \mathcal {Y}^\textrm{reg}_{\textbf{id}}$, $y_{0,\varepsilon }' \in H^3(\Omega ; \mathbb {R}^d) \cap H^1_0(\Omega ;\mathbb {R}^d)$, and $\theta _0 \in L^2_+(\Omega )$. We first define the initial conditions of the scheme by

$$\begin{aligned} y_{\tau }^{(k)} :=y_{0,\varepsilon } + k \tau y_{0,\varepsilon }' \quad \text { for } k \in \{-h/\tau , \, \ldots , \, 0\} \quad \text { and } \quad \theta _{\tau }^{(0)} :=\theta _0. \end{aligned}$$

Note that the time-discrete deformation is also defined for negative times, which will allow us to prove that the solution $y_h$ in (3.1a)–(3.1b) satisfies $y_h(t) = y_{0,\varepsilon } + t y_{0,\varepsilon }'$ for all $t \in [-h, 0]$.

Now, suppose that for $k \in \{1, \ldots , T / \tau \}$ we have already constructed $(y_\tau ^{(0)}, \theta _\tau ^{(0)}), \ldots ,$ $(y_\tau ^{(k-1)},\theta _\tau ^{(k-1)})$. (The solutions also depend on *h* and $\varepsilon$, which we do not include in the notation for simplicity.) Let , and for shorthand we denote by $(\cdot , \cdot )_{2}$ the scalar product in $L^{2}(\Omega ; \mathbb {R}^{d})$. Recalling also (2.6), (2.7), and (2.9), the next deformation $y_\tau ^{(k)}$ is defined as a solution of the minimization problem

3.3 $$\begin{aligned} \begin{aligned} \min _{y \in \mathcal {Y}_{\textbf{id}}\cap H^{ 3} (\Omega ; \mathbb {R}^{d})}&\Bigg \{ \mathcal {M}(y) \ + \mathcal {W}^{\textrm{cpl}}\big (y, \theta _{\tau }^{(k-1)}) + \frac{1}{\tau } \mathcal {R}\big (y_{\tau }^{(k-1)}, y - y_{\tau }^{(k-1)}, \theta _{\tau }^{(k-1)}\big ) \\&\ \ + \frac{\varepsilon }{2\tau } \Vert \nabla \Delta y - \nabla \Delta y_{\tau }^{(k-1)} \Vert _{L^{2}(\Omega )}^{2} - ( f_\tau ^{(k)} , y )_2 \\&\quad + \frac{\rho \tau }{2 h} \Big \Vert \frac{y - y_{\tau }^{(k-1)} }{\tau } - \delta _\tau y^{(k - h/\tau )}_\tau \Big \Vert _{L^2(\Omega )}^2 \Bigg \}. \end{aligned} \end{aligned}$$

Supposing that $y_\tau ^{(k)}$ exists, we define $\theta _{\tau }^{(k)}$ as a solution to the minimization problem

3.4 $$\begin{aligned}&\min _{\theta \in H^1_+(\Omega )} \Bigg \{ \int _\Omega \int _0^\theta \frac{1}{\tau }\left( W^{\textrm{in}}(\nabla y_{\tau }^{(k)},s) - W^{\textrm{in}}(\nabla y_{\tau }^{(k-1)}, \theta _{\tau }^{(k-1)}) \right) \textrm{d}s \textrm{d}x \nonumber \\&\quad + \frac{1}{2} \int _\Omega \mathcal {K}(\nabla y_{\tau }^{(k-1)}, \theta _{\tau }^{(k-1)}) \nabla \theta \cdot \nabla \theta \textrm{d}x \nonumber \\&\quad -\int _\Omega \left( \partial _F W^{\textrm{cpl}}(\nabla y_{\tau }^{(k-1)}, \theta _{\tau }^{(k-1)}) : \delta _\tau \nabla y_{\tau }^{(k)} + \xi (\nabla y_{\tau }^{(k-1)}, \delta _\tau \nabla y_{\tau }^{(k)}, \theta _{\tau }^{(k-1)}) \right. \nonumber \\&\quad \left. + \varepsilon | \delta _{\tau } \nabla \Delta y_{\tau }^{(k)} |^{2} \wedge \tau ^{-1} \right) \theta \textrm{d}x \nonumber \\&\qquad + \frac{\kappa }{2} \int _{{\partial \Omega }} (\theta - \theta _{\flat , \tau }^{(k)})^2 \textrm{d}\mathcal {H}^{d-1} \Bigg \}, \end{aligned}$$

where .

The minimization problem (3.3) differs from the one used in [5] due to the presence of the additional $\varepsilon$-regularizing term and a different discretization of the acceleration term. In [5, Definition 3.3, Theorem 3.5], the termfor a generic $v \in L^2(0,h)$ is used. By replacing *v* with $\partial _t y(\cdot - h)$, one can then construct a $\tau$-discretized solution in the small time interval (0, *h*). Then, a successive repetition of the argument yields a time-discrete solution on [0, *T*]. Here, instead, we simply use the discretized solution $\delta _\tau y^{(k - h/\tau )}_\tau$ which directly allows us to construct time-discrete solutions in the entire time horizon [0, *T*]. The minimization problem (3.4) coincides with the thermal step used in [2], except for the regularizing term $\varepsilon | \delta _{\tau } \nabla \Delta y_{\tau }^{(k)} |^{2} \wedge \tau ^{-1}$. In this context, the truncation by $\tau ^{-1}$ is necessary to guarantee well-posedness of the problem, see Proposition 3.4 below.

We now show the well-definedness of the above minimization problems. In this regard, the following properties of the mechanical energy are useful.

#### Lemma 3.2

(Coercivity of $\mathcal {M}$) Given $M > 0$ there exists a constant $C_M > 0$ such that for all $y \in \mathcal {Y}_{\textbf{id}}$ with $\mathcal {M}(y) \le M$ it holds that

3.5 $$\begin{aligned} \Vert y\Vert _{W^{2, p}(\Omega )}&\le C_M,&\Vert y\Vert _{C^{1, 1-d/p}(\Omega )}&\le C_M,&\Vert (\nabla y)^{-1}\Vert _{C^{1 - d/p}(\Omega )}&\le C_M,&\det (\nabla y) \ge \frac{1}{ C_M} \text { in } \Omega . \end{aligned}$$

#### Proof

By the definition of $\mathcal {H}$, the first inequality in (H.3) and (W.3) we see that

$$\begin{aligned} - C_0 |\Omega | + \int _\Omega |\Delta y|^p \textrm{d}x \le \mathcal {M}(y) \le M, \end{aligned}$$

and hence

$$\begin{aligned} \Vert \Delta y\Vert _{L^p(\Omega )}^p \le M + C_0 |\Omega | =:\tilde{M}. \end{aligned}$$

As $y - \textbf{id}\in W^{2, p}(\Omega ; \mathbb {R}^d) \cap W^{1, p}_0(\Omega ; \mathbb {R}^d)$ by the definition of $\mathcal {Y}_{\textbf{id}}$, we then derive from [32, Lemma 9.17] and the regularity of $\partial \Omega$ that

$$\begin{aligned} \Vert y - \textbf{id}\Vert _{W^{2, p}(\Omega )} \le C \Vert \Delta y\Vert _{L^p(\Omega )} \le C \tilde{M}^{1/p} \end{aligned}$$

for a constant *C* independent of *M*. Our choice of the elastic potential $W^{\textrm{el}}$ satisfies all assumptions imposed in [37]. Consequently, [37, Theorem 3.1] applies which directly leads to (3.5). $\square$

#### Proposition 3.3

(Existence of the mechanical step)

For any $M > 0$ there exists $\tau _0 \in (0, 1]$ such that for all $\tau \in (0, \tau _0)$ and $k \in \{1, \ldots , T/\tau \}$ the following holds: Let $y_{\tau }^{(k-1)} \in \mathcal {Y}_{\textbf{id}}\cap H^3(\Omega ; \mathbb {R}^d)$ satisfy $\mathcal {M}(y_{\tau }^{(k-1)}) \le M$ and let $\theta _{\tau }^{(k-1)} \in H^1_+(\Omega )$. Then, the minimization problem (3.3) admits a solution $y_{\tau }^{(k)} \in \mathcal {Y}_{\textbf{id}}\cap H^{3}(\Omega ; \mathbb {R}^{d})$ solving the corresponding Euler-Lagrange equation, i.e., for all $z \in H^3(\Omega ; \mathbb {R}^d) \cap H^1_0(\Omega ;\mathbb {R}^d)$ it holds that

3.6 $$\begin{aligned}&\int _\Omega \left( \partial _F W(\nabla y_{\tau }^{(k)}, \theta _{\tau }^{(k-1)}) + \partial _{\dot{F}} R(\nabla y_{\tau }^{(k-1)}, \delta _\tau \nabla y_{\tau }^{(k)}, \theta _{\tau }^{(k-1)}) \right) : \nabla z \nonumber \\&+ D H (\Delta y_{\tau }^{(k)}) \cdot \Delta z + \varepsilon \delta _\tau \nabla \Delta y^{(k)}_{\tau } : \nabla \Delta z \textrm{d}x \nonumber \\&\quad = \int _\Omega f_\tau ^{(k)} \cdot z \textrm{d}x - \frac{\rho }{h} \int _\Omega (\delta _\tau y_\tau ^{(k)} - \delta _\tau y_\tau ^{(k - h/\tau )}) \cdot z \textrm{d}x. \end{aligned}$$

Moreover, there exists a constant $$C_M >0 $$, possibly depending on *M*, such that

3.7 $$\begin{aligned} \begin{aligned}&\mathcal {M}(y_{\tau }^{(k)}) + \frac{\tau }{C_M} \Vert \delta _\tau \nabla y_{\tau }^{(k)}\Vert _{L^2(\Omega )}^2 + \frac{\varepsilon \tau }{2} \Vert \delta _\tau \nabla \Delta y_{\tau }^{(k)}\Vert _{L^{2}(\Omega )}^{2} \\&\le C_M( 1 + \Vert f \Vert _{L^2(I \times \Omega )}^2 ) + \frac{\rho \tau }{h} \Vert \delta _\tau y_{\tau }^{(k - h/\tau )} \Vert _{L^2(\Omega )}^2. \end{aligned} \end{aligned}$$

#### Proof

The proof is similar to the one in [2, Proposition 3.5]. We start by showing compactness. To this end, let $(y_n)_n \subset \mathcal {Y}_{\textbf{id}}\cap H^{ 3}(\Omega ; \mathbb {R}^d)$ be a minimizing sequence for the minimization problem in (3.3). Using $y_{\tau }^{(k-1)}$ as a competitor in (3.3), we may suppose that each $y_n$ satisfies

$$\begin{aligned}&\mathcal {M}(y_n) + \mathcal {W}^{\textrm{cpl}}(y_n, \theta _{\tau }^{(k-1)}) + \frac{1}{\tau } \mathcal {R}( y_{\tau }^{(k-1)}, y_n - y_{\tau }^{(k-1)}, \theta _{\tau }^{(k-1)}) + \frac{\varepsilon }{2\tau } \Vert \nabla \Delta y_{n} - \nabla \Delta y_{\tau }^{(k-1)}\Vert _{L^{2}(\Omega )}^{2} \\&\quad - ( f_\tau ^{(k)} , y_n )_2 + \frac{\rho \tau }{2 h} \Big \Vert \frac{y_n - y_{\tau }^{(k-1)}}{\tau } - \delta _\tau y_{\tau }^{(k - h/\tau )} \Big \Vert _{L^2(\Omega )}^2 \\&\qquad \le \mathcal {M}(y_{\tau }^{(k-1)}) + \mathcal {W}^{\textrm{cpl}}(y_{\tau }^{(k-1)}, \theta _{\tau }^{(k-1)}) - ( f_\tau ^{(k)} , y_{\tau }^{(k-1)} )_2 + \frac{\rho \tau }{2 h} \Vert \delta _\tau y_{\tau }^{(k - h/\tau )} \Vert _{L^2(\Omega )}^2. \end{aligned}$$

As the mechanical energy $\mathcal {M}$ satisfies the same coercivity properties as the one in [2], see Lemma 3.2, we can apply the generalized Korn’s inequality in the form [37, Corollary 3.4] (see also [38] for its original formulation). Consequently, reasoning similarly to the proof of [2, Proposition 3.5] for the terms involving $\mathcal {M}$, $\mathcal {W}^{\textrm{cpl}}$, $\mathcal {R}$, and $f_\tau ^{(k)}$, see [2, Equation (3.9)], we can find $C_M > 0$ and $\tau _0 \in (0, 1)$, possibly depending on *M*, such that for $\tau \in (0,\tau _0)$

$$\begin{aligned}&(1 - C_M \tau ) \mathcal {M}(y_n) + \frac{1}{C_M \tau } \Vert \nabla y_n - \nabla y_{\tau }^{(k-1)} \Vert _{L^2(\Omega )}^2 + \frac{\varepsilon }{2\tau } \Vert \nabla \Delta y_{n} - \nabla \Delta y_{\tau }^{(k-1)} \Vert _{L^{2}(\Omega )}^{2} \\&\quad + \frac{\rho \tau }{2 h} \Big \Vert \frac{y_n - y_{\tau }^{(k-1)}}{\tau } - \delta _\tau y_{\tau }^{(k - h/\tau )} \Big \Vert _{L^2(\Omega )}^2 \\&\qquad \le (1 + C_M \tau ) \mathcal {M}(y_{\tau }^{(k-1)}) + C_M \tau \big ( 1 + \Vert f_\tau ^{(k)} \Vert _{L^2(\Omega )}^2 \big ) + \frac{\rho \tau }{2h} \Vert \delta _\tau y_{\tau }^{(k - h/\tau )} \Vert _{L^2(\Omega )}^2. \end{aligned}$$

By the definition of $f_{\tau }^{(k)}$ and Jensen’s inequality we see thatThen, choosing $\tau _0$ small enough such that $C_M \tau _0 \le \frac{1}{2}$ and using $\mathcal {M}(y_{\tau }^{(k-1)}) \le M$, we see that, after possibly increasing $C_M$, it follows

3.8 $$\begin{aligned}&\mathcal {M}(y_n) + \frac{1}{C_M \tau } \Vert \nabla y_n- \nabla y_{\tau }^{(k-1)} \Vert _{L^2(\Omega )}^2 + \frac{\varepsilon }{2\tau } \Vert \nabla \Delta y_{n} - \nabla \Delta y_{\tau }^{(k-1)} \Vert _{L^{2}(\Omega )}^{2} \nonumber \\&\quad + \frac{\rho \tau }{2 h} \Big \Vert \frac{y_n - y_{\tau }^{(k-1)}}{\tau } - \delta _\tau y_{\tau }^{(k - h/\tau )} \Big \Vert _{L^2(\Omega )}^2 \nonumber \\&\qquad \le C_M (1 + \Vert f \Vert _{L^2(I \times \Omega )}^2 ) + \frac{\rho \tau }{h} \Vert \delta _\tau y_{\tau }^{(k - h/\tau )} \Vert _{L^2(\Omega )}^2. \end{aligned}$$

By Young’s inequality with power 2 and constant $\lambda \in (0, 1)$ we derive that

$$\begin{aligned} \Vert \nabla \Delta y_{n} - \nabla \Delta y_{\tau }^{(k)} \Vert _{L^{2}(\Omega )}^{2}&= \Vert \nabla \Delta y_{n}\Vert _{L^{2}(\Omega )}^{2} - 2 \int _\Omega \nabla \Delta y_{n} : \nabla \Delta y_{\tau }^{(k-1)} \textrm{d}x + \Vert \nabla \Delta y_{\tau }^{(k-1)}\Vert _{L^{2}(\Omega )}^{2} \\&\ge (1 - \lambda ) \Vert \nabla \Delta y_{n}\Vert _{L^{2}(\Omega )}^{2} - (-1 + 1 / \lambda ) \Vert \nabla \Delta y_{\tau }^{(k-1)}\Vert _{L^{2}(\Omega )}^{2}. \end{aligned}$$

Choosing $\lambda = 1/2$ above and combining with (3.8) this leads to

$$\begin{aligned}  &   \mathcal {M}(y_n) + \frac{\varepsilon }{4\tau } \Vert \Delta \nabla y_{n}\Vert _{L^{2}(\Omega )}^{2} \le + C_M (1 + \Vert f \Vert _{L^2(I \times \Omega )}^2) \\  &   + \frac{\varepsilon }{2\tau } \Vert \nabla \Delta y_{\tau }^{(k-1)}\Vert _{L^{2}(\Omega )}^{2} + \frac{\rho \tau }{h} \Vert \delta _\tau y_{\tau }^{(k - h/\tau )} \Vert _{L^2(\Omega )}^2. \end{aligned}$$

This implies $\sup _{n \in \mathbb {N}}\Vert \nabla \Delta y_n\Vert _{L^2(\Omega )} < \infty$ and, in view of (H.3), in particular shows $\sup _{n \in \mathbb {N}} \Vert \Delta y_n \Vert _{H^1(\Omega )} < \infty$. As $\Omega$ was assumed to have a $C^5$-boundary and $(y_n)_n \subset \mathcal {Y}_{\textbf{id}}$, elliptic regularity for the operator $\Delta$ implies

$$\begin{aligned} \Vert y_n - \textbf{id}\Vert _{H^3(\Omega )} \le C \Vert \Delta y_n \Vert _{H^1(\Omega )} , \end{aligned}$$

and thus $\sup _{n \in \mathbb {N}} \Vert y_n \Vert _{H^3(\Omega )} < \infty$. Consequently, as $p <2^*$, we can select a subsequence (without relabeling) such that $y_n \rightarrow y$ strongly in $W^{2, p}(\Omega ; \mathbb {R}^d)$ as well as $y_n \rightharpoonup y$ weakly in $H^3(\Omega ; \mathbb {R}^d)$.

Existence of minimizers then follows by standard lower semicontinuity arguments. Also, recalling (3.2), the derivation of the Euler-Lagrange equation (3.6) is standard, see the proof of [2, Proposition 3.5] for some details. Eventually, estimate (3.7) directly follows from (3.8), after passing to the limit $n \rightarrow \infty$ and using standard lower semicontinuity arguments. $\square$

#### Proposition 3.4

(Existence of the thermal step) For any $M > 0$ there exists $\tau _0 \in (0, 1]$ such that for all $\tau \in (0, \tau _0)$ and $k \in \{1, \ldots , T/\tau \}$ the following holds: Let $y_{\tau }^{(k-1)}, \, y_{\tau }^{(k)} \in \mathcal {Y}_{\textbf{id}}$ be such that $\mathcal {M}(y_{\tau }^{(k-1)}) \le M$ and let $\theta _{\tau }^{(k-1)} \in H^1_+(\Omega )$. Then, the minimization problem (3.4) admits a solution $\theta _\tau ^{(k)} \in H^1_+(\Omega )$ solving the corresponding Euler-Lagrange equation, i.e., for all $\varphi \in H^1(\Omega )$ it holds that

3.9 $$\begin{aligned} 0 =&\int _\Omega \delta _\tau w_{\tau }^{(k)} \varphi \textrm{d}x + \int _\Omega \mathcal {K}(\nabla y_{\tau }^{(k-1)}, \theta _{\tau }^{(k-1)}) \nabla \theta _{\tau }^{(k)} \cdot \nabla \varphi \textrm{d}x + \kappa \int _{\partial \Omega } (\theta _{\tau }^{(k)} - \theta _{\flat , \tau }^{(k)}) \varphi \textrm{d}\mathcal {H}^{d-1} \nonumber \\&- \int _\Omega \big ( \partial _F W^{\textrm{cpl}}(\nabla y_{\tau }^{(k-1)}, \theta _{\tau }^{(k-1)}) : \delta _\tau \nabla y_{\tau }^{(k)} + \xi (\nabla y_{\tau }^{(k-1)}, \delta _\tau \nabla y_{\tau }^{(k)}, \theta _{\tau }^{(k-1)}) \nonumber \\&+ \varepsilon |\delta _{\tau } \nabla \Delta y^{(k)}_{\tau } |^{2} \wedge \tau ^{-1} \big ) \varphi \textrm{d}x\,, \end{aligned}$$

where we shortly write $w_{\tau }^{(k-1)} :=W^{\textrm{in}}(\nabla y_{\tau }^{(k-1)}, \theta _{\tau }^{(k-1)})$, $w_{\tau }^{(k)} :=W^{\textrm{in}}(\nabla y_{\tau }^{(k)}, \theta _{\tau }^{(k)})$, and $\delta _\tau w_{\tau }^{(k)} :=\frac{w_{\tau }^{(k)} - w_{\tau }^{(k-1)}}{\tau }$.

#### Proof

The thermal step differs from the one used in [2] only by the regularization of the dissipation term $\xi (\nabla y_{\tau }^{(k-1)}, \delta _\tau \nabla y_{\tau }^{(k)}, \theta _{\tau }^{(k-1)})$ by $\varepsilon |\delta _{\tau } \nabla \Delta y^{(k)}_{\tau } |^{2} \wedge \tau ^{-1}$. Therefore, the statement immediately follows from [2, Proposition 3.8] since an inspection of its proof shows that for the existence of $\theta _\tau ^{(k)}$ it is enough that the dissipation term lies in $L^\infty (\Omega )$ (see Steps 1 and 2) and for nonnegativity of $\theta _\tau ^{(k)}$ it is enough that the dissipation is larger than $c|(\delta _\tau \nabla y_{\tau }^{(k)})^T\nabla y_{\tau }^{(k-1)} + (\nabla y_{\tau }^{(k-1)})^T \delta _\tau \nabla y_{\tau }^{(k)}|^2$, see [2, Remark 3.9] and (D.1). $\square$

### Existence of time-discrete solutions

In the previous subsection, we focused on one step in the staggered scheme. Our next goal is to prove the existence of time-discrete solutions and to derive first a priori bounds independent of $\tau$, *h*, and $\varepsilon$. We set

3.10 $$\begin{aligned} C_{f} :=\Vert f\Vert _{W^{1,1}(I; L^2(\Omega ))}, \end{aligned}$$

and note that by the fundamental theorem of calculus it holds that

3.11 $$\begin{aligned} \Vert f(t) \Vert _{L^2(\Omega )} \le C_T C_{f} \quad \text {for all }t \in I, \end{aligned}$$

where here and in the following $C_T$ denotes a constant possibly depending on *T*.

Given the sequences $y_{\tau }^{(0)}, \, \ldots ,\, y_{\tau }^{(k)}$ and $\theta _{\tau }^{(0)}, \, \ldots , \, \theta _{\tau }^{(k)}$ for some $k \in \{1, \, \ldots , \, T/\tau \}$, as described in Subsection 3.1, for $l \in \{0, \, \ldots , \, k\}$ we define

$$\begin{aligned} \mathcal {F}^{(l)} :=\mathcal {E}(y_{\tau }^{(l)}, \theta _{\tau }^{(l)}) - ( f(l \tau ), y_{\tau }^{(l)} )_2, \end{aligned}$$

where the total internal energy $\mathcal {E}$ is defined in (2.16). Then, using (W.3) we find

3.12 $$\begin{aligned} |( f(l \tau ), y_{\tau }^{(l)} )_2| \le \min \{ \mathcal {F}^{(l)}, \mathcal {E}(y_{\tau }^{(l)}, \theta _{\tau }^{(l)}) \} + C_T C_{f}^2 + C(1+C_0), \end{aligned}$$

see also [2, Lemma 3.10]. Finally, for $l \in \{ 0, \, \ldots , \, k \}$ we also define

3.13 $$\begin{aligned} \mathcal {G}^{(l)} :=\mathcal {F}^{(l)} + \frac{\rho }{2} \frac{\tau }{h} \sum _{m = l-h/\tau +1}^l \Vert \delta _\tau y_{\tau }^{(m)} \Vert _{L^2(\Omega )}^2 , \end{aligned}$$

which corresponds to adding also a suitably averaged kinetic energy. The main result of this subsection reads as follows.

#### Proposition 3.5

(Existence of time-discrete solutions) Let $$ C_f$$ be as in (3.10) and $$\mathcal {G}^{(0)}$$ be as in (3.13). For any $$T > 0$$, there exist a constant $${\overline{C}}_T >0 $$, corresponding constants

$$\begin{aligned} M' :=2 e^{{\overline{C}}_T C_{f}} \Big ( \mathcal {G}^{(0)} + {\overline{C}}_T (1 + C_{f}^3) + \kappa \int _I \int _{\partial \Omega } \theta _\flat \textrm{d}\mathcal {H}^{d-1} \textrm{d}t \Big ), \qquad M :=2M' + {\overline{C}}_T C_{f}^2, \end{aligned}$$

as well as a constant $$C_M >0 $$ and scalar $$\tau _0 \in (0, h]$$ only depending on *M* above such that the following holds true: For each $$\tau \in (0, \tau _0)$$ such that $$h / \tau \in \mathbb {N}$$ the sequences $$y_{\tau }^{(0)}, \ldots , y_{\tau }^{(T / \tau )}$$ and $$\theta _{\tau }^{(0)}, \ldots , \theta _{\tau }^{(T / \tau )}$$ constructed in Subsection 3.1 exist, and for all $$k \in \{ 0, \ldots , T / \tau \}$$ it holds that

3.14 $$\begin{aligned} \mathcal {E}(y_{\tau }^{(k)}, \theta _{\tau }^{(k)}) + \frac{\rho }{2} \frac{\tau }{h} \sum _{l = k - h/\tau + 1}^k \Vert \delta _\tau y_{\tau }^{(l)} \Vert _{L^2(\Omega )}^2&\le M, \end{aligned}$$

3.15 $$\begin{aligned} \sum _{l = 1}^{k} \tau \Big ( \Vert \delta _\tau \nabla y_{\tau }^{(l)} \Vert _{L^2(\Omega )}^2 + \varepsilon \Vert \delta _{\tau } \nabla \Delta y^{(l)}_{\tau } \Vert _{L^{2}(\Omega )}^{2} \Big )&\le C_M (M (1+T) + {\overline{C}}_T C_{f}^2). \end{aligned}$$

The first estimate corresponds to a bound on the total energy and the second one is a bound on the (regularized) strain rate. The proof relies on the following two lemmas.

#### Lemma 3.6

(Inductive bound on the total energy) For any $M,\, T > 0$ there exist constants $C_M >0$ and $C_T >0$ only depending on *M* and *T*, respectively, such that the following holds true: Suppose $\tau \in (0, 1)$ is chosen such that for $k \in \{1, \, \ldots , \, T / \tau \}$ the sequences $y_{\tau }^{(0)}, \, \ldots , \, y_{\tau }^{(k)}$ and $\theta _{\tau }^{(0)}, \, \ldots , \, \theta _{\tau }^{(k)}$ constructed in Subsection 3.1 exist. Moreover, assume that $\mathcal {G}^{(l)} \le M$ for all $l \in \{ 0, \, \ldots , \, k-1 \}$ with $\mathcal {G}^{(l)}$ as in (3.13). Then, it holds that

$$\begin{aligned} \begin{aligned} \mathcal {G}^{(k)}&\le \mathcal {G}^{(0)} + C_M \tau V_k + C_T(1 + C_{f}^3) + \kappa \int _0^{k \tau } \int _{\partial \Omega } \theta _\flat \textrm{d}\mathcal {H}^{d-1} \textrm{d}t \\&+ C\sum _{l = 0}^k \mathcal {G}^{(l)} \int _{(l-1)\tau }^{(l+1)\tau } \Vert \partial _t f(t) \Vert _{L^2 (\Omega )} \textrm{d}t, \end{aligned} \end{aligned}$$

where $C >0$ is a universal constant, and

3.16 $$\begin{aligned} V_k :=\sum _{m = 1}^k \tau \int _\Omega |\delta _\tau \nabla y_{\tau }^{(m)}|^2 \textrm{d}x. \end{aligned}$$

#### Lemma 3.7

(Inductive bound on the strain rates) Given $M, \, T > 0$, there exist a constant $C_M >0$ and $\tau _0 \in (0, 1]$ only depending on *M*, and a constant $C_T >0$ only depending on *T* such that for $\tau \in (0, \tau _0)$ the following holds: Suppose that the sequences $y_{\tau }^{(0)}, \, \ldots , \, y_{\tau }^{(k)}$ and $\theta _{\tau }^{(0)}, \, \ldots , \, \theta _{\tau }^{(k)}$ for some $k \in \{1, \ldots , T / \tau \}$ constructed in Subsection 3.1 exist. Moreover, suppose that $\mathcal {G}^{(l)} \le M$ for all $l \in \{0, \, \ldots , \, k-1\}$ with $\mathcal {G}^{(l)}$ as defined in (3.13). Then,

3.17 $$\begin{aligned} \begin{aligned}&\sum _{l = 1}^{k} \tau \Big ( \Vert \delta _\tau \nabla y_{\tau }^{(l)} \Vert _{L^2(\Omega )}^2 + \varepsilon \Vert \delta _{\tau } \nabla \Delta y^{(l)}_{\tau } \Vert _{L^{2}(\Omega )}^{2} \Big )\\&\quad \le C_M \left( \mathcal {M}(y_{0,\varepsilon }) + \frac{\rho }{2} \Vert y_{0,\varepsilon }' \Vert _{L^2(\Omega )}^2 + C_T C_{f}^2 \right) + C_M \tau \sum _{l = 0}^{k-1} \big (1+ \mathcal {M}(y_{\tau }^{(l)})\big ) . \end{aligned} \end{aligned}$$

#### Proof of Proposition 3.5

Once Lemmas 3.6–3.7 are proved, the proof of Proposition 3.5 follows by an inductive argument using the discrete Gronwall’s inequality and Propositions 3.3–3.4. We refer to [2, Theorem 3.13] for all details (cf. also [2, Lemma 3.11, Lemma 3.12]). $\square$

We now proceed with the proof of the lemmas. As an auxiliary result, we show $\Lambda$-convexity of $W^{\textrm{el}}$ and $W^{\textrm{cpl}}$. The result is closely related to the estimate in [15, Subsection 2.3], which improved upon [37, Proposition 3.2], where *local* $\Lambda$-convexity has been shown.

#### Lemma 3.8

($\Lambda$-convexity of $W^{\textrm{el}}$ and $W^{\textrm{cpl}}$) For any $M > 0$ there exists a constant $C_M>0$ such that for all $y_1, y_2 \in \mathcal {Y}_{\textbf{id}}$ with $\mathcal {M}(y_1), \mathcal {M}(y_2) \le M$ and $\theta \in {\L } ^1(\Omega )$, we have

$$\begin{aligned}&\int _\Omega W^{\textrm{el}}(y_{2}) \, \textrm{d}x \ge \int _\Omega W^{\textrm{el}}(y_1) \textrm{d}x + \int _\Omega \partial _F W^{\textrm{el}}(\nabla y_1) : \big ( \nabla y_2 - \nabla y_1 \big ) \textrm{d}x \\&\quad - C_M \Vert \nabla y_2 - \nabla y_1 \Vert ^2_{L^2(\Omega )}. \\&\int _\Omega W^{\textrm{cpl}}(y_{2},\theta ) \, \textrm{d}x \ge \int _\Omega W^{\textrm{cpl}}(y_1,\theta ) \textrm{d}x + \int _\Omega \partial _F W^{\textrm{cpl}}(\nabla y_1,\theta ) : \\&\quad \big ( \nabla y_2 - \nabla y_1 \big ) \textrm{d}x - C_M \Vert \nabla y_2 - \nabla y_1 \Vert ^2_{L^2(\Omega )}. \end{aligned}$$

#### Proof

We first prove the statement for $W^{\textrm{el}}$. By Lemma 3.2 there exists a constant $C^*_M$ depending on *M* such that for all $x \in \Omega$ and $l \in \{ 1,2\}$ it holds that

3.18 $$\begin{aligned} |\nabla y_l(x)| \le C_M^*, \quad \det (\nabla y_l(x)) \ge \frac{1}{C_M^*}. \end{aligned}$$

By the continuity of the determinant we can find $\delta _M > 0$ such that for all $\lambda \in (0, 1)$ and $F_1, \, F_2 \in GL^+(d)$ with $|F_1|, |F_2| \le C_M^*$, $\det (F_1), \, \det (F_2) \ge \frac{1}{C_M^*}$ and $|F_1 - F_2| \le \delta _M$ we have that $\det (\lambda F_1 + (1 - \lambda ) F_2) \ge \frac{1}{2 C_M^*}$. The set

$$\begin{aligned} K :=\left\{ F \in GL^+(d) :|F| \le C_M^*, \, \det (F) \ge \frac{1}{2 C_M^*}\right\} \end{aligned}$$

is a compact subset of $GL^+(d)$. Hence, by the $C^2$-regularity of $W^{\textrm{el}}$ it follows that

$$\begin{aligned} C_M :=\sup _{F \in K} | \partial _{FF}^2 W^{\textrm{el}}(F)| < \infty . \end{aligned}$$

With $\delta _M$ as above, let us define the set $G :=\{x \in \Omega :|\nabla y_2(x) - \nabla y_{1}(x)| \le \delta _M \}$. Moreover, we define $y_t = (1-t)y_1 + ty_2$ for $t \in [0,1]$. By the previous reasoning, for every $x \in G$ it holds that $\nabla y_t(x) \in K$ and

3.19 $$\begin{aligned} \begin{aligned}&|\partial _F W^{\textrm{el}}(\nabla y_t(x)) - \partial _F W^{\textrm{el}}(\nabla y_1(x))| \le C_M |\nabla y_t(x) - \nabla y_1(x)| \le C_M |\nabla y_2(x) - \nabla y_1(x)|. \end{aligned} \end{aligned}$$

On the one hand, by the Gateaux differentiability of $\mathcal {W}^\textrm{el}$ (see [37, Proposition 3.2]) and the chain rule we have that

$$\begin{aligned} \int _G W^{\textrm{el}}(\nabla y_2) \, \textrm{d}x- \int _G W^{\textrm{el}}(\nabla y_1) \, \textrm{d}x&= \int _0^1 \int _G \partial _FW^{\textrm{el}}(\nabla y_t) : \big ( \nabla y_2 - \nabla y_1\big ) \, \textrm{d}x \textrm{d}t . \end{aligned}$$

On the other hand, using (3.19) we can estimate

$$\begin{aligned}&\Bigg | \int _0^1 \int _G \partial _FW^{\textrm{el}}(\nabla y_t) : \big ( \nabla y_2 - \nabla y_1\big ) \, \textrm{d}x \textrm{d}t - \int _0^1 \int _G \partial _FW^{\textrm{el}}(\nabla y_1) : \big ( \nabla y_2 - \nabla y_1\big ) \textrm{d}x \textrm{d}t \Bigg | \\&\le C_M \Vert \nabla y_2 - \nabla y_1\Vert _{L^2(\Omega )}^2. \end{aligned}$$

By the definition of *G* we also see that, by possibly increasing $C_M$, it holds

3.20 $$\begin{aligned}&\Big |\int _{\Omega \setminus G} W^{\textrm{el}}(\nabla y_2) \, \textrm{d}x - \int _{\Omega \setminus G} W^{\textrm{el}}(\nabla y_1) \textrm{d}x - \int _{\Omega \setminus G} \partial _F W^{\textrm{el}}(\nabla y_1) : \big ( \nabla y_2 - \nabla y_1 \big ) \textrm{d}x\Big | \nonumber \\&\quad \le \int _{\Omega \setminus G} \frac{|W^{\textrm{el}}(\nabla y_1)| + |W^{\textrm{el}}(\nabla y_2)| + \delta _M|\partial _F W^{\textrm{el}}(\nabla y_1)| }{\delta _M^2} |\nabla y_2 - \nabla y_1|^2 \textrm{d}x \nonumber \\&\quad \le C_M \Vert \nabla y_2 - \nabla y_1 \Vert _{L^2(\Omega )}^2, \end{aligned}$$

where we used that $W^{\textrm{el}}(\nabla y_1)$, $W^{\textrm{el}}(\nabla y_2)$, and $|\partial _F W^{\textrm{el}}(\nabla y_1)|$ are uniformly bounded by (3.18). The combination of the last three estimates gives the statement for $W^{\textrm{el}}$.

The argument for $W^{\textrm{cpl}}$ is similar. However, the bounds in (3.19) and (3.20) do not follow immediately from the compactness of *K* due to the presence of the temperature $\theta \in L^1(\Omega )$. To obtain the analogous bounds, we use (C.4), the first inequality in (C.5), and (3.18). $\square$

As a second auxiliary result, we establish a bound on the mechanical energy.

#### Lemma 3.9

(Mechanical energy bound in the time-discrete setting)

Given $M > 0$, there exists a constant $C_M>0$ such that the following holds: Suppose that for $\tau \in (0,\tau _0)$ and $k \in \{1, \, \ldots , \, T / \tau \}$ the sequences $y_{\tau }^{(0)}, \, \ldots , \, y_{\tau }^{(k)}$ and $\theta _{\tau }^{(0)}, \, \ldots , \, \theta _{\tau }^{(k)}$ constructed in Subsection 3.1 exist, and that $\mathcal {G}^{(l)}\le M$ for all $l \in \{0, \, \ldots , \, k-1\}$. Then, it holds that

$$\begin{aligned}&\mathcal {M}(y_{\tau }^{(k)}) + \frac{\rho \tau }{2h} \sum _{l=k - h/\tau + 1}^k \Vert \delta _\tau y_{\tau }^{(l)} \Vert _{L^2(\Omega )}^2 + \sum _{l = 1}^k \tau \Big ( 2\mathcal {R}_\varepsilon ( y_{\tau }^{(l-1)}, \delta _\tau y_{\tau }^{(l)}, \theta _{\tau }^{(l-1)}) \\&+ \frac{\rho }{2h} \Vert \delta _\tau y_{\tau }^{(l)} - \delta _\tau y_{\tau }^{(l - h/\tau )} \Vert _{L^2(\Omega )}^2 \Big ) \\  &\le \mathcal {M}(y_{0,\varepsilon }) + \frac{\rho }{2} \Vert y_{0,\varepsilon }' \Vert _{L^2(\Omega )}^2 - \tau \sum _{l=1}^k \int _\Omega \partial _F W^{\textrm{cpl}}(\nabla y_{\tau }^{(l-1)}, \theta _{\tau }^{(l-1)}) : \delta _\tau \nabla y_{\tau }^{(l)} \textrm{d}x \\&+ \tau \sum _{l = 1}^k ( f_{\tau }^{(l)}, \delta _\tau y_{\tau }^{(l)} )_2 + C_M \tau V_k, \end{aligned}$$

where $\mathcal {R}_\varepsilon$ is given in (2.12) and $V_k$ in (3.16).

#### Proof

Using Proposition 3.3 for *l* in place of *k*, (2.11), and testing (3.6) with $z = \delta _\tau y_{\tau }^{(l)}$ it follows that

3.21 $$\begin{aligned} 0 =&\int _\Omega \partial _F W(\nabla y_{\tau }^{(l)}, \theta _{\tau }^{(l-1)}) : \delta _\tau \nabla y_{\tau }^{(l)} + D H (\Delta y_{\tau }^{(l)}) \cdot \delta _\tau \Delta y_{\tau }^{(l)} + \varepsilon \delta _{\tau } \nabla \Delta y^{(l)}_{\tau } : \delta _{\tau } \nabla \Delta y^{(l)}_{\tau } \textrm{d}x \nonumber \\&+ \int _\Omega \xi (\nabla y_{\tau }^{(l-1)}, \delta _\tau \nabla y_{\tau }^{(l)}, \theta _{\tau }^{(l-1)}) \textrm{d}x - \int _\Omega f_{\tau }^{(l)} \cdot \delta _\tau y_{\tau }^{(l)} \textrm{d}x \nonumber \\&+ \frac{\rho }{h} \int _\Omega \big (\delta _\tau y_{\tau }^{(l)} - \delta _\tau y_{\tau }^{(l-h/\tau )} \big ) \cdot \delta _\tau y_{\tau }^{(l)} \textrm{d}x. \end{aligned}$$

By the convexity of $H$ (see (H.1)) it follows for $l \in \{ 1, \ldots , k \}$ that

3.22 $$\begin{aligned} \mathcal {H}(\Delta y_{\tau }^{(l-1)})&\ge \mathcal {H}(\Delta y_{\tau }^{(l)}) + \int _\Omega D H(\Delta y_{\tau }^{(l)}) \cdot (\Delta y_{\tau }^{(l-1)} - \Delta y_{\tau }^{(l)}) \, \textrm{d}x = \mathcal {H}(\Delta y_{\tau }^{(l)}) \nonumber \\&- \tau \int _\Omega D H (\Delta y_{\tau }^{(l)}) \cdot \delta _\tau \Delta y_{\tau }^{(l)} \textrm{d}x, \end{aligned}$$

where we recall the notation in (2.3). We now perform a similar argument for the elastic energy. Using (3.10), (3.12)–(3.13), and $\mathcal {G}^{(l)}\le M$ for $l \in \{ 0, \, \ldots , \, k - 1 \}$, we get

3.23 $$\begin{aligned} \mathcal {M}(y_{\tau }^{(l-1)}) \le \mathcal {G}^{(l-1)} + ( f((l-1) \tau ), y_{\tau }^{(l-1)} )_2 \le 2 \mathcal {G}^{(l-1)} + C + C_T C_{f}^2 \le 2 M + C + C_T C_{f}^2 \end{aligned}$$

for all $l \in \{ 1, \, \ldots , \, k \}$. Thus, we can apply Proposition 3.3 for all $l \in \{ 1, \, \ldots , \, k \}$, where now $C_M$ may also depend on *T* and *f*. Then, using again $\mathcal {G}^{(l)}\le M$ for $l \in \{ 0, \, \ldots , \, k - 1 \}$, by (3.7) we find for all $l \in \{ 1, \, \ldots , \, k \}$ that

3.24 $$\begin{aligned} \mathcal {M}(y_{\tau }^{(l)}) \le C_M \big ( 1 + \Vert f \Vert _{L^2(I \times \Omega )}^2 \big ) + 2\mathcal {G}^{(l-1)} \le 2 M + C_M(1 + C_T C_{f}^2). \end{aligned}$$

Consequently, by (3.2), (3.23)–(3.24), and Lemma 3.8 applied for $y_1 = y_{\tau }^{(l)}$ and $y_2 = y_{\tau }^{(l-1)}$ we find

3.25 $$\begin{aligned} \tau \int _\Omega \partial _F W^{\textrm{el}}(\nabla y_{\tau }^{(l)}) : \delta _\tau \nabla y_{\tau }^{(l)} \textrm{d}x \ge \mathcal {W}^{\textrm{el}}(y_{\tau }^{(l)}) - \mathcal {W}^{\textrm{el}}( y_{\tau }^{(l-1)}) - C_M \tau ^2 \int _\Omega |\delta _\tau \nabla y_{\tau }^{(l)}|^2 \textrm{d}x \end{aligned}$$

for a possibly larger $C_M$, where we recall the definition in (2.2). Multiplying (3.21) by $\tau$, using (3.22) and (3.25), and summing over $l \in \{ 1, \ldots , k \}$, we conclude

3.26 $$\begin{aligned}&\mathcal {M}(y_{\tau }^{(k)}) + \frac{\tau \rho }{h} \sum _{l=1}^k \int _\Omega \big ( \delta _\tau y_{\tau }^{(l)} - \delta _\tau y_{\tau }^{(l-h/\tau )} \big ) \cdot \delta _\tau y_{\tau }^{(l)} \textrm{d}x \nonumber \\&\quad + \sum _{l = 1}^k\tau \Big ( \int _\Omega \xi (\nabla y_{\tau }^{(l-1)}, \delta _\tau \nabla y_{\tau }^{(l)}, \theta _{\tau }^{(l-1)}) \textrm{d}x +\varepsilon \Vert \delta _{\tau } \nabla \Delta y^{(l)}_{\tau } \Vert ^2_{L^2(\Omega )} \Big ) \nonumber \\&\qquad \le \mathcal {M}(y_{0,\varepsilon }) - \tau \sum _{l=1}^k \int _\Omega \partial _F W^{\textrm{cpl}}(\nabla y_{\tau }^{(l)}, \theta _{\tau }^{(l-1)}) : \delta _\tau \nabla y_{\tau }^{(l)} \textrm{d}x \nonumber \\&\quad + \tau \sum _{l = 1}^k ( f_{\tau }^{(l)}, \delta _\tau y_{\tau }^{(l)} )_2 + C_M \tau V_k, \end{aligned}$$

where we employed the definitions in (2.6) and (3.16). Using the identity

$$\begin{aligned}  &   \Pi _l :=\int _\Omega \big ( \delta _\tau y_{\tau }^{(l)} - \delta _\tau y_{\tau }^{(l-h/\tau )} \big ) \cdot \delta _\tau y_{\tau }^{(l)} \textrm{d}x = \frac{1}{2} (\Vert \delta _\tau y_{\tau }^{(l)} \Vert _{L^2(\Omega )}^2 \\  &   - \Vert \delta _\tau y_{\tau }^{(l - h/\tau )} \Vert _{L^2(\Omega )}^2 + \Vert \delta _\tau y_{\tau }^{(l)} - \delta _\tau y_{\tau }^{(l - h/\tau )} \Vert _{L^2(\Omega )}^2), \end{aligned}$$

summing over $l \in \{ 1, \ldots , k \}$, and recalling the definition $y_{\tau }^{(m)} = y_{0,\varepsilon } + m \tau y_{0,\varepsilon }'$ for $m \in \{-h/\tau , \ldots , 0\}$ we derive that

3.27 $$\begin{aligned} \sum _{l=1}^k 2\tau \Pi _l&= \sum _{l=1}^k \tau \Vert \delta _\tau y_{\tau }^{(l)} - \delta _\tau y_{\tau }^{(l - h/\tau )} \Vert _{L^2(\Omega )}^2 + \sum _{l=k - h/\tau + 1}^k \tau \Vert \delta _\tau y_{\tau }^{(l)} \Vert _{L^2(\Omega )}^2 - \sum _{l=-h/\tau + 1}^0 \tau \Vert \delta _\tau y_{\tau }^{(l)} \Vert _{L^2(\Omega )}^2 \nonumber \\  &= \sum _{l=1}^k \tau \Vert \delta _\tau y_{\tau }^{(l)} - \delta _\tau y_{\tau }^{(l - h/\tau )} \Vert _{L^2(\Omega )}^2 + \sum _{l=k - h/\tau + 1}^k \tau \Vert \delta _\tau y_{\tau }^{(l)} \Vert _{L^2(\Omega )}^2 - h \Vert y_{0,\varepsilon }' \Vert _{L^2(\Omega )}^2. \end{aligned}$$

We apply Lemma 3.8 first for $y_1 = y_{\tau }^{(l-1)}$ and $y_2 = y_{\tau }^{(l)}$, then for $y_1 = y_{\tau }^{(l)}$ and $y_2 = y_{\tau }^{(l-1)}$, and sum the equations to get

$$\begin{aligned} 0 \ge \int _\Omega  &   \Big ( \partial _F W^{\textrm{cpl}}(\nabla y_{\tau }^{(l)}, \theta _{\tau }^{(l-1)}) - \partial _F W^{\textrm{cpl}}(\nabla y_{\tau }^{(l-1)}, \theta _{\tau }^{(l-1)}) \Big ) : \\  &   \big ( \nabla y_{\tau }^{(l-1)} - \nabla y_{\tau }^{(l)} \big ) \textrm{d}x - 2C_M \tau ^2 \int _\Omega |\delta _\tau \nabla y_{\tau }^{(l)}|^2 \textrm{d}x. \end{aligned}$$

Rearranging and summing over $l \in \lbrace 1,\ldots , k\rbrace$ yields

3.28 $$\begin{aligned} \tau \sum _{l = 1}^k \int _\Omega \Big ( \partial _F W^{\textrm{cpl}}(\nabla y_{\tau }^{(l)}, \theta _{\tau }^{(l-1)}) - \partial _F W^{\textrm{cpl}}(\nabla y_{\tau }^{(l-1)}, \theta _{\tau }^{(l-1)}) \Big ) : \delta _\tau \nabla y_{\tau }^{(l)} \textrm{d}x \ge - 2C_M \tau V_k. \end{aligned}$$

Eventually, recalling (2.11)–(2.12), the combination of (3.26), (3.27), and (3.28) concludes the proof. $\square$

We now proceed with the proofs of Lemma 3.6 and Lemma 3.7.

#### Proof of Lemma 3.6

Recalling the argument in (3.23), we observe that Proposition 3.4 is applicable by passing to a larger value of *M*. We test (3.9) (for *l* in place of *k*) with $\varphi = 1$ to obtain

$$\begin{aligned} 0 =&\int _\Omega \Big ( \delta _\tau w_\tau ^{(l)} -\partial _F W^{\textrm{cpl}}(\nabla y_{\tau }^{(l-1)}, \theta _{\tau }^{(l-1)}) : \delta _\tau \nabla y_{\tau }^{(l)} - \xi (\nabla y_{\tau }^{(l-1)}, \delta _\tau \nabla y_{\tau }^{(l)}, \theta _{\tau }^{(l-1)}) \textrm{d}x \\&- \varepsilon |\delta _{\tau } \nabla \Delta y^{(\tau )}_{k} |^{2} \wedge \tau ^{-1} \Big ) \textrm{d}x \\&\quad + \kappa \int _{\partial \Omega } (\theta _{\tau }^{(l)} - \theta _{\flat , \tau }^{(l)}) \textrm{d}\mathcal {H}^{d-1}. \end{aligned}$$

Multiplying this equation by $\tau$, summing over $l \in \{1, \, \ldots , \, k\}$, and adding to the estimate in Lemma 3.9, by (2.11)–(2.12) we discover that

$$\begin{aligned}&\mathcal {M}(y_{\tau }^{(k)}) + \frac{\rho \tau }{2h} \sum _{l=k - h/\tau + 1}^k \Vert \delta _\tau y_{\tau }^{(l)} \Vert _{L^2(\Omega )}^2 + \int _\Omega w^{(k)}_\tau \textrm{d}x \\  &\le \mathcal {M}(y_{0,\varepsilon }) + \int _\Omega w_{0,\varepsilon } \textrm{d}x + \frac{\rho }{2} \Vert y_{0,\varepsilon }' \Vert _{L^2(\Omega )}^2 \\&+ \tau \sum _{l = 1}^k ( f_{\tau }^{(l)}, \delta _\tau y_{\tau }^{(l)} )_2 + \tau \sum _{l = 1}^k \kappa \int _{\partial \Omega } (\theta _{\flat , \tau }^{(l)} - \theta _{\tau }^{(l)}) \textrm{d}\mathcal {H}^{d-1} + C_M \tau V_k, \end{aligned}$$

where $w_{0,\varepsilon } = W^{\textrm{in}}(\nabla y_{0,\varepsilon }, \theta _0)$. Recalling the definition of $\mathcal {E}$ in (2.16), we conclude that

3.29 $$\begin{aligned}&\mathcal {E}(y_{\tau }^{(k)}, \theta _{\tau }^{(k)}) + \frac{\rho \tau }{2h} \sum _{l=k - h/\tau + 1}^k \Vert \delta _\tau y_{\tau }^{(l)} \Vert _{L^2(\Omega )}^2 \nonumber \\&\le \mathcal {E}(y_{0,\varepsilon }, \theta _{0} ) + \frac{\rho }{2} \Vert y_{0,\varepsilon }' \Vert _{L^2(\Omega )}^2 + C_M \tau V_k + \tau \sum _{l = 1}^k ( f_{\tau }^{(l)}, \delta _\tau y_{\tau }^{(l)} )_2 \nonumber \\&\quad + \tau \sum _{l = 1}^k \kappa \int _{\partial \Omega } (\theta _{\flat , \tau }^{(l)} - \theta _{\tau }^{(l)}) \textrm{d}\mathcal {H}^{d-1} . \end{aligned}$$

Now, we estimate the last two terms on the right-hand side of (3.29). By the nonnegativity of $\theta _{\tau }^{(l)}$ and the definition of $\theta _{\flat , \tau }^{(l)}$ we can bound

3.30 $$\begin{aligned} \tau \sum _{l = 1}^k \kappa \int _{\partial \Omega }(\theta _{\flat , \tau }^{(l)} - \theta _{\tau }^{(l)}) \textrm{d}\mathcal {H}^{d-1} \le \tau \sum _{l = 1}^k \kappa \int _{\partial \Omega } \theta _{\flat , \tau }^{(l)} \textrm{d}\mathcal {H}^{d-1} = \kappa \int _0^{k \tau } \int _{\partial \Omega } \theta _\flat \textrm{d}\mathcal {H}^{d-1} \textrm{d}t. \end{aligned}$$

We define the piecewise affine function $\hat{y}_\tau (t) = \frac{t - (l-1)\tau }{\tau } y_\tau ^{(l)} + \frac{l\tau - t}{\tau } y_\tau ^{(l-1)}$ for $t \in [(l-1)\tau , l\tau ]$ and $l \in \{ 1, \ldots , k \}$, and note that $\delta _\tau y_{\tau }^{(l)} = \partial _t \hat{y}_\tau (t)$ for $t \in ((l-1)\tau , l\tau )$. Consequently, integration by parts yields

3.31 $$\begin{aligned} \sum _{l = 1}^k \tau ( f_{\tau }^{(l)}, \delta _\tau y_{\tau }^{(l)} )_2&= \int _0^{k\tau } ( f(t), \partial _t \hat{y}_\tau (t))_2 \textrm{d}t = ( f(k\tau ), \hat{y}_\tau (k \tau ) )_2 - ( f (0), y_{0,\varepsilon } )_2 \nonumber \\&\quad - \int _0^{k\tau } ( \partial _t f(t), \hat{y}_\tau (t) )_2 \textrm{d}t \le ( f(k\tau ), \hat{y}_\tau (k \tau ) )_2 - ( f(0), y_{0,\varepsilon } )_2 \nonumber \\&\quad + \int _0^{k\tau } \Vert \partial _t f(t) \Vert _{L^2(\Omega )} \Vert \hat{y}_\tau (t) \Vert _{L^2 (\Omega )} \textrm{d}t. \end{aligned}$$

By Poincaré’s inequality and (W.3) we have for every $t \in ((l-1)\tau , l\tau )$ that

$$\begin{aligned} \Vert \hat{y}_\tau (t) \Vert ^2_{ L^2 (\Omega )} \le C ( \Vert \nabla y_{\tau }^{(l-1)} \Vert ^2_{L^2(\Omega )} + \Vert \nabla y_{\tau }^{(l)} \Vert ^2_{L^2(\Omega )} ) \le C \big (1 + \mathcal {W}^{\textrm{el}}(y_{\tau }^{(l-1)}) + \mathcal {W}^{\textrm{el}}(y_{\tau }^{(l)})\big ). \end{aligned}$$

Therefore, by (3.11), (3.12), the definition of $\mathcal {G}^{(l)}$ in (3.13), and $\sqrt{s} \le 1 + s$ for all $s \ge 0$ we get

$$\begin{aligned}&\int _0^{k \tau } \Vert \partial _t f(t) \Vert _{L^2 (\Omega )} \Vert \hat{y}_\tau (t) \Vert _{L^2 (\Omega )} \textrm{d}t \\&\quad \le C \sum _{l=1}^k \Big ( 1 + \mathcal {W}^{\textrm{el}}(y_{\tau }^{(l-1)}) + \mathcal {W}^{\textrm{el}}(y_{\tau }^{(l)}) \Big ) \int _{(l-1) \tau }^{l \tau } \Vert \partial _t f(t) \Vert _{ L^2 (\Omega )} \textrm{d}t \\&\quad \le C \sum _{l=1}^k \Big ( \big (\mathcal {G}^{(l-1)} + \mathcal {G}^{(l)} \big ) \int _{(l-1)\tau }^{l\tau } \Vert \partial _t f(t) \Vert _{ L^2 (\Omega )} \textrm{d}t \Big ) + C_T (C_{f} + C_{f}^3 ). \end{aligned}$$

Then, using an index shift and $C_{f} \le \frac{2}{3} + \frac{1}{3}C_{f}^3$ we get

$$\begin{aligned}&\int _0^{k\tau } \hspace{-0.1cm} \Vert \partial _{t}{f}(t) \Vert _{L^2(\Omega )} \Vert \hat{y}_\tau (t)\Vert _{L^2(\Omega )} \textrm{d}t \\&\quad \le C \sum _{l=0}^k \Big ( \mathcal {G}^{(l)} \int _{(l-1)\tau }^{l\tau } \big ( \Vert \partial _{t}{f}(t) \Vert _{L^2 (\Omega )} + \Vert \partial _{t}{f}(t + \tau ) \Vert _{L^2 (\Omega )} \big ) \textrm{d}t \Big ) + C_T (1 + C_{f}^3) \end{aligned}$$

for a possibly larger $C_T>0$. Plugging this into (3.31) and using (3.30) to estimate the terms on the right-hand side of (3.29), we conclude the proof by the definition of $\mathcal {G}^{(l)}$ in (3.13). $\square$

#### Proof of Lemma 3.7

Applying Lemma 3.9 and using the nonnegativity of $\mathcal {M}$ we get

3.32 $$\begin{aligned}&\sum _{l = 1}^k \tau \Big ( \int _\Omega \xi (\nabla y_{\tau }^{(l-1)}, \delta _\tau \nabla y_{\tau }^{(l)}, \theta _{\tau }^{(l-1)}) \textrm{d}x + \varepsilon \Vert \delta _{\tau } \nabla \Delta y^{(l)}_{\tau } \Vert _{L^{2}(\Omega )}^{2} \Big ) \nonumber \\  &\le \mathcal {M}(y_{0,\varepsilon }) + \frac{\rho }{2} \Vert y_{0,\varepsilon } ' \Vert _{L^2(\Omega )}^2 - \tau \sum _{l=1}^k \int _\Omega \partial _F W^{\textrm{cpl}}(\nabla y_{\tau }^{(l-1)}, \theta _{\tau }^{(l-1)}) : \delta _\tau \nabla y_{\tau }^{(l)} \textrm{d}x \nonumber \\&+ \tau \sum _{l = 1}^k ( f_{\tau }^{(l)}, \delta _\tau y_{\tau }^{(l)} )_2 + C_M \tau V_k. \end{aligned}$$

As $\mathcal {M}(y_{\tau }^{(l-1)}) \le 2 M + C + C_T C_{f}^2$ for all $l \in \{ 1, \, \ldots , \, k \}$, see the argument in (3.23), employing also Lemma 3.2 we can apply the generalized Korn’s inequality in the form [37, Corollary 3.4], leading to

3.33 $$\begin{aligned} \int _\Omega \xi (\nabla y_{\tau }^{(l-1)}, \delta _\tau \nabla y_{\tau }^{(l)}, \theta _{\tau }^{(l-1)}) \textrm{d}x \ge \frac{1}{C_M} \Vert \delta _\tau \nabla y_{\tau }^{(l)} \Vert _{L^2(\Omega )}^2, \end{aligned}$$

for a constant $C_M$ depending on *M*, *T*, and *f*. By Hölder’s inequality, Poincaré’s inequality, and Young’s inequality with constant $\lambda \in (0, 1)$ we derive that

3.34 $$\begin{aligned} |( f_\tau ^{(l)}, \delta _\tau y_{\tau }^{(l)} )_2| \le C \Vert f_\tau ^{(l)} \Vert _{L^2(\Omega )} \Vert \delta _\tau \nabla y_{\tau }^{(l)} \Vert _{L^2(\Omega )}&\le \frac{C}{\lambda } \Vert f_\tau ^{(l)} \Vert _{L^2 (\Omega )}^2 + \lambda \Vert \delta _\tau \nabla y_{\tau }^{(l)} \Vert _{L^2(\Omega )}^2. \end{aligned}$$

Choosing $\lambda < \frac{1}{2 C_M}$ above, summing over $l \in \lbrace 1,\ldots , k \rbrace$ in (3.33)–(3.34), and plugging into (3.32), we find

3.35 $$\begin{aligned}&\frac{1}{2C_M}V_k + \sum _{l = 1}^k \tau \varepsilon \Vert \delta _{\tau } \nabla \Delta y^{(l)}_{\tau } \Vert _{L^{2}(\Omega )}^{2} = \frac{1}{2C_M} \sum _{l = 1}^k \tau \Vert \delta _\tau \nabla y_{\tau }^{(l)} \Vert _{L^2(\Omega )}^2 + \sum _{l = 1}^k \tau \varepsilon \Vert \delta _{\tau } \nabla \Delta y^{(l)}_{\tau } \Vert _{L^{2}(\Omega )}^{2} \nonumber \\&\le \mathcal {M}(y_{0,\varepsilon } ) + \frac{\rho }{2} \Vert y_{0,\varepsilon } ' \Vert _{L^2(\Omega )}^2 - \tau \sum _{l=1}^k \int _\Omega \partial _F W^{\textrm{cpl}}(\nabla y_{\tau }^{(l-1)}, \theta _{\tau }^{(l-1)}) : \delta _\tau \nabla y_{\tau }^{(l)} \textrm{d}x \nonumber \\&+ C_M \tau V_k + C_M C_T C_{f}^2, \end{aligned}$$

where we also used (3.11). Since $|\partial _F W^\textrm{cpl}(F,\theta )| \le 2C_0(1 + |F|) \le 2C_0(2 + |F|^2)$ for all $F \in GL^+(d)$ and $\theta >0$, see [2, Lemma 3.4], applying Young’s inequality, (W.3), and (2.6) we get for some $C_M^*>0$ that

$$\begin{aligned} \tau \sum _{l=1}^k \int _\Omega&\partial _F W^{\textrm{cpl}}(\nabla y_{\tau }^{(l-1)}, \theta _{\tau }^{(l-1)}) : \delta _\tau \nabla y_{\tau }^{(l)} \textrm{d}x \le \frac{1}{8C_M} \sum _{l = 1}^k \tau \Vert \delta _\tau \nabla y_{\tau }^{(l)} \Vert _{L^2(\Omega )}^2 \\&+ C^*_M \tau \sum _{l = 1}^k \int _\Omega (1+ |\nabla y_{\tau }^{(l-1)}|^2) \, \textrm{d}x \\&\le \frac{1}{8C_M} V_k + C^*_M \tau \sum _{l = 1}^k \big (1+ \mathcal {M}(y_{\tau }^{(l-1)})\big ) \,. \end{aligned}$$

Plugging this into (3.35), and possibly decreasing $\tau _0$ such that $C_M^2 \tau _0 \le \frac{1}{8}$ holds true, we get

$$\begin{aligned}  &   \frac{1}{4C_M}V_k + \sum _{l = 1}^k \tau \varepsilon \Vert \delta _{\tau } \nabla \Delta y^{(l)}_{\tau } \Vert _{L^{2}(\Omega )}^{2} \le \mathcal {M}(y_{0,\varepsilon } ) + \frac{\rho }{2} \Vert y_{0,\varepsilon } ' \Vert _{L^2(\Omega )}^2 \\  &   + C_M \tau \sum _{l = 1}^k \big (1+ \mathcal {M}(y_{\tau }^{(l-1)})\big ) + C_M C_T C_{f}^2 \end{aligned}$$

for $$C_M >0 $$ sufficiently large. Multiplying both sides with $$4C_M$$ then leads to the desired estimate (3.17). $$\square $$

### A priori bounds, compactness, and regularity

Given $y_{\tau }^{(0)}, \ldots , y_{\tau }^{(T / \tau )}$ and $\theta _{\tau }^{(0)}, \ldots , \theta _{\tau }^{(T / \tau )}$ from Proposition 3.5, we define the following interpolations: for $k \in \{ -h/\tau , \ldots , T / \tau \}$, let ${\overline{y}}_\tau (k\tau ) = \underline{y}_\tau (k\tau ) = \hat{y}_\tau (k\tau ) :=y_{\tau }^{(k)}$ and for $t \in ((k-1)\tau , k\tau )$ let

3.36 $$\begin{aligned} \overline{y}_\tau (t)&:=y_{\tau }^{(k)},&\underline{y}_\tau (t)&:=y_{\tau }^{(k-1)},&\hat{y}_\tau (t)&:=\frac{k \tau - t}{\tau } y_{\tau }^{(k-1)} + \frac{t - (k-1)\tau }{\tau } y_{\tau }^{(k)}. \end{aligned}$$

A similar notation is employed for $\overline{\theta }_\tau$, $\underline{\theta }_\tau$, and $\hat{\theta }_\tau$, for $\overline{w}_\tau$, $\underline{w}_\tau$, and $\hat{w}_\tau$, and for $\overline{f}_\tau$. The next proposition lists several a priori bounds for the sequences of interpolations.

#### Proposition 3.10

(A priori bounds and compactness) Let $T, \, h, \, \varepsilon > 0$ and $\tau _0$ be as in Proposition 3.5. Then, there exists a constant *C* only depending on *T*, $y_{0,\varepsilon }$, $y_{0,\varepsilon }'$, $\theta _{0}$, *f*, and $\theta _\flat$ such that for all $\tau \in (0, \tau _0)$ with $T / h \in \mathbb {N}$ and $h / \tau \in \mathbb {N}$ the following bounds hold true:

3.37a $$\begin{aligned} \Vert {\overline{y}}_\tau \Vert _{L^\infty ( I_h; W^{2, p}(\Omega ))} + \Vert \det (\nabla {\overline{y}}_\tau )^{-1} \Vert _{L^\infty ( I_h \times \Omega )}&\le C , \end{aligned}$$

3.37b

3.37c $$\begin{aligned} \Vert \overline{\theta }_\tau \Vert _{L^\infty (I; L^1(\Omega ))} + \Vert {\overline{w}}_\tau \Vert _{L^\infty (I; L^1(\Omega ))}&\le C . \end{aligned}$$

Moreover, for each $q \in [1, \frac{d + 2}{d})$ and $r \in [1, \frac{d+2}{d+1})$ we can find constants $C_q>0$ and $C_r>0$ such that

3.37d $$\begin{aligned} \Vert \overline{\theta }_\tau \Vert _{L^q(I \times \Omega )} + \Vert {\overline{w}}_\tau \Vert _{L^q(I \times \Omega )} \le C_q , \end{aligned}$$

3.37e $$\begin{aligned} \Vert \nabla \overline{\theta }_\tau \Vert _{L^r(I \times \Omega )} + \Vert \nabla {\overline{w}}_\tau \Vert _{L^r(I \times \Omega )} \le C_r. \end{aligned}$$

Moreover, there exist $y_h \in C (I_h; \mathcal {Y}_{\textbf{id}}) \cap H^1( I_h; H^3(\Omega ; \mathbb {R}^d))$ and $\theta _h \in L^1(I; W^{1, 1}(\Omega ))$ with $y_h(t) = y_{0,\varepsilon } + t y_{0,\varepsilon }'$ for all $t \in [-h, 0]$ and $\theta _{h} \ge 0$ a.e. such that, up to taking subsequences (not relabeled), as $\tau \rightarrow 0$ it holds that

3.38a $$\begin{aligned} \hat{y}_\tau&\rightharpoonup y_h \quad \text {weakly in } H^1( I_h; H^3(\Omega ;\mathbb {R}^d)), \end{aligned}$$

3.38b $$\begin{aligned} {\overline{y}}_\tau&\rightarrow y_h \quad \text {strongly in } L^\infty (I_h; W^{1, \infty }(\Omega ; \mathbb {R}^{d})) \ \ \text { and strongly in } L^\infty (I_h; W^{2, p}(\Omega ; \mathbb {R}^{d})), \end{aligned}$$

3.38c $$\begin{aligned} \overline{\theta }_\tau&\rightharpoonup \theta _h \quad \text { and } \quad {\overline{w}}_\tau \rightharpoonup w_h \quad \text {weakly in } L^r(I; W^{1,r}(\Omega )) \text { for any } r \in [1, \tfrac{d+2}{d+1}), \end{aligned}$$

3.38d $$\begin{aligned} \overline{\theta }_\tau&\rightarrow \theta _h \quad \text { and } \quad {\overline{w}}_\tau \rightarrow w_h \quad \text {strongly in } L^s(I \times \Omega ) \text { for any } s \in [1, \tfrac{d+2}{d}), \end{aligned}$$

where $w_h :=W^{\textrm{in}}(\nabla y_h, \theta _h)$. Note that the convergence in (3.38b) also hold true for $\underline{y}_\tau$ or $\hat{y}_\tau$ instead of ${\overline{y}}_\tau$. Moreover, the convergences in (3.38c) and (3.38d) remain true after replacing $\overline{\theta }_\tau$ (${\overline{w}}_\tau$) with $\underline{\theta }_\tau$ ($\underline{w}_\tau$) or $\hat{\theta }_\tau$ ($\hat{w}_\tau$), respectively.

#### Proof

Except for the estimate on $\Vert \partial _{t} \hat{y}_{\tau }\Vert _{L^2( I_h; H^3(\Omega ))}$, the bounds (3.37a)–(3.37c) are a direct consequence of the a priori bounds (3.14)–(3.15) from Proposition 3.5, Lemma 3.2, (2.15), and our definition of the different interpolations in time, where we particularly use that $\hat{y}_{\tau }(t) = y_{0,\varepsilon } + t y_{0,\varepsilon }'$ for $t \in (-h,0)$. The remaining estimate in (3.37b) is based on the bound

3.39 $$\begin{aligned} \Vert \partial _{t} \nabla \hat{y}_{\tau }\Vert _{L^2(I_h \times \Omega )} + \sqrt{\varepsilon } \Vert \partial _{t} \nabla \Delta \hat{y}_{\tau }\Vert _{L^2(I_h \times \Omega )} \le C \end{aligned}$$

provided by (3.15). Elliptic regularity for the operator $\Delta$ and the fact that $\partial _{t} \hat{y}_{\tau } = 0$ on $I_h\times \partial \Omega$ imply

3.40 $$\begin{aligned} \Vert \partial _{t} \hat{y}_{\tau } \Vert _{L^2(I_h; H^3(\Omega ))} \le C \Vert \partial _{t} \Delta \hat{y}_{\tau } \Vert _{L^2(I_h; H^1(\Omega ))}. \end{aligned}$$

Eventually, we use the interpolation inequality $\Vert \Delta v \Vert _{L^2(\Omega )} \le C \Vert \nabla \Delta v \Vert _{L^2(\Omega )} + C \Vert \nabla v \Vert _{L^2(\Omega )}$ for all $v \in H^3(\Omega ;\mathbb {R}^d)$ which can be shown by the standard contradiction-compactness argument. This along with (3.39)–(3.40) indeed yields the remaining bound in (3.37b).

The proof of (3.37d)–(3.37e) relies on a proof of a weighted $L^2$-bound on the temperature gradient, namely

3.41 $$\begin{aligned} \int _0^T \int _\Omega \frac{\eta }{(1 + {\overline{w}}_\tau )^{1 + \eta }} |\nabla {\overline{w}}_\tau |^2 \textrm{d}x \textrm{d}t \le C \end{aligned}$$

for any $\eta \in (0, 1)$, where *C* is a constant only depending on *T*. We refer to e.g. [2, Theorem 3.20] for further details. It thus remains to show (3.41). As noticed in the proof of Proposition 3.4, our thermal step coincides with the one from [2] up to adding the term $\varepsilon |\delta _{\tau } \nabla \Delta y^{(k)}_{\tau } |^{2} \wedge \tau ^{-1}$ to the dissipation $\xi (\nabla y_{\tau }^{(k-1)}, \delta _\tau \nabla y_{\tau }^{(k)}, \theta _{\tau }^{(k-1)})$. The proof of the weighted $L^2$-bound (3.41) in [2, Lemma 3.19] relies only on a uniform $L^1$-bound on the dissipation. In view of (3.37b), a uniform $L^1$-bound (in $\tau$, *h*, and $\varepsilon$) is still available in the current setting and the arguments in [2, Lemma 3.19] also apply here.

The a priori bounds along with a diagonal sequence argument show (3.38a)–(3.38d). More precisely, the convergence (3.38a) is a straightforward consequence of the a priori bound (3.37b) and Banach’s selection principle. The convergences in (3.38b) follow from the embeddings $H^3(\Omega ; \mathbb {R}^d) \subset \subset W^{2, p}(\Omega ; \mathbb {R}^{d}) \subset \subset W^{1, \infty }(\Omega ; \mathbb {R}^{d})$ (recall that $p \in (3, 6)$ for $d=3$), and (3.37b) along with the Aubin-Lions’ lemma. Next, (3.38c) follows from (3.37d)–(3.37e). Eventually, the convergence in (3.38d) and the identification $w_h = W^{\textrm{in}}(\nabla y_h, \theta _h)$ can be shown using the bounds (3.37d)–(3.37e), the Aubin-Lions’ lemma, and interpolation with the bound in (3.37c). For further details we refer, e.g., to [2, Lemma 4.2]. $\square$

We proceed with further regularity results for $\hat{y}_\tau$ which hinge on a special case of elliptic regularity, see Lemma A.1 in the appendix. This will allow us to prove better regularity for the temperature variable, see Corollary 3.13 below, and it will also be instrumental for the passage $\varepsilon \rightarrow 0$ in Section 5.

#### Proposition 3.11

(Higher regularity of the deformation) Let $T, \, h, \, \varepsilon > 0$ and $\tau \in (0,\tau _0)$ be as in Proposition 3.5. Then, $\hat{y}_\tau \in L^2( I; H^4(\Omega ; \mathbb {R}^d))$, $\partial _t\hat{y}_\tau \in L^2(I; H^5(\Omega ; \mathbb {R}^d))$, and for each $t \in I$ the time derivative $\partial _t \hat{y}_\tau$ satisfies the boundary conditions

3.42 $$\begin{aligned} \partial _\nu \Delta \partial _t \hat{y}_\tau (t) = \Delta \partial _t \hat{y}_\tau (t) = \Delta ^{2}\partial _t \hat{y}_\tau (t) = 0 \qquad \mathcal {H}^{d-1}\text {-a.e. in } \partial \Omega . \end{aligned}$$

Note that $\hat{y}_\tau$ only has $H^4$-regularity in space due to the regularity of the initial condition $y_{0,\varepsilon }$, see (2.20). The next lemma provides some useful bounds on $\Delta \hat{y}_{\tau }$ and on $\Delta ^{2} \hat{y}_{\tau }$, which will be particularly crucial for passing to the limit $\varepsilon \rightarrow 0$ in Section 5. Define

3.43 $$\begin{aligned} \varrho = \frac{2p}{4p - (p-2)d } \end{aligned}$$

and note that $\varrho \in (\frac{1}{2},1)$ since $p >2$ for $d=2$ and $p \in (3,6)$ for $d=3$.

#### Lemma 3.12

(Bounds from regularity) Let $T, \, h, \, \varepsilon > 0$. Then, for $\tau _0$ sufficiently small depending on $\varepsilon$ and *h* such that Proposition 3.5 is applicable and for $\tau \in (0,\tau _0)$, there exists a constant $C>0$ only depending on *T*, $y_{0,\varepsilon }$, $y_{0,\varepsilon }'$, $\theta _{0}$, *f*, and $\theta _\flat$ such that

3.44 $$\begin{aligned}&\Vert \Delta \overline{y}_{\tau } \Vert _{L^{2} (I; H^{1}(\Omega ))} + \Vert |\Delta \overline{y}_{\tau }|^{\frac{p-2}{2}} \nabla (\Delta \overline{y}_{\tau }) \Vert _{L^{2} (I \times \Omega )} \le C \big ( 1 + \sqrt{\varepsilon } \Vert y_{0,\varepsilon }\Vert _{H^{4}(\Omega )} + \Vert y'_{0, \varepsilon }\Vert _{H^{1}( \Omega )} \big ), \end{aligned}$$

3.45 $$\begin{aligned}&\Vert \, \Delta ^{2}\hat{y}_{\tau } \Vert _{L^{2}(I \times \Omega )} \le C \varepsilon ^{-1/2} \big ( 1 + \sqrt{\varepsilon } \Vert y_{0, \varepsilon }\Vert _{H^{4}(\Omega )} + \Vert y'_{0, \varepsilon }\Vert _{H^{1}(\Omega )} \big ) , \end{aligned}$$

3.46 $$\begin{aligned}&\Vert \nabla {D H}(\Delta \overline{y}_\tau )\Vert _{L^2(I;L^{p'}(\Omega ))} \le C \big ( 1 + \sqrt{\varepsilon } \Vert y_{0, \varepsilon }\Vert _{H^{4}(\Omega )} + \Vert y'_{0, \varepsilon }\Vert _{H^{1}(\Omega )} \big ) , \end{aligned}$$

3.47 $$\begin{aligned}&\Vert \, \Delta \partial _{t} \hat{y}_{\tau } \Vert _{L^{2}(I;H^2(\Omega ))} \le C \varepsilon ^{-\frac{1+\varrho }{2}} \big ( 1 + \root 4 \of {\varepsilon } \Vert y_{0, \varepsilon }\Vert _{H^{4}(\Omega )} + \Vert y'_{0, \varepsilon }\Vert _{H^{1}(\Omega )} \big ). \end{aligned}$$

We postpone the proofs of the two results to the end of the subsection and first present the following consequence.

#### Corollary 3.13

(Further a priori bounds) Let $T, \, h, \, \varepsilon > 0$ and $\tau _0$ be as in Proposition 3.5. Then, there exists a constant $C_\varepsilon >0$ only depending on *T*, $\varepsilon$, $y_{0,\varepsilon }$, $y_{0,\varepsilon }'$, $\theta _{0}$, *f*, and $\theta _\flat$ such that for all $\tau \in (0, \tau _0)$ with $T / h \in \mathbb {N}$ and $h / \tau \in \mathbb {N}$ the following bounds hold true:

3.48 $$\begin{aligned} \Vert \overline{\theta }_\tau \Vert _{L^2(I; H^1(\Omega ))} + \Vert {\overline{w}}_\tau \Vert _{L^2(I;H^1(\Omega ))}&\le C_\varepsilon , \end{aligned}$$

3.49 $$\begin{aligned} \Vert \hat{w}_\tau \Vert _{H^1(I; (H^1(\Omega ))^*)}&\le C_\varepsilon ,\end{aligned}$$

3.50 $$\begin{aligned} \Vert \hat{y}_\tau \Vert _{L^2(I; H^4(\Omega ))}&\le C_\varepsilon . \end{aligned}$$

In particular, $\theta _h$ and $w_h$ from Proposition 3.10 satisfy $\theta _h, w_h \in L^2(I; H^1(\Omega ))$ and $w_h\in H^1(I; (H^1(\Omega ))^*)$ with $w_h(0) = W^{\textrm{in}}(\nabla y_{0,\varepsilon }, \theta _0)$. Moreover, $y_h$ lies in $L^\infty ( I; \mathcal {Y}^\textrm{reg}_{\textbf{id}})$.

#### Proof

First, by elliptic regularity along with the boundary conditions $\hat{y}_\tau = \textbf{id}$ and $\Delta \hat{y}_\tau = 0$ on $I_h \times \partial \Omega$ (see (2.1), (2.20), and (3.42)), and the fact that $\Omega$ has $C^5$-boundary, we get

$$\begin{aligned} \Vert \hat{y}_\tau (t) - \textbf{id}\Vert _{H^4(\Omega )} \le C \Vert \Delta \hat{y}_\tau (t) \Vert _{H^2(\Omega )}, \quad \quad \Vert \Delta \hat{y}_\tau (t) \Vert _{H^2(\Omega )} \le C \Vert \Delta ^2 \hat{y}_\tau (t) \Vert _{L^2(\Omega )} \end{aligned}$$

for a.e. $t \in I_h$. This along with (3.45) shows (3.50). Moreover, $y_h \in L^\infty ( I; \mathcal {Y}^\textrm{reg}_{\textbf{id}})$ (see (2.20)) follows from (3.37b), (3.42), (3.47), and the fact that $y_{0,\varepsilon } \in \mathcal {Y}^\textrm{reg}_{\textbf{id}}$.

Due to (3.47), we find that $\varepsilon | \partial _t \nabla \Delta \hat{y}_\tau |^{2}$ is bounded in $L^2(I_h;L^2(\Omega ))$ for a bound depending on $\varepsilon$, but independent of $\tau$ and *h*. Therefore, the term $\partial _F W^{\textrm{cpl}}(\nabla \underline{y}_\tau , \underline{\theta }_\tau ) : \partial _t \nabla \hat{y}_\tau + \xi (\nabla \underline{y}_\tau , \partial _t \nabla \hat{y}_\tau , \underline{\theta }_\tau ) + \varepsilon |\partial _t \nabla \Delta \hat{y}_\tau |^{2} \wedge \tau ^{-1}$ appearing in the second line of (3.9) is bounded in $L^{2} (I_{h};L^2(\Omega ))$ for a bound depending on $\varepsilon$, but independent of $\tau$ and *h*. This regularity allows us to apply the a priori estimates in [37, Proposition 4.2]. This yields (3.48)–(3.49), and then the regularity of the limits $\theta _h$ and $w_h$ is a direct consequence of weak compactness. By [4, Lemma 4.5(iii)] we get $\theta _h, w_h \in C(I;L^2(\Omega ))$ which along with (C.1) and $y_h \in C (I_h; \mathcal {Y}_{\textbf{id}})$ also shows $w_h(0) = W^{\textrm{in}}(\nabla y_{0,\varepsilon }, \theta _0)$. This concludes the proof. $\square$

We now come to the proofs of Proposition 3.11 and Lemma 3.12. As they are purely of technical nature, the reader might want to skip these proofs on first reading of the paper.

#### Proof of Proposition 3.11

We recall the notation in (3.36) and for convenience we drop the index $\tau$ in the entire proof. As a preliminary step, we first show $\hat{y} \in H^1(I; H^4 (\Omega ; \mathbb {R}^d))$, and afterwards the statement.

*Step 1* ($\hat{y} \in H^1(I; H^4 (\Omega ; \mathbb {R}^d))$): We show that, if $\overline{y} \in L^2(I; W^{2,q} (\Omega ; \mathbb {R}^d))$ for some $q \in [ p, 2(p-1)]$, then

3.51 $$\begin{aligned} \hat{y} \in H^1(I; W^{4,\frac{q}{p-1}} (\Omega ; \mathbb {R}^d)), \quad \quad \overline{y} \in L^2(I; W^{2,q(1+\eta )} (\Omega ; \mathbb {R}^d)), \end{aligned}$$

where $\eta :=\infty$ for $d=2$ and $\eta :=\frac{5}{p-1} - 1>0$ for $d=3$, as well as

3.52 $$\begin{aligned} \partial _\nu \Delta \partial _t \hat{y}(t) = 0 \qquad \mathcal {H}^{d-1}\text {-a.e. on } \partial \Omega . \end{aligned}$$

Once this has been shown, this argument can first be applied for $$q = p $$ by (3.37a), and then by a bootstrapping argument, after a finite number of repetitions depending on $$\eta $$, we get $$\hat{y} \in H^1(I; H^4 (\Omega ; \mathbb {R}^d))$$.

Let us show (3.51)–(3.52). Suppose that $\overline{y} \in L^2(I; W^{2,q} (\Omega ; \mathbb {R}^d))$ for some $q \in [ p, 2(p-1)]$. For $t \in I$, we define $g^\textrm{3rd}(t) \in X_q^*$ in the dual space of $X_q:=W^{2,(\frac{q}{p-1})'}(\Omega ; \mathbb {R}^d) \cap H^1_0(\Omega ; \mathbb {R}^d)$ by

3.53 $$\begin{aligned} \langle g^\textrm{3rd}(t), z \rangle&:=\int _\Omega D H(\Delta \overline{y}(t)) \cdot \Delta z + \Big ( \partial _F W(\nabla \overline{y}(t), \underline{\theta }(t)) + \partial _{\dot{F}} R(\nabla \underline{y}(t), \partial _t \nabla \hat{y}(t), \underline{\theta }(t)) \Big ) : \nabla z \textrm{d}x \nonumber \\&\quad - \int _\Omega \overline{f}(t) \cdot z \textrm{d}x + \frac{\rho }{h} \int _{\Omega } (\partial _t \hat{y}(t) - \partial _t \hat{y}(t - h)) \cdot z \textrm{d}x \end{aligned}$$

for all $z \in X_q$. By (H.3) and the assumption $\overline{y} \in L^2 (I;W^{2,q}(\Omega ;\mathbb {R}^d))$ we get $D H(\Delta \overline{y}) \in L^2 (I;L^{\frac{q}{p-1}}(\Omega ;\mathbb {R}^d))$ and thus $g^\textrm{3rd} \in L^2(I;X_q^*)$ by the bounds from Proposition 3.10. Fixing $z \in X_q \cap H^3(\Omega ;\mathbb {R}^d)$ and using *z* as a test function in (3.6), we derive that

3.54 $$\begin{aligned}&-\varepsilon \int _{\Omega } \nabla \Delta \partial _t \hat{y}(t) : \nabla \Delta z \textrm{d}x = \langle g^\textrm{3rd}(t), z \rangle , \end{aligned}$$

i.e., $$g^\textrm{3rd}$$ represents the regularization of third order. Due to (3.54), for every $$t \in I$$ we can apply Lemma A.1(a) for $$u :=\partial _t \hat{y}(t)$$. With (A.2)–(A.3) this shows $$\partial _t \hat{y} \in L^2( I; W^{4,\frac{q}{p-1}} (\Omega ; \mathbb {R}^d))$$ as well as (3.52). As $$y_{0, \varepsilon } \in \mathcal {Y}^\textrm{reg}_{\textbf{id}}$$, the above statements together with (2.20) directly lead to $$\hat{y} \in H^1(I; W^{4,\frac{q}{p-1}} (\Omega ; \mathbb {R}^d))$$. By Sobolev embedding we get that $$\overline{y} \in L^2(I; W^{2, r} (\Omega ; \mathbb {R}^d))$$, where $$r = (\frac{q}{p-1})^{**}$$. Since $$q \ge p$$ and thus $$\frac{q}{p-1} \ge \frac{6}{5}$$, we get $$r=\infty $$ for $$d=2$$ and for $$d=3$$ we have $$r = (3\frac{q}{p-1})(3-2\frac{q}{p-1})^{-1} \ge \frac{5q}{p-1} = q (1+\eta )$$. This concludes the proof of (3.51).

*Step 2 (Proof of the statement):* From now on we can suppose that $\hat{y} \in H^1(I; H^4 (\Omega ; \mathbb {R}^d))$. Due to this improved regularity of $\hat{y}$, we have for each $t \in I$ and any $z \in C^\infty _c(\Omega ; \mathbb {R}^d)$ that

3.55 $$\begin{aligned} \int _\Omega D H(\Delta \overline{y} (t) ) \cdot \Delta z \textrm{d}x = -\int _\Omega \nabla ( D H(\Delta \overline{y} (t) )) : \nabla z \textrm{d}x. \end{aligned}$$

Recalling (2.4)–(2.5), an elementary computation for a general $v \in H^4 (\Omega ;\mathbb {R}^d)$ yields that pointwise a.e. in $\Omega$ it holds that

3.56 $$\begin{aligned} \nabla ( D H(\Delta v)) = {\left\{ \begin{array}{ll} 2 \nabla \Delta v & \text {if } p |\Delta v|^{p-2} \le 2, \\ p (p-2) |\Delta v|^{p-4} \Delta v \otimes \big ( ( \nabla \Delta v)^T \Delta v \big ) + p |\Delta v|^{p-2} \nabla \Delta v & \text {else}. \end{array}\right. } \end{aligned}$$

Taking the Frobenius norm on both sides of (3.56) we see a.e. in $\Omega$ that

3.57 $$\begin{aligned} |\nabla ( D H(\Delta v))| \le {\left\{ \begin{array}{ll} 2 |\nabla \Delta v| & \text {if } p |\Delta v|^{p-2} \le 2, \\ p (p-1) |\Delta v|^{p-2} |\nabla \Delta v| & \text {else}. \end{array}\right. } \end{aligned}$$

By Sobolev embedding we have that $H^2(\Omega ; \mathbb {R}^d) \subset L^\infty (\Omega ; \mathbb {R}^d)$ for $d=2,3$. Hence, $\Delta \overline{y} \in L^\infty (I \times \Omega ; \mathbb {R}^d)$. In particular, this shows $\nabla ( D H(\Delta \overline{y})) \in L^2(I \times \Omega ; \mathbb {R}^{d\times d})$ and therefore, recalling the definition in (3.53) and (3.55), we derive $g^\textrm{3rd} \in L^2(I; H^{-1}(\Omega ; \mathbb {R}^d))$ by arbitrariness of *z*, where we have used that $C^\infty _c(\Omega ; \mathbb {R}^d)$ is dense in $H^1_0(\Omega ; \mathbb {R}^d)$. (Now, $\langle \cdot , \cdot \rangle$ stands for the duality pairing between $H^{1}_0(\Omega ; \mathbb {R}^d)$ and $H^{-1}(\Omega ; \mathbb {R}^d)$.) By Lemma A.1(b), (3.52), and the bounds from Proposition 3.10 we derive that $\partial _t \hat{y} \in L^2(I; H^5(\Omega ; \mathbb {R}^d))$ and that $\partial _t \hat{y}$ satisfies

3.58 $$\begin{aligned} \Vert \partial _t \hat{y} \Vert _{L^2(I; H^5(\Omega ))} \le C_{\varepsilon ,h} \end{aligned}$$

for a constant $C_{\varepsilon ,h}$ depending on $\varepsilon$ and *h*. Moreover, we have the boundary condition

3.59 $$\begin{aligned} \Delta ^{2}\partial _t \hat{y}(t) = 0 \qquad \mathcal {H}^{d-1}\text {-a.e. on } \partial \Omega \ \ \text {for }t \in I. \end{aligned}$$

To conclude the proof, it remains to show $\Delta \partial _t \hat{y}(t) = 0$ $\mathcal {H}^{d-1}$-a.e. on $\partial \Omega$ for $t \in I$. Defining for $z \in H:=H^3(\Omega ;\mathbb {R}^d) \cap H^1_0(\Omega ;\mathbb {R}^d)$

$$\begin{aligned} \langle g^\textrm{2nd}(t), z \rangle&:=-\varepsilon \int _{\Omega } \nabla \Delta \partial _{t} \hat{y}(t) : \nabla \Delta z \textrm{d}x \\&- \int _\Omega \Big ( \partial _F W(\nabla \overline{y}(t), \underline{\theta }(t)) + \partial _{\dot{F}} R(\nabla \underline{y}(t), \partial _t \nabla \hat{y}(t), \underline{\theta }(t)) \Big ) : \nabla z \textrm{d}x \\&\phantom {\quad =}\quad + \int _\Omega \overline{f}(t) \cdot z \textrm{d}x - \frac{\rho }{h} \int _{\Omega } (\partial _t \hat{y}(t) - \partial _t \hat{y}(t - h)) \cdot z \textrm{d}x, \end{aligned}$$

we can rewrite (3.53)–(3.54) as

3.60 $$\begin{aligned} \int _\Omega D H(\Delta \overline{y}(t)) \cdot \Delta z \textrm{d}x = \langle g^\textrm{2nd}(t), z \rangle \end{aligned}$$

for $t \in I$, i.e., $g^\textrm{2nd}$ represents the term with second derivative. Using the improved regularity of $\partial _t\hat{y}$ and the boundary conditions (3.52) and (3.59), the first integral in the definition of $g^\textrm{2nd}(t)$ can be written as

$$\begin{aligned} \int _\Omega \nabla \Delta \partial _t \hat{y}(t) : \nabla \Delta z \textrm{d}x&= - \int _\Omega \Delta ^{2}\partial _t \hat{y}(t) \cdot \Delta z \textrm{d}x + \int _{\partial \Omega } \partial _\nu \Delta \partial _t \hat{y}(t) \cdot \Delta z \textrm{d}\mathcal {H}^{d-1} \\&= \int _\Omega \nabla \Delta ^{2}\partial _t \hat{y}(t) : \nabla z \textrm{d}x - \int _{\partial \Omega } \Delta ^{2}\partial _t \hat{y}(t) \cdot \partial _\nu z \textrm{d}\mathcal {H}^{d-1} \\&= \int _\Omega \nabla \Delta ^{2}\partial _t \hat{y}(t) : \nabla z \textrm{d}x \end{aligned}$$

for each $z \in H$. As $\partial _t \hat{y} \in L^2(I; H^5(\Omega ; \mathbb {R}^d))$, this shows $g^\textrm{2nd}(t) \in H^{-1}(\Omega ; \mathbb {R}^d)$ for each $t \in I$. Now, it is standard to find $v(t) \in H^1_0(\Omega ;\mathbb {R}^d)$ such that $\langle g^\textrm{2nd}(t), z \rangle = \int _\Omega v(t) \cdot \Delta z \textrm{d}x$ for all $z \in H^2(\Omega ;\mathbb {R}^d ) \cap H^1_0(\Omega ;\mathbb {R}^d)$, see (A.16)–(A.18) below for details. Given an arbitary $\varphi \in L^2(\Omega ;\mathbb {R}^d)$ and choosing $z \in H^2(\Omega ;\mathbb {R}^d) \cap H^1_0(\Omega ;\mathbb {R}^d)$ with $\Delta z = \varphi$, this along with (3.60) shows

$$\begin{aligned} \int _\Omega \big ( D H(\Delta \overline{y}(t)) - v(t) \big ) \cdot \varphi \textrm{d}x = 0. \end{aligned}$$

This yields $D H(\Delta \overline{y}(t)) = v(t)$ a.e. in $\Omega$ and thus it holds that ${D H}(\Delta \bar{y} (t)) = 0$ $\mathcal {H}^{d-1}$-a.e. on $\partial \Omega$ for $t \in I$. Since

$$\begin{aligned} {D H}(v) = \max \{2, p |v|^{p-2}\}v = 0 \ \Leftrightarrow \ v = 0 \in \mathbb {R}^d \end{aligned}$$

and $y_{0,\varepsilon } \in \mathcal {Y}^\textrm{reg}_{\textbf{id}}$, this concludes the proof of (3.42). $\square$

#### Proof of Lemma 3.12

For notational convenience, we define the weighted Bochner space

$$\begin{aligned} \Vert \cdot \Vert _{L^2_T(I; L^q(\Omega ))} :=\Vert \sqrt{T-t} \, \cdot \Vert _{L^2(I; L^q(\Omega ))}, \end{aligned}$$

and employ a similar notation for Sobolev spaces. We first note that it is not restrictive to establish the bounds (3.44)–(3.47) only for the weighted space. Indeed, by extending $$\theta _\flat $$ and *f* suitably on the time interval $$[T,T+\eta ]$$ for some $$\eta >0$$ small, we can establish time-discrete solutions on the time interval $$[-h,T+\eta ]$$, see Proposition 3.5. Then, a control on $$\Vert \cdot \Vert _{L^2_{T+\eta }([0,T+\eta ]; L^q(\Omega ))}$$ will directly imply a control on $$\Vert \cdot \Vert _{L^2(I; L^q(\Omega ))}$$ for a constant additionally depending on $$\eta $$. To simplify notation, we use the interval $$ I= [0,T]$$ instead of $$[0,T+\eta ]$$ in the sequel. As in the proof of Proposition 3.11, we omit the index $$\tau $$.

*Step 1 (Proof of* (3.44) *and* (3.45))*:* As $\partial _t \hat{y} \in L^2(I; H^4(\Omega ; \mathbb {R}^d))$ with $\partial _\nu \Delta \partial _t \hat{y}(t) = 0$ $\mathcal {H}^{d-1}$-a.e. on $\partial \Omega$ for $t \in I$ by Proposition 3.11, by an integration by part we can rewrite the $\varepsilon$-dependent term in (3.6) as

3.61 $$\begin{aligned} \int _\Omega \varepsilon \nabla \Delta \partial _t \hat{y} : \nabla \Delta z \textrm{d}x&= - \int _\Omega \varepsilon \Delta ^{2}\partial _t \hat{y} : \Delta z \textrm{d}x + \int _{\partial \Omega }\varepsilon \partial _\nu \Delta \partial _t \hat{y} : \Delta z \textrm{d}\mathcal {H}^{d-1} \nonumber \\&= - \int _\Omega \varepsilon \Delta ^{2}\partial _t \hat{y} : \Delta z \textrm{d}x \end{aligned}$$

for each $t \in ((k-1)\tau , k\tau )$ and $z \in H^3(\Omega ;\mathbb {R}^d) \cap H^1_0(\Omega ;\mathbb {R}^d)$. Thus, by approximation we can test (3.6) with functions in $H^2(\Omega ;\mathbb {R}^d) \cap H^1_0(\Omega ;\mathbb {R}^d)$. As $\Delta \hat{y} \in L^2(I; H^2(\Omega ; \mathbb {R}^d))$ with $\Delta \hat{y}(t) = 0$ $\mathcal {H}^{d-1}$-a.e. on $\partial \Omega$ for $t \in I$ by Proposition 3.11 and the fact that $y_{0,\varepsilon } \in \mathcal {Y}^\textrm{reg}_{\textbf{id}}$ (see (2.20)), we discover that $z(t, x) :=(T - t)\Delta \hat{y}(t,x)$ for $t \in ((k-1)\tau , k\tau )$ is a valid test function in (3.6). After summation and rearranging terms, and employing (3.61) this yields

3.62 $$\begin{aligned} \begin{aligned}&\varepsilon \int _I \int _{\Omega } \Delta ^{2}\partial _t \hat{y} : \Delta ^{2}\hat{y} (T - t) \textrm{d}x \textrm{d}t - \int _I\int _\Omega D H(\Delta \overline{y}) \cdot \Delta ^{2}\hat{y} (T - t) \textrm{d}x \textrm{d}t \\&\quad = \int _I\int _\Omega \big ( \partial _F W(\nabla \overline{y}, \underline{\theta }) + \partial _{\dot{F}} R(\nabla \underline{y}, \partial _t \nabla \hat{y}, \underline{\theta }) \big ) : \nabla \Delta \hat{y} (T - t) \textrm{d}x \textrm{d}t \\&\phantom {\quad =}\quad - \int _I\int _\Omega \overline{f} \cdot \Delta \hat{y}(t) (T - t) \textrm{d}x \textrm{d}t + \frac{\rho }{h} \int _I \int _\Omega \big (\partial _t \hat{y}(t) - \partial _t \hat{y}(t - h)\big ) \cdot \Delta \hat{y}(t) (T - t) \textrm{d}x \textrm{d}t. \end{aligned} \end{aligned}$$

(The advantage of multiplying with $(T-t)$ will become apparent in (3.68) below.) Using (3.42) and $y_{0,\varepsilon } \in \mathcal {Y}^\textrm{reg}_{\textbf{id}}$ we integrate by parts in the second term on the left-hand side above, which leads to

$$\begin{aligned}&-\int _I\int _\Omega D H(\Delta \overline{y}) \cdot \Delta ^{2}\hat{y}(T - t) \textrm{d}x \textrm{d}t \\&\quad = \int _I\int _\Omega \nabla ( D H(\Delta \overline{y})) : \nabla \Delta \hat{y} (T - t) \textrm{d}x \textrm{d}t - \int _I \int _{\partial \Omega } D H(\Delta \overline{y}) \cdot \partial _\nu \Delta \hat{y} (T - t) \textrm{d}\mathcal {H}^{d-1} \textrm{d}t \\&\quad \ge \int _I\int _\Omega \nabla ( D H(\Delta \overline{y})) : \nabla \Delta \overline{y} (T - t) \textrm{d}x \textrm{d}t - C\tau \Vert \nabla ( D H(\Delta \overline{y})) \Vert _{L^2(I \times \Omega )} \Vert \nabla \Delta \partial _t \hat{y} \Vert _{L^2(I \times \Omega )}, \end{aligned}$$

where in the last step we exploited the definition in (3.36). In view of (3.56), we get that

$$\begin{aligned}&\int _I\int _\Omega \nabla ( D H(\Delta \overline{y})) : \nabla \Delta \overline{y} (T - t) \textrm{d}x \textrm{d}t \\&= \int _I\int _\Omega \max \{2, p |\Delta \overline{y}|^{p-2} \} |\nabla \Delta \overline{y}|^2 (T - t) \textrm{d}x \textrm{d}t \\&+ \int _I\int _\Omega 1\!\!1_{\{p |\Delta \overline{y}|^{p-2} \ge 2\}} p(p-2) |(\nabla \Delta \overline{y})^{T} \Delta \overline{y}|^{2} |\Delta \overline{y}|^{p-4} (T - t) \, \textrm{d}x \, \textrm{d}t \\&\ge \int _I\int _\Omega \max \{2, p |\Delta \overline{y}|^{p-2} \} |\nabla \Delta \overline{y}|^2 (T - t) \textrm{d}x \textrm{d}t. \end{aligned}$$

By $y_{0,\varepsilon } \in \mathcal {Y}^\textrm{reg}_{\textbf{id}}$ and (3.58) we find $\Vert \Delta \overline{y} \Vert _{L^\infty (I;L^\infty (\Omega ))} \le C_{\varepsilon ,h}$ and $\Vert \overline{y} \Vert _{L^2(I; H^3(\Omega ))} \le C_{\varepsilon ,h}$. Then, again using (3.56)–(3.57) and (3.37b) we eventually find

3.63 $$\begin{aligned}&-\int _I\int _\Omega D H(\Delta \overline{y}) \cdot \Delta ^{2}\hat{y}(T - t) \textrm{d}x \textrm{d}t \nonumber \\&\quad \ge \int _I\int _\Omega \max \{2, p |\Delta \overline{y}|^{p-2} \} |\nabla \Delta \overline{y}|^2 (T - t) \textrm{d}x \textrm{d}t - \tau C_{\varepsilon ,h}. \end{aligned}$$

By the chain rule we write

3.64 $$\begin{aligned}&\int _I \int _{\Omega } \Delta ^{2}\partial _t \hat{y} \cdot \Delta ^{2}\hat{y} (T - t) \textrm{d}x \textrm{d}t \nonumber \\&\quad = \int _I \frac{\textrm{d}}{\textrm{d}t}\Big ( \frac{1}{2} \int _{\Omega } |\Delta ^{2}\hat{y}|^2 (T - t) \textrm{d}x \Big ) \textrm{d}t + \frac{1}{2} \int _I \int _{\Omega } |\Delta ^{2}\hat{y}|^2 \textrm{d}x \textrm{d}t \nonumber \\&\quad = \frac{1}{2} \int _I \int _{\Omega } |\Delta ^{2}\hat{y}|^2 \textrm{d}x \textrm{d}t - \frac{T}{2} \Vert \Delta ^{2}y_{0, \varepsilon }\Vert _{L^2(\Omega )}^2\,. \end{aligned}$$

By the definition of $\mathcal {Y}_{\textbf{id}}$ and $\hat{y} \in L^\infty ( I_h; \mathcal {Y}_{\textbf{id}})$, it follows that $\partial _t \hat{y}(t) = 0$ $\mathcal {H}^{d-1}$-a.e. in $\partial \Omega$ for $t \in I$. Hence, integrating by parts leads to

$$\begin{aligned}&\int _I \int _\Omega (\partial _t \hat{y}(t) - \partial _t \hat{y}(t - h)) \cdot \Delta \hat{y} (t) (T - t) \textrm{d}x \textrm{d}t \\&= - \int _I \int _\Omega (\partial _t \nabla \hat{y}(t) - \partial _t \nabla \hat{y}(t - h)) : \nabla \hat{y}(t) (T - t) \textrm{d}x \textrm{d}t. \end{aligned}$$

Now, using the substitution $t \mapsto t - h$ we derive3.65where

$$\begin{aligned}  &   \Pi :=\rho \int _0^{T - h} (T-t) \int _\Omega \partial _t \nabla \hat{y}(t) : \frac{\nabla \hat{y}(t + h) - \nabla \hat{y}(t)}{h} \textrm{d}x \textrm{d}t - \rho \int _0^{T-h} \\  &   \quad \int _\Omega \partial _{t} \nabla \hat{y} (t) :\nabla \hat{y} (t+h) \textrm{d}x \textrm{d}t. \end{aligned}$$

Using that  and applying Hölder’s inequality, we can check that

$$\begin{aligned} |\Pi | \le C\Vert \partial _{t} \nabla \hat{y} \Vert ^2_{L^{2}(I \times \Omega )} + C\Vert \partial _{t} \nabla \hat{y} \Vert _{L^{2}(I \times \Omega )} \Vert \nabla \hat{y} \Vert _{L^{2}(I \times \Omega )} \le C\Vert \nabla \hat{y} \Vert ^2_{H^{1}(I; L^{2} (\Omega ))}. \end{aligned}$$

Combining (3.62)–(3.65) then leads to3.66In view of the bounds (3.37a)–(3.37e), we have that

3.67 $$\begin{aligned}&\sup _{h>0, \, \varepsilon \in (0, 1)} \Vert \partial _{F} W(\nabla \overline{y}, \underline{\theta }) + \partial _{\dot{F}} R(\nabla \underline{y}, \partial _{t} \nabla \hat{y}, \underline{\theta }) \Vert _{L^{2} (I \times \Omega )}<+\infty \,,\nonumber \\&\sup _{h>0, \, \varepsilon \in (0, 1)} \Vert \overline{y}\Vert _{L^{\infty } (I; W^{2, p} (\Omega ))} + \Vert \hat{y}\Vert _{H^1 (I; H^{1}(\Omega ))} <+\infty \,, \end{aligned}$$

where we use (W.1), (C.4), and (D.1). Moreover, we choose $\tau _0$ small enough such that $\tau _0 C_{\varepsilon , h} \le 1$. Hence, by Hölder’s inequality, the weighted Young’s inequality, and by the boundary condition $\Delta \hat{y} (t) = 0$ for a.e. $t \in [0, T]$ and $\mathcal {H}^{d-1}$-a.e. in $\partial \Omega$ (see (2.20) and Proposition 3.11), we infer from (3.66) that

3.68 $$\begin{aligned} \sqrt{\varepsilon } \Vert \Delta ^{2}\hat{y} \Vert _{L^{2}(I \times \Omega )} + \Vert \Delta \overline{y} \Vert _{L^{2}_T (I; H^{1}(\Omega ))} + \Vert \, |\Delta \overline{y}|^{\frac{p-2}{2}} \nabla (\Delta \overline{y}) \Vert _{L^{2}_T (I; L^2(\Omega ))} \nonumber \\ \le C \big ( 1 + \sqrt{\varepsilon } \Vert y_{0, \varepsilon }\Vert _{H^4(\Omega )} + \Vert y'_{0, \varepsilon }\Vert _{H^{1}(\Omega )} \big ) \end{aligned}$$

for some constant $C>0$ independent of $\varepsilon \in (0, 1)$ and $h>0$, where the notation $L^{2}_T (I; H^{1}(\Omega ))$ has been introduced at the beginning of the proof. Here, the factor $(T-t)$ guarantees that the last term on the right-hand side of (3.66) can be controlled uniformly in *h*. This shows (3.44) and (3.45).

*Step 2 (Proof of* (3.46))*:* We proceed with (3.46). In view of (3.57), it holds that

$$\begin{aligned} | \, \nabla ({D H}(\Delta \overline{y}) ) |&\le C \big (1+|\Delta \overline{y}|\big )^{\frac{p-2}{2}} \, \Big |\big (1+|\Delta \overline{y}|\big )^{\frac{p-2}{2}} \nabla \Delta \overline{y} \Big | \end{aligned}$$

a.e. in $\Omega$. By using Hölder’s inequality for $\frac{p'}{2}+\frac{p-2}{2(p-1)}=1$ we estimate

3.69 $$\begin{aligned} \Vert \nabla {D H}(\Delta \overline{y})\Vert _{L^2_T(I;L^{p'}(\Omega ))}^2&\le C \int _I\bigg (\int _\Omega \big (1+ |\Delta \overline{y}|\big )^{\frac{(p-2)p}{2(p-1)}}\big (\sqrt{T - t} \,\big (1+|\Delta \overline{y}|\big )^{\frac{p-2}{2}} |\nabla \Delta \overline{y}|\big )^{p'}\, \textrm{d}x\bigg )^\frac{2}{p'}\, \textrm{d}t \nonumber \\&\le C \Vert 1+|\Delta \overline{y}|\Vert _{L^\infty (I;L^p(\Omega ))}^{p-2}\Vert (1+ |\Delta \overline{y}|)^{\frac{p-2}{2}} \nabla \Delta \overline{y}\Vert _{L^2_T(I; L^2(\Omega ))}^2. \end{aligned}$$

Note that $|\Delta \overline{y}|$ is uniformly bounded in $L^{\infty } (I; L^{p}(\Omega ; \mathbb {R}^{d}))$ by (3.37a). Hence, (3.46) follows from (3.68).

*Step 3 (Proof of* (3.47))*:* Finally, we prove (3.47). In view of Corollary 3.11, we may test the mechanical equation (3.6) with $z = (T - t) \partial _{t}\Delta \hat{y}(t)$ for $t \in ((k-1)\tau , k\tau )$. After summation and rearranging terms this yields

$$\begin{aligned}&-\varepsilon \int _I \int _{\Omega }\partial _{t} \nabla \Delta \hat{y} : \partial _{t} \nabla \Delta ^{2}\hat{y}(T - t) \textrm{d}x \textrm{d}t - \int _I\int _\Omega DH(\Delta \overline{y}) \cdot \partial _{t}\Delta ^{2}\hat{y} (T - t) \textrm{d}x \textrm{d}t \\&\quad = \int _I\int _\Omega \big ( \partial _F W(\nabla \overline{y}, \underline{\theta }) + \partial _{\dot{F}} R(\nabla \underline{y}, \partial _t \nabla \hat{y}, \underline{\theta }) \big ) : \partial _{t} \nabla \Delta \hat{y} (T - t) \textrm{d}x \textrm{d}t \\&\phantom {\quad =}\quad - \int _I\int _\Omega \overline{f} \cdot \partial _{t} \Delta \hat{y} (T - t) \textrm{d}x \textrm{d}t + \frac{\rho }{h} \int _I \int _\Omega (\partial _t \hat{y}(t) - \partial _t \hat{y}(t - h)) \cdot \partial _{t} \Delta \hat{y}(t) (T - t) \textrm{d}x \textrm{d}t. \end{aligned}$$

Integrating by parts the left-hand side and the last term on the right-hand side above, by the boundary conditions $\partial _{\nu } \Delta \partial _{t} \hat{y} = 0$ and $\partial _{t} \hat{y}= 0$ on $\partial \Omega$ for $t \in I$, we get that

3.70 $$\begin{aligned} \begin{aligned}&\varepsilon \int _I \int _{\Omega }| \partial _{t} \Delta ^{2}\hat{y} |^{2} (T - t) \textrm{d}x \textrm{d}t + \int _I\int _\Omega \nabla ( DH(\Delta \overline{y}) ) : \partial _{t}\nabla \Delta \hat{y} (T - t) \textrm{d}x \textrm{d}t \\&\quad = \int _I\int _\Omega \big ( \partial _F W(\nabla \overline{y}, \underline{\theta }) + \partial _{\dot{F}} R(\nabla \underline{y}, \partial _t \nabla \hat{y}, \underline{\theta }) \big ) : \partial _{t} \nabla \Delta \hat{y} (T - t) \textrm{d}x \textrm{d}t \\&\phantom {\quad =}\quad - \int _I\int _\Omega \overline{f} \cdot \partial _{t} \Delta \hat{y} (T - t) \textrm{d}x \textrm{d}t - \frac{\rho }{h} \int _0^T \int _\Omega (\partial _t \nabla \hat{y}(t) - \partial _t \nabla \hat{y}(t - h)) : \\&\qquad \partial _{t} \nabla \hat{y}(t) (T - t) \textrm{d}x \textrm{d}t. \end{aligned} \end{aligned}$$

We denote the last term on the right-hand side of (3.70) (without negative sign) by $\Pi _T$. An expansion yields3.71Combining (3.70) and (3.71) and applying Hölder inequality, we deduce that

$$\begin{aligned}&\varepsilon \Vert \partial _{t} \Delta ^{2}\hat{y} \Vert _{L^{2}_T(I; L^2(\Omega ))}^{2} \le \Vert \nabla (DH(\Delta \overline{y})) \Vert _{L^{2}_T (I; L^{p'}(\Omega ))} \Vert \partial _{t} \nabla \Delta \hat{y} \Vert _{L^{2}_T(I; L^p(\Omega ))} \\&\quad \phantom {\le } + \sqrt{T} \Vert ( \partial _F W(\nabla \overline{y}, \underline{\theta }) + \partial _{\dot{F}} R(\nabla \underline{y}, \partial _t \nabla \hat{y}, \underline{\theta }) ) \Vert _{L^{2}(I \times \Omega )} \Vert \partial _{t} \nabla \Delta \hat{y} \Vert _{L^{2}_T(I; L^2(\Omega ))} \\&\quad \phantom {\le } + \sqrt{T} \Vert f \Vert _{L^{2} ( I \times \Omega ) } \Vert \partial _{t} \Delta \hat{y}\Vert _{ L^{2}_T(I; L^2(\Omega ))} + \frac{\rho T }{2}\Vert y'_{0, \varepsilon } \Vert _{H^{1}(\Omega )}^{2}\,. \end{aligned}$$

Then, by (3.67)–(3.69), Hölder’s inequality, and Poincaré’s inequality along with $\Delta \partial _t \hat{y} = 0$ on $I \times \partial \Omega$ we derive

3.72 $$\begin{aligned}&\varepsilon \Vert \partial _{t} \Delta ^{2}\hat{y} \Vert _{L^{2}_T(I;L^2(\Omega ))}^{2} \le \ C ( 1 + \sqrt{\varepsilon } \Vert y_{0, \varepsilon }\Vert _{H^{4} (\Omega )} + \Vert y'_{0, \varepsilon }\Vert _{H^{1}(\Omega )}) \Vert \partial _{t} \nabla \Delta \hat{y}\Vert _{L^{2}_T(I; L^p(\Omega ))} \nonumber \\&\quad + C \Vert y'_{0, \varepsilon }\Vert _{H^{1}(\Omega )}^{2}. \end{aligned}$$

By the Gagliardo-Nirenberg interpolation inequality with $$\theta = \frac{p-2}{2p}d$$ ($$\theta \in (0,1)$$ as $$p \in (3,6)$$ for $$d=3$$), see e.g. [42, Theorem 1.24]) for $$r= p $$, $$\beta = 0$$, $$k=1$$, and $$p = q = 2$$, we get

$$\begin{aligned} \Vert \partial _{t} \nabla \Delta \hat{y}\Vert _{L^{2}_T(I; L^p(\Omega ))} \le \Vert \partial _{t} \nabla \Delta \hat{y}\Vert _{L^{2}_T(I; L^2(\Omega ))} + \Vert \partial _{t} \nabla \Delta \hat{y}\Vert _{L^{2}_T(I; L^2(\Omega ))}^{1-\theta } \Vert \partial _{t} \nabla ^2 \Delta \hat{y}\Vert _{L^{2}_T(I; L^2(\Omega ))}^\theta . \end{aligned}$$

By elliptic regularity for the operator $\Delta$ and the fact that $\Delta \partial _t \hat{y} = 0$ on $I \times \partial \Omega$ we get $\Vert \partial _{t} \nabla ^2 \Delta \hat{y}\Vert _{L^{2}_T(I; L^2(\Omega ))} \le C \Vert \partial _{t} \Delta ^{2}\hat{y}\Vert _{L^{2}_T(I; L^2(\Omega ))}$. Then, using the weighted Young’s inequality for $\lambda >0$ and exponent $\frac{2}{\theta }$ we get

$$\begin{aligned}  &   \Vert \partial _{t} \nabla \Delta \hat{y}\Vert _{L^{2}_T(I; L^p(\Omega ))} \le \Vert \partial _{t} \nabla \Delta \hat{y}\Vert _{L^{2}_T(I; L^2(\Omega ))} + C(\varepsilon \lambda )^{-\frac{\theta }{2-\theta }} \Vert \partial _{t} \nabla \Delta \hat{y}\Vert _{L^{2}_T(I; L^2(\Omega ))}^{\frac{2(1-\theta )}{2-\theta }} \\  &   + C\varepsilon \lambda \Vert \partial _{t} \Delta ^{2}\hat{y}\Vert _{L^{2}_T(I; L^2(\Omega ))}^2. \end{aligned}$$

The bound $\sqrt{\varepsilon } \Vert \partial _{t} \hat{y} \Vert _{L^2( I; H^3(\Omega ))} \le C$ given by (3.37b) and the fact that $\frac{1}{2-\theta } = \varrho \in (\frac{1}{2},1)$ (see (3.43)) yield

$$\begin{aligned}  &   \Vert \partial _{t} \nabla \Delta \hat{y}\Vert _{L^{2}_T(I; L^p(\Omega ))} \le C\varepsilon ^{-1/2} + C_\lambda \varepsilon ^{-\frac{\theta }{2-\theta }} \varepsilon ^{-\frac{1-\theta }{2-\theta }} + C\varepsilon \lambda \Vert \partial _{t} \Delta ^{2}\hat{y}\Vert _{L^{2}_T(I; L^2(\Omega ))}^2 \\  &   \le C_\lambda \varepsilon ^{-\varrho } + C\varepsilon \lambda \Vert \partial _{t} \Delta ^{2}\hat{y}\Vert _{L^{2}_T(I; L^2(\Omega ))}^2. \end{aligned}$$

Multiplying inequality (3.72) by $\varepsilon ^{\varrho }$ and choosing $\lambda$ sufficiently small we get

$$\begin{aligned} \varepsilon ^{1+\varrho } \Vert \partial _{t} \Delta ^{2}\hat{y} \Vert _{L^{2}_T(I;L^2(\Omega ))}^{2}&\le \ C ( 1 + \sqrt{\varepsilon } \Vert y_{0, \varepsilon }\Vert _{H^{4} (\Omega )} + \Vert y'_{0, \varepsilon }\Vert _{H^{1}(\Omega )}) + C \varepsilon ^\varrho \Vert y'_{0, \varepsilon }\Vert _{H^{1}(\Omega )}^{2} \end{aligned}$$

for some constant $$C>0$$ independent of $$\varepsilon \in (0, 1)$$ and of $$ h>0$$. This concludes the proof of (3.47) by using (3.42) and an elliptic regularity estimate. $$\square $$

### Proof of Theorem 3.1

In this subsection, we separately discuss the limiting passage $\tau \rightarrow 0$ for the mechanical and the heat-transfer equation, leading to (3.1a) and (3.1b), respectively.

#### Lemma 3.14

(Convergence of the mechanical equation) Let $(y_h,\theta _h)$ be as in Proposition 3.10. Then, for any test function $z \in C^\infty ( I \times \overline{\Omega }; \mathbb {R}^d)$ satisfying $z = 0$ on $I \times \partial \Omega$ we have that (3.1a) holds.

#### Proof

By (3.38a) we have that $\partial _t \hat{y}_{\tau } \rightharpoonup y_h$ weakly in $L^2(I_h; H^3(\Omega ;\mathbb {R}^d))$ and by (3.38b) it holds that $\hat{y}_{\tau }(t), \overline{y}_{\tau }(t)$, $\underline{y}_{\tau }(t) \rightarrow y_h$ strongly in $W^{2, p}(\Omega ; \mathbb {R}^{d})$ for a.e. $t \in I$. Moreover, $(\hat{y}_{\tau })_\tau$, $(\overline{y}_{\tau })_\tau$, and $(\underline{y}_{\tau })_\tau$ are bounded in $L^\infty (I; W^{2, p}(\Omega ; \mathbb {R}^{d}))$ independently of $\tau$ and *h*. Hence, we deduce (3.1a) by testing (3.6) with *z*, summing over *k*, and passing to the limit as $\tau \rightarrow 0$ using the generalized dominated convergence theorem. Here, we crucially use that $\partial _{\dot{F}} R$ is linear in the second entry, cf. (D.1). $\square$

Before we concern ourselves with the limiting passage in the heat-transfer equation, we establish a mechanical energy balance for $(y_h,\theta _h)$.

#### Lemma 3.15

(Mechanical energy balance) Let $(y_h,\theta _h)$ be as in Proposition 3.10 with $y_h(0, \cdot ) = y_{0, \varepsilon }$ satisfying (3.1a). Then, for any $t \in I$ we have the mechanical energy balance3.73

#### Proof

By the regularity $y_h \in H^1(I_h;H^3(\Omega ;\mathbb {R}^d))$, using the chain rule for $\Lambda$-convex functionals (see [37, Proposition 3.6] and Lemma 3.8), we first observe that the mechanical energy defined in (2.6) satisfies that $t \mapsto \mathcal {M}(y_h(t))$ lies in $W^{1,1}(I)$ and that

3.74 $$\begin{aligned} \frac{\textrm{d}}{\textrm{d}t } \mathcal {M} (y_{h}) = \int _{\Omega } {D H}( \Delta y_{h}) : \partial _{t} \Delta y_{h} \textrm{d}x + \int _{\Omega } \partial _{F} W^{\textrm{el}}( \nabla y_{h} ) \cdot \partial _{t} \nabla y_{h} \textrm{d}x \quad \text {for a.e. }t \in I. \end{aligned}$$

We test the equation (3.1a) with $z :=\partial _{t} y_{h} 1\!\!1_{[0,t]}$, and obtain by (2.11)

$$\begin{aligned}&\int _0^t \int _\Omega \big ( \partial _F W^{\textrm{el}}(\nabla y_h) + \partial _{F} W^\textrm{cpl} (\nabla y_{h}, \theta _{h}) \big ) : \partial _{t}\nabla y_{h} \textrm{d}x \textrm{d}s + \int _0^t \int _\Omega D H( \Delta y_h) \cdot \partial _{t} \Delta y_{h} \textrm{d}x \textrm{d}s \\&- \int _0^t \int _\Omega f \cdot \partial _{t} y_{h} \textrm{d}x \textrm{d}s +\frac{\rho }{h} \int _0^t \int _\Omega \big ( \partial _t y_h(s) - \partial _t y_h(s - h) \big ) \cdot \partial _{t} y_{h} (s) \textrm{d}x \textrm{d}s \\&\qquad = -\int _0^t \int _\Omega 2 R(\nabla y_h, \partial _t \nabla y_h, \theta _h) \textrm{d}x \textrm{d}s - \varepsilon \int _{0}^{t} \int _{\Omega } |\partial _t \nabla \Delta y_{h}|^2 \textrm{d}x \textrm{d}s . \end{aligned}$$

Applying the chain rule (3.74) we find

3.75 $$\begin{aligned}&\mathcal {M} (y_h(t)) - \mathcal {M}(y_{0,\varepsilon }) + \int _0^t \int _\Omega \partial _{F} W^\textrm{cpl} (\nabla y_{h}, \theta _{h}) : \partial _{t}\nabla y_{h} \textrm{d}x \textrm{d}s - \int _0^t \int _\Omega f \cdot \partial _{t} y_{h} \textrm{d}x \textrm{d}s \nonumber \\&\quad = -\int _0^t \int _\Omega \Big ( 2 R(\nabla y_h, \partial _t \nabla y_h, \theta _h) + \varepsilon |\partial _t \nabla \Delta y_{h}|^2 \Big ) \textrm{d}x \textrm{d}s \nonumber \\&- \frac{\rho }{h} \int _0^t \int _\Omega \big ( \partial _t y_h(s) - \partial _t y_h(s - h) \big ) \cdot \partial _{t} y_{h} (s) \textrm{d}x \textrm{d}s. \end{aligned}$$

Denoting the last term on the right-hand side by $\Pi$ (without negative sign), and expanding it as in (3.71), we derivePlugging this into (3.75) and using (2.12) as well as $\partial _t y_h(s) = y_{0,\varepsilon }'$ for $s \in (-h,0)$, the proof of the mechanical energy balance is concluded. $\square$

#### Lemma 3.16

(Convergence of the heat-transfer equation) Let $(y_h,\theta _h)$ be as in Proposition 3.10. Then, for any test function $\varphi \in C^\infty (I \times \overline{\Omega })$ equation (3.1b) is satisfied.

#### Proof

The essential point is to show strong convergence of the strain rates, namely

3.76 $$\begin{aligned} \nabla \partial _t \hat{y}_\tau \rightarrow \nabla \partial _t y_h \text { and } \nabla \Delta \partial _t \hat{y}_\tau \rightarrow \nabla \Delta \partial _t y_h \quad \text {strongly in } L^2(I; L^2(\Omega ; \mathbb {R}^{d\times d}) ). \end{aligned}$$

Once this is achieved, using the convergences in (3.38a)–(3.38d), we can almost verbatim follow the proof of [2, Proposition 4.6], recalling that the scheme for the heat-transfer equation differs from the one in [2] only by the regularizing term $\varepsilon |\partial _t \nabla \Delta \hat{y}_\tau |^{2} \wedge \tau ^{-1}$, see (3.9) and [2, Equation (3.11)]. The only difference is that for the term

$$\begin{aligned} \int _\Omega \partial _t \hat{w}_\tau \varphi \textrm{d}x \end{aligned}$$

in (3.9) we do not perform an integration by parts in time, but directly pass to the limit using that $\partial _t\hat{w}_\tau \rightharpoonup \partial _t w_h$ in $L^2( I; (H^1(\Omega ))^*)$ by Corollary 3.13. This gives (3.1b).

The argument for showing (3.76) is along the lines of [2, Lemma 4.5] or [37, Proposition 5.1], and relies on passing to the limit in the mechanical energy balance. We briefly sketch the argument. In view of the notation in (3.36), we can write the discrete mechanical energy estimate in Lemma 3.9 asEmploying (3.38a)–(3.38d), standard lower semicontinuity arguments (see [2, Equation (4.15)] and also [25, Theorem 7.5] for a general result) imply,

3.77a $$\begin{aligned} \liminf _{\tau \rightarrow 0} \mathcal {M}(\overline{y}_\tau (T))&\ge \mathcal {M}(y_h(T)), \end{aligned}$$

3.77b $$\begin{aligned} \liminf _{\tau \rightarrow 0} \int _0^T \int _\Omega \xi (\nabla \underline{y}_\tau , \partial _t \nabla \hat{y}_\tau , \underline{\theta }_\tau ) \textrm{d}x \textrm{d}t&\ge \int _0^T \int _\Omega \xi (\nabla y_h, \partial _t \nabla y_h, \theta _h) \textrm{d}x \textrm{d}t, \end{aligned}$$

3.77c $$\begin{aligned} \liminf _{\tau \rightarrow 0} \int _0^T \int _\Omega \varepsilon |\partial _t \nabla \Delta \hat{y}_{\tau }|^2 \textrm{d}x \textrm{d}t&\ge \int _0^T \int _\Omega \varepsilon |\partial _t \nabla \Delta {y}_h|^2 \textrm{d}x \textrm{d}t, \end{aligned}$$

3.77d

3.77e $$\begin{aligned} \liminf _{\tau \rightarrow 0} \frac{\rho }{2h} \int _0^T \Vert \partial _t \hat{y}_\tau (t) - \partial _t \hat{y}_\tau (t-h)\Vert _{L^2(\Omega )}^2 \, \textrm{d}t&\ge \frac{\rho }{2h}\int _0^T \Vert \partial _t y_h(t) - \partial _t y_h(t-h) \Vert ^2_{L^2(\Omega )} \textrm{d}t. \end{aligned}$$

Since $\int _0^T ( \overline{f}_\tau (t), \partial _t \hat{y}_\tau (t) )_2 \, \textrm{d}t \rightarrow \int _0^T \int _\Omega f \cdot \partial _{t} y_{h} \textrm{d}x \textrm{d}t$, as well as $\int _0^T \int _\Omega \partial _F W^{\textrm{cpl}}(\underline{y}_\tau , \underline{\theta }_\tau ) : \partial _t \nabla \hat{y}_\tau \textrm{d}x \, \textrm{d}t$ converges to $\int _0^T \int _\Omega \partial _{F} W^\textrm{cpl} (\nabla y_{h}, \theta _{h}) : \partial _{t}\nabla y_{h} \textrm{d}x \textrm{d}t$ by (3.38a)–(3.38d), and $\tau V_{T/\tau } \rightarrow 0$ as $\tau \rightarrow 0$ by (3.37b), using the mechanical energy balance (3.73), we find that all estimates (3.77a)–(3.77e) are actually equalities, see [2, Equation (4.15)] for details on this argument. In particular, the convergence in (3.77b) implies the first part of (3.76), by repeating the arguments in [2, Equation (4.16)ff.]. Eventually, the convergence in (3.77c) provides the second part of (3.76). $\square$

#### Proof of Theorem 3.1

The statement follows by collecting the regularity for $y_h$, $\theta _h$, and $w_h$ given in Proposition 3.10 and Corollary 3.13, and the identification of the limiting equations in Lemmas 3.14 and 3.16. $\square$

## Vanishing time-delay

We recall that for each $T, \, h, \, \varepsilon > 0$, Theorem 3.1 guarantees the existence of $(y_h, \theta _h)$ such that $y_h \in L^\infty ( I; \mathcal {Y}^\textrm{reg}_{\textbf{id}}) \cap H^1(I_h; H^3(\Omega ; \mathbb {R}^d))$, $\theta _h \in L^2(I; H^1_+(\Omega ))$, and such that equations (3.1a)–(3.1b) hold. We also recall that $y_h(t) = y_{0,\varepsilon } + t y_{0,\varepsilon }'$ for $t \in [-h, 0]$. The goal of this section is to pass to the limit $h \rightarrow 0$ in (3.1a)–(3.1b). We start by a compactness result.

### Lemma 4.1

(Compactness) There exist $y_\varepsilon \in L^{\infty }(I; \mathcal {Y}^\textrm{reg}_{\textbf{id}}) \cap H^1(I; H^3(\Omega ; \mathbb {R}^d))$ with $y_\varepsilon (0) = y_{0,\varepsilon }$, and $\theta _\varepsilon \in L^2(I; H^1_+(\Omega ))$, as well as $w_\varepsilon :=W^{\textrm{in}}(\nabla y_\varepsilon , \theta _\varepsilon ) \in L^2(I; H^1(\Omega )) \cap H^1(I; (H^1(\Omega ))^*)$ with $w_\varepsilon (0) = w_{0,\varepsilon }$ such that, up to selecting a subsequence (not relabeled), it holds that

4.1a $$\begin{aligned} y_h&\rightharpoonup y_\varepsilon \quad \text {weakly in } H^1(I; H^3(\Omega ; \mathbb {R}^d)), \end{aligned}$$

4.1b $$\begin{aligned} y_h&\rightarrow y_\varepsilon \quad \text {strongly in } C (I; W^{1, \infty }(\Omega ; \mathbb {R}^{d})) \ \ \text { and strongly in } C(I; W^{2, p}(\Omega ; \mathbb {R}^{d})), \end{aligned}$$

4.1c $$\begin{aligned} \theta _h&\rightarrow \theta _\varepsilon \quad \text { and } \quad w_h \rightarrow w_\varepsilon \text { strongly in } L^s(I \times \Omega ) \text { for any } s \in [1, \tfrac{d+2}{d}), \end{aligned}$$

4.1d $$\begin{aligned} \theta _h&\rightharpoonup \theta _\varepsilon \quad \text { and } \quad w_h \rightharpoonup w_\varepsilon \text { weakly in } L^2(I; H^1(\Omega )) , \end{aligned}$$

4.1e $$\begin{aligned} w_h&\rightharpoonup w_\varepsilon \text { weakly in } H^1(I; (H^1(\Omega ))^*) . \end{aligned}$$

### Proof

As the a priori bounds derived in Proposition 3.10 and Corollary 3.13 are independent of *h*, we see by the lower semicontinuity of norms that the same bounds hold true for $(y_h, \theta _h)$ in place of $(y_\tau , \theta _\tau )$. Then, (4.1a)–(4.1c) can be obtained exactly as in the proof of Proposition 3.10, and (4.1d) –(4.1e) follow from the bounds in Corollary 3.13 by weak compactness and the Aubin-Lions’ lemma, see also [37, Proposition 5.1]. $\square$

We collect a priori bounds for the limits $(y_{\varepsilon }, \theta _{\varepsilon })$ which directly follow from Proposition 3.10, Lemma 3.12, Lemma 4.1, and lower semicontinuity: for each $q \in [1, \frac{d + 2}{d})$ and $r \in [1, \frac{d+2}{d+1})$ we can find constants *C*, $C_q$, and $C_r$ independent of $\varepsilon$ such that

4.2a $$\begin{aligned} \Vert y_{\varepsilon } \Vert _{L^\infty (I; W^{2, p}(\Omega ))} + \Vert \det (\nabla y_{\varepsilon })^{-1} \Vert _{L^\infty (I \times \Omega )}&\le C , \end{aligned}$$

4.2b $$\begin{aligned} \Vert y_{\varepsilon } \Vert _{H^1(I \times \Omega )} + \sqrt{\varepsilon } \Vert \partial _{t} {y}_{\varepsilon }\Vert _{L^{2}(I;H^3(\Omega ))}&\le C , \end{aligned}$$

4.2c $$\begin{aligned} \Vert \theta _{\varepsilon } \Vert _{L^\infty (I; L^1(\Omega ))} + \Vert w_{\varepsilon } \Vert _{L^\infty (I; L^1(\Omega ))}&\le C \end{aligned}$$

4.2d $$\begin{aligned} \Vert \theta _{\varepsilon } \Vert _{L^q(I \times \Omega )} + \Vert w_{\varepsilon } \Vert _{L^q(I \times \Omega )} \le C_q , \end{aligned}$$

4.2e $$\begin{aligned} \Vert \nabla \theta _{\varepsilon } \Vert _{L^r(I \times \Omega )} + \Vert \nabla w_{\varepsilon } \Vert _{L^r(I \times \Omega )} \le C_r. \end{aligned}$$

Furthermore, there exist $\varrho \in (\frac{1}{2},1)$ and $C>0$ independently of $\varepsilon$ with

4.3a $$\begin{aligned}&\Vert \Delta y_{\varepsilon } \Vert _{L^{2} (I; H^{1}(\Omega ))} + \Vert |\Delta y_{\varepsilon }|^{\frac{p-2}{2}} \nabla (\Delta y_{\varepsilon }) \Vert _{L^{2} (I \times \Omega )} \le C \big ( 1 + \sqrt{\varepsilon } \Vert y_{0,\varepsilon }\Vert _{H^{4}(\Omega )} + \Vert y'_{0, \varepsilon }\Vert _{H^{1}( \Omega )} \big ) , \end{aligned}$$

4.3b $$\begin{aligned}&\Vert \nabla {D H}(\Delta y_\varepsilon )\Vert _{L^2(I;L^{p'}(\Omega ))} \le C \big ( 1 + \sqrt{\varepsilon } \Vert y_{0, \varepsilon }\Vert _{H^{4}(\Omega )} + \Vert y'_{0, \varepsilon }\Vert _{H^{1}(\Omega )} \big ) \,, \end{aligned}$$

4.3c $$\begin{aligned}&\Vert \Delta \partial _{t} y_{\varepsilon } \Vert _{L^{2}(I; H^2(\Omega ))} \le C \varepsilon ^{-\frac{1+\varrho }{2}} \big ( 1 + \root 4 \of {\varepsilon } \Vert y_{0, \varepsilon }\Vert _{H^{4}(\Omega )} + \Vert y'_{0, \varepsilon }\Vert _{H^{1}(\Omega )} \big ). \end{aligned}$$

Moreover, we have

4.4a $$\begin{aligned} \Delta y_{\varepsilon }(t)&= \partial _{\nu } \Delta y_{\varepsilon }(t) = 0 \qquad \text {on }\partial \Omega \text { for a.e. }t \in I, \end{aligned}$$

4.4b $$\begin{aligned} \Delta \partial _{t} y_{\varepsilon } (t)&= \partial _{\nu } \Delta \partial _{t} y_{\varepsilon }(t) = 0 \qquad \text {on }\partial \Omega \text { for a.e. }t \in I. \end{aligned}$$

In particular, (4.4b) follows from the boundary conditions deduced in Proposition 3.11, the estimates obtained in Lemma 3.12 on $(\partial _{t} y_{h})_h$, and a compactness argument, while (4.4a) is a consequence of (4.4b) and the fact that $y_{0,\varepsilon } \in \mathcal {Y}^\textrm{reg}_{\textbf{id}}$, see (2.20).

The rest of the section is devoted to the proof of Theorems 2.2 and 2.3. In particular, we show that $(y_\varepsilon ,\theta _\varepsilon )$ is a solution to the regularized system (2.21)–(2.22). As the second bound in (3.37b) transfers uniformly to $\partial _t y_h$, there exists $y_{T,\varepsilon }' \in L^2(\Omega ;\mathbb {R}^d)$ such that, as $h \rightarrow 0$,4.5

### Proposition 4.2

(Auxiliary mechanical equation) Let $(y_\varepsilon , \theta _\varepsilon )$ be as in Lemma 4.1. Then, $(y_\varepsilon , \theta _\varepsilon )$ satisfies

4.6 $$\begin{aligned} \begin{aligned} 0=&\int _I \int _{\Omega } \partial _{F} W(\nabla y_\varepsilon , \theta _\varepsilon ) : \nabla z \textrm{d}x \textrm{d}t + \int _I \int _{\Omega } {D H} ( \Delta y_\varepsilon ) \cdot \Delta z \textrm{d}x \textrm{d}t \\&+ \varepsilon \int _I \int _{\Omega } \partial _t \nabla \Delta y_\varepsilon : \nabla \Delta z \textrm{d}x \textrm{d}t \\&\quad - \int _I \int _{\Omega } f \cdot z \textrm{d}x \textrm{d}t + \int _I \int _{\Omega } \partial _{\dot{F}} R(\nabla y_\varepsilon , \partial _t \nabla y_\varepsilon , \theta _\varepsilon ) : \nabla z \textrm{d}x \textrm{d}t \\&- \rho \int _I \int _{\Omega } \partial _t y_\varepsilon \cdot \partial _t z \textrm{d}x \textrm{d}t \\&\qquad + \rho \int _{\Omega } \big (y'_{T,\varepsilon } \cdot z(T) - y'_{0,\varepsilon } \cdot z(0)\big ) \textrm{d}x, \end{aligned} \end{aligned}$$

for every $z \in C^{\infty } ( I \times \overline{\Omega }; \mathbb {R}^{d})$ with $z = 0$ on $I \times \partial \Omega$.

### Proof

Notice that by a change of variables we can rewrite (3.1a) for every $z \in C^\infty (I \times \overline{\Omega }; \mathbb {R}^{d})$ with $z = 0$ on $I \times \partial \Omega$ asBy the smoothness of *z* and (4.5) it directly follows thatMoreover, the convergence in (4.1a) and the smoothness of *z* show by weak-strong convergence

$$\begin{aligned} \int _0^{T-h} \int _\Omega \partial _t y_h(t) \cdot \frac{z(t + h) - z(t)}{h} \textrm{d}x \textrm{d}t \rightarrow \int _0^T \int _\Omega \partial _t y_\varepsilon \cdot \partial _t z \textrm{d}x \textrm{d}t \qquad \text {as } h \rightarrow 0. \end{aligned}$$

By (4.1b), assumption (H.3), and the generalized dominated convergence theorem, we derive that

$$\begin{aligned} \int _I\int _\Omega D H (\Delta y_h) \cdot \Delta z \textrm{d}x \textrm{d}t \rightarrow \int _I\int _\Omega D H (\Delta y_\varepsilon ) \cdot \Delta z \textrm{d}x \textrm{d}t \qquad \text {as } h \rightarrow 0. \end{aligned}$$

Finally, by (4.1a)–(4.1c) the convergence of all remaining terms follows, leading to the desired equation (4.6). $\square$

### Proposition 4.3

(Mechanical equation) Let $(y_\varepsilon , \theta _\varepsilon )$ be as in Lemma 4.1. Then, $(y_\varepsilon , \theta _\varepsilon )$ satisfies (2.21). Moreover, it holds that $\partial _{tt}^2 y_\varepsilon \in L^2(I; (H^3(\Omega ;\mathbb {R}^d) \cap H^1_0(\Omega ;\mathbb {R}^d))^*)$ with $\Vert \partial _{tt}^2 y_\varepsilon \Vert _{L^2(I; (H^3(\Omega ) \cap H^1_0(\Omega ))^*)} \le C$ for a constant $C>0$ independent of $\varepsilon$. In particular, $\partial _t y_\varepsilon \in C(I;L^2(\Omega ;\mathbb {R}^d))$ with $\partial _t y_\varepsilon (0) = y'_{0,\varepsilon }$ and $\partial _t y_\varepsilon (T) = y'_{T,\varepsilon }$.

### Proof

In view of (4.2a)–(4.2e), all time integrals in (4.6) except for $\rho \int _I \int _{\Omega } \partial _t y_\varepsilon \cdot \partial _t z \textrm{d}x \textrm{d}t$ lie in $L^2(I;(H^3(\Omega ) \cap H^1_0(\Omega ))^*)$, where for the nonlinear term $\int _I \int _{\Omega } {D H} ( \Delta y) \cdot \Delta z \textrm{d}x \textrm{d}t$ we particularly use (H.3), (4.2a), and the fact that $\Delta z \in L^2(I; L^p(\Omega ))$ for each $z \in L^2(I;H^3(\Omega ) \cap H^1_0(\Omega ))$ ($p \in (3,6)$ for $d=3$). More precisely, the corresponding operator norms are uniformly bounded independently of $\varepsilon$. Then, by definition of weak derivatives we get that $\partial _{tt}^2 y_\varepsilon \in L^2(I; (H^3(\Omega ;\mathbb {R}^d) \cap H^1_0(\Omega ;\mathbb {R}^d))^*)$ exists and is bounded independently of $\varepsilon$. Moreover, an integration by parts in time shows (2.21) for $z \in C^{\infty } ( I \times \overline{\Omega }; \mathbb {R}^{d})$ with $z = 0$ on $I \times \partial \Omega$ and $z(0)=z(T) = 0$. Then, by a density argument we observe that the assumption $z(0)=z(T)=0$ can be dropped. Eventually, $\partial _t y_\varepsilon \in C(I;L^2(\Omega ;\mathbb {R}^d))$ follows from [42, Lemma 7.3], and $\partial _t y_\varepsilon (0) = y'_{0,\varepsilon }$ as well as $\partial _t y_\varepsilon (T) = y'_{T,\varepsilon }$ follow by integration by parts in time of (2.21) for general $z \in C^{\infty } ( I \times \overline{\Omega }; \mathbb {R}^{d})$ and a comparison with (4.6). $\square$

We proceed with a mechanical energy balance which is the analog to the one in (3.73). We note that the balance can be formulated for each $t \in I$ since $y_\varepsilon \in C(I; W^{2, p}(\Omega ; \mathbb {R}^{d}))$ and $\partial _t y_\varepsilon \in C(I;L^2(\Omega ;\mathbb {R}^d))$.

### Lemma 4.4

(Mechanical energy balance) Let $(y_\varepsilon , \theta _\varepsilon )$ be as in Lemma 4.1 satisfying (2.21). Then, for any $t \in I$ we have the mechanical energy balance (2.23).

### Proof

Since $\partial ^{2}_{tt} y_\varepsilon$ lies in $L^{2} (I; ( H^{3} (\Omega ; \mathbb {R}^{d}) \cap H^{1}_{0} (\Omega ; \mathbb {R}^{d}))^{*})$, see Proposition 4.3, by an approximation argument we can test (2.21) with $z = \partial _{t} y_\varepsilon 1\!\!1_{[0,t]}$ and obtain

4.7 $$\begin{aligned}&\int _0^t \int _\Omega \Big ( \partial _F W^{\textrm{el}}(\nabla y_\varepsilon ) + \partial _{F} W^\textrm{cpl} (\nabla y_\varepsilon , \theta _\varepsilon ) \Big ) : \partial _{t} \nabla y_\varepsilon \textrm{d}x \textrm{d}t \nonumber \\&\quad + \int _0^t \int _\Omega D H( \Delta y_\varepsilon ) : \partial _{t}\Delta y_\varepsilon \textrm{d}x \textrm{d}s \nonumber \\&\quad \quad = - \int _0^t 2 \mathcal {R}_\varepsilon ( y_\varepsilon , \partial _t y_\varepsilon , \theta _\varepsilon ) \textrm{d}s - \rho \int _0^t \langle \partial ^2_{tt} y_\varepsilon , \partial _t y_\varepsilon \rangle \textrm{d}s + \int _0^t \int _\Omega f \cdot \partial _{t} y_\varepsilon \textrm{d}x \textrm{d}s . \end{aligned}$$

Using the chain rule we find

$$\begin{aligned} \rho \int _0^t \langle \partial ^2_{tt} y_\varepsilon , \partial _t y_\varepsilon \rangle \textrm{d}s&= \frac{\rho }{2} \int _0^t \int _{\Omega } \frac{\textrm{d}}{\textrm{d}t } |\partial _t y_\varepsilon |^{2} \, \textrm{d}x \textrm{d}s = \frac{\rho }{2} \Vert \partial _t y_h(t) \Vert ^2_{L^2(\Omega )} - \frac{\rho }{2} \Vert y_{0,\varepsilon }' \Vert ^2_{L^2(\Omega )} . \end{aligned}$$

Combining this with the chain rule in (3.74) (for $y_\varepsilon$ in place of $y_h$) and plugging into (4.7), the proof is concluded, using again (2.11)–(2.12). $\square$

### Proposition 4.5

(Heat-transfer equation) Let $(y_\varepsilon , \theta _\varepsilon )$ be as in Lemma 4.1. Then, $(y_\varepsilon , \theta _\varepsilon )$ satisfies (2.22).

### Proof

As in the proof of Lemma 3.16, the essential point is to show strong convergence of the strain rates, namely

4.8 $$\begin{aligned} \nabla \partial _t y_h \rightarrow \nabla \partial _t y_\varepsilon \text { and } \nabla \Delta \partial _t y_h \rightarrow \nabla \Delta \partial _t y_\varepsilon \quad \text {strongly in } L^2(I; L^2(\Omega ; \mathbb {R}^{d\times d}) ), \end{aligned}$$

as then one can pass to the limit in each term by using the convergences in (4.1a)–(4.1e). Rearranging the terms in (3.73), dropping one nonnegative term, and passing to the liminf as $h \rightarrow 0$, by the convergences in Lemma 4.1 we get4.9By the convergences in Lemma 4.1 and standard lower semicontinuity arguments we get4.10For the first two estimates we also refer to [2, Equation (4.15)] and the last one follows from (4.5) and Proposition 4.3. Combining (4.9)–(4.10) with the mechanical energy balance in Lemma 4.4, we conclude that all inequalities in (4.10) are actually equalities. Then, (4.8) follows exactly as in the final argument of the proof of Lemma 3.16. $\square$

### Proof (Proofs of Theorems 2.2 and 2.3)

The weak formulation, the regularity properties of $(y_\varepsilon ,\theta _\varepsilon ,w_\varepsilon )$, and the initial conditions for $y_\varepsilon ,\partial _t y_\varepsilon , w_\varepsilon$ follow from Lemma 4.1, Proposition 4.3, and Proposition 4.5. The mechanical energy balance is given in Lemma 4.4.

Concerning (2.24), we observe that by density we can test (2.22) with functions $\varphi \in W^{1,1}(I)$ which are independent of the space variable *x* and satisfy $\varphi (T) = 0$. For $t \in (0,T)$ fixed, we define the test function $\varphi$ with $\varphi \equiv 1$ on $(0,t-\delta )$, $\varphi \equiv 0$ on $(t+\delta ,T)$, and $\varphi ' \equiv -\frac{1}{2\delta }$ on $(t-\delta , t + \delta )$. Then, by an integration by parts in time for the term $\int _I \langle \partial _t w_\varepsilon , \varphi \rangle \textrm{d}t$, in the limit $\delta \rightarrow 0$, (2.22) yields

$$\begin{aligned} 0 =&\int _0^t -\Big ( \xi (\nabla y_\varepsilon , \partial _t \nabla y_\varepsilon , \theta _\varepsilon ) + \partial _F W^{\textrm{cpl}}(\nabla y_\varepsilon , \theta _\varepsilon ) : \partial _t \nabla y_\varepsilon + \varepsilon |\partial _t \nabla \Delta y_\varepsilon |^2 \Big ) \textrm{d}x \textrm{d}t \\&\quad - \kappa \int _0^t \int _{\partial \Omega } (\theta _\flat - \theta _\varepsilon ) \textrm{d}\mathcal {H}^{d-1} \textrm{d}t + \lim _{\delta \rightarrow 0} \frac{1}{2\delta }\int _{t-\delta }^{t+\delta } \int _\Omega w_\varepsilon \textrm{d}x \textrm{d}t - \int _\Omega w_{0,\varepsilon } \textrm{d}x. \end{aligned}$$

As $w_\varepsilon \in C(I;L^2 (\Omega ))$ by Lemma 4.1 and [42, Lemma 7.3], we find $\lim _{\delta \rightarrow 0} \frac{1}{2\delta }\int _{t-\delta }^{t+\delta } \int _\Omega w_\varepsilon \textrm{d}x$ $\textrm{d}s = \int _\Omega w_\varepsilon (t,x) \textrm{d}x$, which concludes the proof of (2.24). Eventually, (2.25) follows by summation of (2.23) and (2.24). $\square$

## Vanishing regularization: Proof of Theorem 2.5

This section is devoted to the analysis of the limit of the regularized thermo-elastodynamic system (2.21)–(2.22) as $\varepsilon \rightarrow 0$. This will show existence of solutions to the system (2.26)–(2.27), i.e., Theorem 2.5. First, given initial data $y_{0} \in \mathcal {Y}_{\textbf{id}}$ and $y'_{0} \in H^{1}_{0} (\Omega ; \mathbb {R}^{d})$, we consider suitable regularizations $y_{0, \varepsilon } \in \mathcal {Y}^\textrm{reg}_{\textbf{id}}$ (see (2.20)) and $y'_{0, \varepsilon } \in H^{3} (\Omega ; \mathbb {R}^{d})$ such that

5.1 $$\begin{aligned}&y_{0, \varepsilon } \rightarrow y_{0} \text { in }W^{2, p}(\Omega ; \mathbb {R}^{d}) \quad and \quad y'_{0, \varepsilon } \rightarrow y'_{0} \text { in }H^{1}(\Omega ; \mathbb {R}^{d}), \end{aligned}$$

5.2 $$\begin{aligned}&\limsup _{\varepsilon \rightarrow 0} \root 4 \of {\varepsilon } \Vert y_{0, \varepsilon }\Vert _{ H^{4} (\Omega ) } <+\infty \,. \end{aligned}$$

This can be achieved by considering regularizations $(\varphi _\varepsilon )_\varepsilon \subset C^\infty _c(\Omega ;\mathbb {R}^d)$ with $\varphi _\varepsilon \rightarrow \Delta y_{0} \in L^p(\Omega ;\mathbb {R}^d)$, and choosing $y_{0, \varepsilon } \in C^\infty (\Omega ;\mathbb {R}^d) \cap \mathcal {Y}_\textbf{id}$ as the solution to $\Delta y_{0, \varepsilon } = \varphi _\varepsilon$. Then, $\partial _\nu \Delta y_{0,\varepsilon } = \Delta y_{0,\varepsilon } = 0$ on $\partial \Omega$ holds by construction and (5.1)–(5.2) can be achieved by the elliptic regularity estimate $\Vert y_{0, \varepsilon } - y_0 \Vert _{W^{2,p}(\Omega )} \le C \Vert \Delta ( y_{0, \varepsilon } - y_0) \Vert _{L^p(\Omega )}$, see [32, Lemma 9.17].

For every $\varepsilon >0$, in Theorem 2.2 we have shown the existence of a solution $(y_{\varepsilon }, \theta _{\varepsilon })$ to the regularized thermo-elastodynamic system (2.21)–(2.22). In the following lemma, we summarize the compactness properties of such sequences.

### Lemma 5.1

Let $(y_{\varepsilon }, \theta _{\varepsilon })$ be a sequence of solutions to (2.21)–(2.22) with initial data $(y_{0, \varepsilon }, y'_{0, \varepsilon }, \theta _{0})$ given by Theorem 2.2. Then, there exists $(y, \theta ) \in (L^\infty (I;\mathcal {Y}_{\textbf{id}}) \cap H^{1}( I; H^{1} (\Omega ;$ $\mathbb {R}^{d}))) \times L^{1} ( I; W^{1, 1}_{+}(\Omega ))$ such that, up to a subsequence, it holds for any $q \in (1, 2^{*} )$ that

5.3a $$\begin{aligned} y_{\varepsilon }&{\mathop {\rightharpoonup }\limits ^{*}}y \text { weakly* in } L^\infty (I; W^{2,p}(\Omega ;\mathbb {R}^d)) \text { and weakly in } H^{1}( I; H^{1}(\Omega ; \mathbb {R}^{d})) , \end{aligned}$$

5.3b $$\begin{aligned} y_{\varepsilon }&\rightarrow y \text { in } L^\infty (I; W^{1, \infty }(\Omega ; \mathbb {R}^{d })) \text { and in } L^2(I; W^{2, p}(\Omega ; \mathbb {R}^d)), \end{aligned}$$

5.3c $$\begin{aligned} \partial _{t} y_{\varepsilon }&\rightarrow \partial _{t} y \text { in }L^{2}(I; L^{q} (\Omega ; \mathbb {R}^{d})), \end{aligned}$$

5.3d $$\begin{aligned} \theta _{\varepsilon }&\rightharpoonup \theta \quad \text { and } \quad w_{\varepsilon } \rightharpoonup w \text { weakly in } L^r(I; W^{1,r}(\Omega )) \text { for any } r \in [1, \tfrac{d+2}{d+1}), \end{aligned}$$

5.3e $$\begin{aligned} \theta _{\varepsilon }&\rightarrow \theta \quad \text { and } \quad w_{\varepsilon } \rightarrow w \text { in } L^s(I \times \Omega ) \text { for any } s \in [1, \tfrac{d+2}{d}). \end{aligned}$$

where $$w :=W^{\textrm{in}}(\nabla y, \theta )$$.

### Proof

The convergences in (5.3a) and (5.3d)–(5.3e) as well as the first convergence in (5.3b) are obtained arguing as in the proof of Proposition 3.10, relying on the estimates (4.2a)–(4.2e). As for (5.3c), we apply the Aubin-Lions’ lemma as follows: by (4.2b) we have that $\partial _{t} y_{\varepsilon }$ is bounded in $L^{2} (I; H^{1}(\Omega ; \mathbb {R}^{d}))$ and Proposition 4.3 yields that $\partial ^{2}_{tt} y_{\varepsilon }$ is bounded in $L^{2} (I; ( H^{3} (\Omega ; \mathbb {R}^{d}) \cap H^{1}_{0} (\Omega ; \mathbb {R}^{d}))^{*})$. Hence, (5.3c) holds by the compact embedding $H^{1}(\Omega ; \mathbb {R}^{d}) \subset \subset L^q(\Omega ;\mathbb {R}^d)$ for $q < 2^*$. The identification $w = W^\textrm{in} (\nabla y, \theta )$ follows as in the proof of Proposition 3.10, cf. also [2, Lemma 4.2]. Note that by (4.3a) and elliptic regularity we follow that $y_\varepsilon$ is bounded in $L^2(I; H^3(\Omega ; \mathbb {R}^d))$. Consequently, yet another application of the Aubin-Lions’ lemma using also the boundedness of $\partial _t y_\varepsilon$ in $L^2(I \times \Omega ; \mathbb {R}^d)$ shows the second convergence in (5.3b). $\square$

### The mechanical equation

We recall that the mechanical equation (2.21) is equivalent to the formulation in (4.6).

#### Proposition 5.2

Let $(y, \theta )$ be as in Lemma 5.1. Then, $(y, \theta )$ satisfies (2.26).

#### Proof

We test (4.6) with $$z \in C^{\infty } (I \times \overline{\Omega }; \mathbb {R}^{d})$$ with $$z = 0 $$ in $$I \times \partial \Omega $$ and $$z(T) = 0$$. Thanks to the convergences in (5.3a)–(5.3e) and to assumptions ((W.1)), ((H.1)) and ((H.3)), ((C.1)), ((D.1)), and (5.1), we have that

5.4a $$\begin{aligned} \int _{I} \int _{\Omega } \partial _{F} W(\nabla y_{\varepsilon }, \theta _{\varepsilon }) : \nabla z \textrm{d}x \textrm{d}t&\rightarrow \int _{I} \int _{\Omega } \partial _{F} W(\nabla y, \theta ) : \nabla z \textrm{d}x \textrm{d}t\,,\end{aligned}$$

5.4b $$\begin{aligned} \int _{I} \int _{\Omega } DH(\Delta y_{\varepsilon }) \cdot \Delta z \textrm{d}x \textrm{d}t&\rightarrow \int _{I} \int _{\Omega } DH(\Delta y) \cdot \Delta z \textrm{d}x \textrm{d}t \,,\end{aligned}$$

5.4c $$\begin{aligned} \int _I \int _{\Omega } \partial _{\dot{F}} R(\nabla y_\varepsilon , \partial _t \nabla y_\varepsilon , \theta _\varepsilon ) : \nabla z \textrm{d}x \textrm{d}t&\rightarrow \int _I \int _{\Omega } \partial _{\dot{F}} R(\nabla y, \partial _t \nabla y, \theta ) : \nabla z \textrm{d}x \textrm{d}t \,,\end{aligned}$$

5.4d $$\begin{aligned} \int _{I} \int _{\Omega } \partial _{t} y_{\varepsilon } \cdot \partial _t z\textrm{d}x \textrm{d}t&\rightarrow \int _{I} \int _{\Omega } \partial _{t} y \cdot \partial _t z\textrm{d}x \textrm{d}t \,,\end{aligned}$$

5.4e $$\begin{aligned} \int _{\Omega } y_{0, \varepsilon } \cdot z(0) \textrm{d}x&\rightarrow \int _{\Omega } y_{0} \cdot z(0) \textrm{d}x \,. \end{aligned}$$

In particular, in (5.4b) we have used (5.3b) and in (5.4c) we have exploited the linear structure of $\partial _{\dot{F}} R$ with respect to $\partial _{t} y_{\varepsilon }$. Finally, estimate (4.2b) implies that

$$\begin{aligned} \varepsilon \int _{I} \int _{\Omega } \partial _t \nabla \Delta y_\varepsilon : \nabla \Delta z \textrm{d}x \textrm{d}t \rightarrow 0\,. \end{aligned}$$

Hence, the pair $(y, \theta )$ satisfies (2.26). $\square$

### The heat-transfer equation

We are left to consider the limit as $\varepsilon \rightarrow 0$ in the heat-transfer equation (2.22). To this purpose, we now derive a weaker form of the regularized heat equation which is suitable for the limit procedure. This relies on integration by parts and on the chain rule for the mechanical energy. For notational convenience, for $\psi \in C^\infty (\overline{\Omega })$ we define

5.5 $$\begin{aligned} \mathcal {E}(y,\theta ;\psi ) = \int _\Omega \Big (W^{\textrm{el}}(\nabla y) + H(\Delta y) + W^\textrm{in}(y,\theta ) \Big ) \psi \textrm{d}x. \end{aligned}$$

Notice that for the limiting passage we will also crucially use the bounds in (4.3).

#### Proposition 5.3

For every $\varepsilon >0$, every $\varphi \in C^{\infty } (I \times \overline{\Omega })$ of the form $\varphi = \psi \eta$ for $\psi \in C^{\infty } (\overline{\Omega })$ and $\eta \in C^{\infty }( I )$ with $\eta (T) = 0$ it holds

5.6 $$\begin{aligned} 0 =&\int _I\int _\Omega \eta \, \mathcal {K}(\nabla y_{\varepsilon }, \theta _{\varepsilon }) \nabla \theta _{\varepsilon } \cdot \nabla \psi - \kappa \int _I \int _{\partial \Omega } \eta \psi \, (\theta _\flat - \theta _{\varepsilon }) \textrm{d}\mathcal {H}^{d-1} \textrm{d}t - \int _I\int _\Omega \psi \eta \, f \cdot \partial _{t} y_{\varepsilon } \textrm{d}x \textrm{d}t \nonumber \\&- \int _I \partial _{t} \eta \, \Big ( \mathcal {E}(y_{\varepsilon }, \theta _\varepsilon ; \psi ) + \int _\Omega \frac{\rho }{2} | \partial _{t} y_{\varepsilon }|^{2} \psi \textrm{d}x \Big ) \textrm{d}t - \eta (0) \, \Big ( \mathcal {E}(y_{0,\varepsilon }, \theta _0; \psi ) + \int _{\Omega } \frac{\rho }{2} | y'_{0, \varepsilon }|^{2} \psi \, \textrm{d}x \Big ) \nonumber \\&+ \int _I\int _\Omega \eta \Big ( \partial _F W(\nabla y_{\varepsilon }, \theta _{\varepsilon }) + \partial _{\dot{F}} R (\nabla y_{\varepsilon }, \partial _{t} \nabla y_{\varepsilon }, \theta _{\varepsilon }) \Big ) : (\partial _{t} y_{\varepsilon } \otimes \nabla \psi ) \, \textrm{d}x \, \textrm{d}t \nonumber \\&- \int _I\int _\Omega \eta \, {D H}(\Delta y_{\varepsilon }) \cdot \partial _{t} y_{\varepsilon } \Delta \psi \, \textrm{d}x \textrm{d}t - 2\int _I\int _\Omega \eta \, \nabla ({D H}(\Delta y_{\varepsilon }) ) : (\partial _{t} y_{\varepsilon } \otimes \nabla \psi )\, \textrm{d}x \textrm{d}t \nonumber \\&- \varepsilon \int _I\int _\Omega \eta \, \partial _{t} \Delta ^{2}y_{\varepsilon } \cdot \big ( 2\partial _{t} \nabla y_{\varepsilon } \nabla \psi + \textrm{div} (\partial _{t} y_{\varepsilon } \otimes \nabla \psi ) \big ) \textrm{d}x \textrm{d}t \nonumber \\&+ \varepsilon \int _I\int _\Omega \eta \, \partial _{t} \nabla \Delta y_{\varepsilon } : \partial _{t} \nabla y_{\varepsilon } \Delta \psi \textrm{d}x \textrm{d}t - 2\varepsilon \int _I\int _\Omega \eta \, \partial _{t} \nabla \Delta y_{\varepsilon } : \partial _{t} \nabla y_{\varepsilon } \nabla ^{2}\psi \textrm{d}x \textrm{d}t . \end{aligned}$$

Note that except for the $\varepsilon$-dependent terms this formulation coincides with the one in (2.27).

#### Proof

For $\varepsilon >0$, we define $z :=\eta \psi \partial _{t} y_{\varepsilon }$ and, by (4.2b), we note that $z \in L^{2} (I; H^{3}(\Omega ; \mathbb {R}^{d}))$ and $z = 0$ in $I \times \partial \Omega$. Recall that $\partial ^{2}_{tt} y_{\varepsilon } \in L^{2}(I; (H^{3} (\Omega ; \mathbb {R}^{d}) \cap H^{1}_{0} (\Omega ; \mathbb {R}^{d}))^{*})$ by Proposition 4.3. Testing the heat-transfer equation (2.22) and performing an integration by parts in time we may write

5.7 $$\begin{aligned} \Pi&:=\int _I\int _\Omega \eta \psi \, \xi ( \nabla y_{\varepsilon } , \partial _t \nabla y_{\varepsilon } , \theta _{\varepsilon } ) \textrm{d}x \textrm{d}t + \varepsilon \int _I \int _{\Omega } \eta \psi \, | \partial _{t} \nabla \Delta y_{\varepsilon } |^{2} \textrm{d}x \textrm{d}t \nonumber \\&+ \int _I\int _\Omega \eta \psi \, \partial _F W^{\textrm{cpl}}(\nabla y_{\varepsilon }, \theta _{\varepsilon }) : \partial _t \nabla y_{\varepsilon } \, \textrm{d}x \textrm{d}t \nonumber \\&= \int _I\int _\Omega \Big ( \eta \, \mathcal {K}(\nabla y_{\varepsilon }, \theta _{\varepsilon }) \nabla \theta _{\varepsilon } \cdot \nabla \psi - \psi \, w_{\varepsilon } \partial _{t} \eta \Big ) \, \textrm{d}x \textrm{d}t \nonumber \\&- \kappa \int _I \int _{\partial \Omega } \eta \psi \, (\theta _\flat - \theta _{\varepsilon }) \textrm{d}\mathcal {H}^{d-1} \textrm{d}t - \int _\Omega w_{0,\varepsilon } \psi \eta ( 0 ) \textrm{d}x. \end{aligned}$$

Our goal is to rewrite the terms on the left hand side, i.e., $\Pi$. To this end, we test the regularized mechanical equation (2.21) with $z = \eta \psi \partial _{t} y_{\varepsilon }$: this yields

5.8 $$\begin{aligned}&\int _I\int _\Omega \eta \Big ( \partial _F W^{\textrm{el}}(\nabla y_\varepsilon ) + \partial _{F} W^\textrm{cpl} (\nabla y_{\varepsilon }, \theta _{\varepsilon }) \Big ) : \nabla (\psi \partial _{t} y_{\varepsilon }) \textrm{d}x \textrm{d}t \nonumber \\&+ \int _I\int _\Omega \eta \partial _{\dot{F}} R(\nabla y_\varepsilon , \partial _t \nabla y_\varepsilon , \theta _\varepsilon ) : \nabla (\psi \partial _{t} y_{\varepsilon }) \textrm{d}x \textrm{d}t \nonumber \\&\quad + \varepsilon \int _I \int _{\Omega } \eta \, \partial _{t} \nabla \Delta y_{\varepsilon } : \nabla \Delta (\psi \partial _{t} y_{\varepsilon }) \textrm{d}x \textrm{d}t + \int _I\int _\Omega \eta D H( \Delta y_\varepsilon ) \cdot \Delta (\psi \partial _{t} y_{\varepsilon } ) \, \textrm{d}x \textrm{d}t \nonumber \\  &\quad \quad \quad \quad \quad \quad \quad \quad \quad \quad \quad \quad \quad \ = \int _I\int _\Omega \psi \eta \, f \cdot \partial _{t} y_{\varepsilon } \textrm{d}x \textrm{d}t - \rho \int _I \left\langle \partial ^{2}_{tt} y_{\varepsilon } , \partial _{t} y_{\varepsilon } \, \eta \psi \right\rangle \textrm{d}t .\end{aligned}$$

We expand the terms on the left-hand side of (5.8) by expanding $\nabla (\psi \partial _{t} y_{\varepsilon })$, $\nabla \Delta (\psi \partial _{t} y_{\varepsilon })$, and $\Delta (\psi \partial _{t} y_{\varepsilon })$. This yields

5.9 $$\begin{aligned}&\int _I\int _\Omega \! \eta \Big ( \partial _F W^{\textrm{el}}(\nabla y_\varepsilon ) + \partial _{F} W^\textrm{cpl} (\nabla y_{\varepsilon }, \theta _{\varepsilon }) \Big ) : \nabla (\psi \partial _{t} y_{\varepsilon }) \textrm{d}x \textrm{d}t \nonumber \\&= \int _I\int _\Omega \! \eta \psi \, \partial _{F} W^\textrm{cpl} (\nabla y_{\varepsilon }, \theta _{\varepsilon }) : \partial _{t} \nabla y_{\varepsilon } \textrm{d}x \textrm{d}t + J_0 + J_1,\nonumber \\&\int _I\int _\Omega \eta \partial _{\dot{F}} R(\nabla y_\varepsilon , \partial _t \nabla y_\varepsilon , \theta _\varepsilon ) : \nabla (\psi \partial _{t} y_{\varepsilon }) \textrm{d}x \textrm{d}t \nonumber \\&= \int _I\int _\Omega \eta \psi \, \partial _{\dot{F}} R(\nabla y_\varepsilon , \partial _t \nabla y_\varepsilon , \theta _\varepsilon ) : \partial _{t} \nabla y_{\varepsilon } \textrm{d}x \textrm{d}t + J_2,\nonumber \\&\varepsilon \int _I\int _\Omega \eta \, \partial _{t} \nabla \Delta y_{\varepsilon } : \nabla \Delta (\psi \partial _{t} y_{\varepsilon }) \textrm{d}x \textrm{d}t \nonumber \\&= \varepsilon \int _I\int _\Omega \eta \psi \, |\partial _{t} \nabla \Delta y_{\varepsilon }|^{2} \textrm{d}x \textrm{d}t + J_3 + J_4, \nonumber \\ \int _I\int _\Omega \eta D&H( \Delta y_\varepsilon ) \cdot \Delta (\psi \partial _{t} y_{\varepsilon } ) \, \textrm{d}x \textrm{d}t \nonumber \\&= \int _I\int _\Omega \eta \psi \, {D H}(\Delta y_\varepsilon ) \cdot \partial _{t}\Delta y_{\varepsilon } \, \textrm{d}x \textrm{d}t + J_5, \end{aligned}$$

where for brevity we have written

5.10 $$\begin{aligned} J_0&:=\int _I\int _\Omega \eta \psi \, \partial _{F} W^\textrm{el} (\nabla y_{\varepsilon }) : \partial _{t} \nabla y_{\varepsilon } \textrm{d}x \textrm{d}t, \nonumber \\ J_1&:=\int _I\int _\Omega \eta \Big ( \partial _F W^{\textrm{el}}(\nabla y_\varepsilon ) + \partial _{F} W^\textrm{cpl} (\nabla y_{\varepsilon }, \theta _{\varepsilon }) \Big ) : (\partial _{t} y_{\varepsilon } \otimes \nabla \psi ) \, \textrm{d}x \, \textrm{d}t, \nonumber \\ J_2&:=\int _I\int _\Omega \eta \, \partial _{\dot{F}} R(\nabla y_\varepsilon , \partial _t \nabla y_\varepsilon , \theta _\varepsilon ) : (\partial _{t} y_{\varepsilon } \otimes \nabla \psi ) \textrm{d}x \textrm{d}t, \nonumber \\ J_3&:=\varepsilon \int _I\int _\Omega \eta \, \partial _{t} \nabla \Delta y_{\varepsilon } : ( \partial _{t} \Delta y_{\varepsilon } \otimes \nabla \psi ) \textrm{d}x \textrm{d}t , \nonumber \\ J_4&:=\varepsilon \int _I\int _\Omega \eta \, \partial _{t} \nabla \Delta y_{\varepsilon } : \nabla ( \partial _{t} \nabla y_{\varepsilon } \nabla \psi ) \textrm{d}x \textrm{d}t \nonumber \\&\quad + \varepsilon \int _I\int _\Omega \eta \, \partial _{t} \nabla \Delta y_{\varepsilon } : \nabla (\textrm{div} ( \partial _{t} y_{\varepsilon } \otimes \nabla \psi )) \textrm{d}x \textrm{d}t , \nonumber \\ J_5&:=\int _I\int _\Omega \eta \, {D H}(\Delta y_{\varepsilon }) \cdot \partial _{t} y_{\varepsilon } \Delta \psi \, \textrm{d}x \textrm{d}t + 2\int _I\int _\Omega \eta \, {D H}(\Delta y_{\varepsilon }) \cdot \partial _{t} \nabla y_{\varepsilon } \nabla \psi \, \textrm{d}x \textrm{d}t. \end{aligned}$$

Recalling (2.11), we see that the first three terms on the right-hand sides of (5.9) correspond to the terms in (5.7) whose sum is denoted by $\Pi$. This along with (5.8) yields

$$\begin{aligned}&\Pi + \sum _{i=0}^4J_i = \int _I\int _\Omega \psi \eta \, f \cdot \partial _{t} y_{\varepsilon } \textrm{d}x \textrm{d}t - \int _I\int _\Omega \eta D H( \Delta y_\varepsilon ) \cdot \Delta (\psi \partial _{t} y_{\varepsilon } ) \, \textrm{d}x \textrm{d}t \\&- \rho \int _I \left\langle \partial ^{2}_{tt} y_{\varepsilon } , \partial _{t} y_{\varepsilon } \, \eta \psi \right\rangle \textrm{d}t . \end{aligned}$$

Using the last equation in (5.9) and the definition of $J_0$, we get

5.11 $$\begin{aligned}&\Pi + \sum _{i=1}^5J_i = \int _I\int _\Omega \psi \eta \, \Big ( f \cdot \partial _{t} y_{\varepsilon } - \partial _{F} W^\textrm{el} (\nabla y_{\varepsilon }) : \partial _{t} \nabla y_{\varepsilon } - {D H}(\Delta y_\varepsilon ) \cdot \partial _{t}\Delta y_{\varepsilon } \Big ) \, \textrm{d}x \textrm{d}t \nonumber \\&\quad - \rho \int _I \left\langle \partial ^{2}_{tt} y_{\varepsilon } , \partial _{t} y_{\varepsilon } \, \eta \psi \right\rangle \textrm{d}t \,. \end{aligned}$$

Using the chain rule and $\eta (T) = 0$, we rewrite the last term on the right-hand side as

5.12 $$\begin{aligned} \rho \int _I \left\langle \partial ^{2}_{tt} y_{\varepsilon }, \partial _t y_{\varepsilon } \psi \eta \right\rangle \textrm{d}t&= \frac{\rho }{2} \int _I\int _\Omega \frac{\textrm{d}}{\textrm{d}t} (| \partial _{t} y_{\varepsilon }|^{2} \eta )\psi \textrm{d}x \textrm{d}t - \frac{\rho }{2} \int _I\int _\Omega \psi | \partial _{t} y_{\varepsilon }|^{2} \partial _{t} \eta \textrm{d}x \textrm{d}t \nonumber \\&= -\frac{\rho }{2} \int _{\Omega } \psi \eta (0) \, | y'_{0, \varepsilon }|^{2} \, \textrm{d}x - \frac{\rho }{2} \int _I\int _\Omega \psi | \partial _{t} y_{\varepsilon }|^{2} \partial _{t} \eta \textrm{d}x \textrm{d}t . \end{aligned}$$

By the chain rule for the mechanical energy, see (3.74) (for $y_\varepsilon$ in place of $y_h$), and integration by parts we have

5.13 $$\begin{aligned} \int _I\int _\Omega \eta \psi \,&\Big ( \partial _{F} W^\textrm{el} (\nabla y_{\varepsilon }) : \partial _{t} \nabla y_{\varepsilon } + {D H}(\Delta y_\varepsilon ) \cdot \partial _{t}\Delta y_{\varepsilon } \Big ) \, \textrm{d}x \textrm{d}t \nonumber \\&= \int _I \eta \, \frac{\textrm{d}}{\textrm{d}t} \int _{\Omega } \psi \big ( W^{\textrm{el}}(\nabla y_{\varepsilon }) + H(\Delta y_{\varepsilon } ) \big )\textrm{d}x \textrm{d}t \nonumber \\&= - \int _{\Omega } \eta (0) \, \psi \big ( W^{\textrm{el}}(\nabla y_{0,\varepsilon }) + H(\Delta y_{0,\varepsilon } ) \big )\textrm{d}x \nonumber \\&- \int _I\int _\Omega \partial _{t} \eta \, \psi \big ( W^{\textrm{el}}(\nabla y_{\varepsilon }) + H(\Delta y_{\varepsilon } ) \big )\textrm{d}x \textrm{d}t . \end{aligned}$$

Next, we manipulate $J_3$, $J_4$, $J_5$. Recall that $\Delta y_{\varepsilon } = 0$ and $\partial _{t} y_{\varepsilon } =0$ on $\partial \Omega$ for a.e. $t \in I$ (see (4.4)) and $DH(0)=0$ by (2.4)–(2.5). We perform an integration by parts in $J_5$ to get

5.14 $$\begin{aligned} J_5&= \int _I\int _\Omega \eta \, {D H}(\Delta y_{\varepsilon }) \cdot \partial _{t} y_{\varepsilon } \Delta \psi \, \textrm{d}x \textrm{d}t - 2\int _I\int _\Omega \eta \, \nabla ({D H}(\Delta y_{\varepsilon }) ) : (\partial _{t} y_{\varepsilon } \otimes \nabla \psi )\, \textrm{d}x \textrm{d}t \nonumber \\&\quad - 2 \int _I\int _\Omega \eta \, {D H}(\Delta y_{\varepsilon }) \cdot \partial _{t} y_{\varepsilon } \Delta \psi \, \textrm{d}x \textrm{d}t \nonumber \\&= - \int _I\int _\Omega \eta \, {D H}(\Delta y_{\varepsilon }) \cdot \partial _{t} y_{\varepsilon } \Delta \psi \, \textrm{d}x \textrm{d}t - 2\int _I\int _\Omega \eta \, \nabla ({D H}(\Delta y_{\varepsilon }) ) : (\partial _{t} y_{\varepsilon } \otimes \nabla \psi )\, \textrm{d}x \textrm{d}t . \end{aligned}$$

By integrating by parts and recalling the boundary conditions $\Delta \partial _{t} y_{\varepsilon } = \partial _{\nu } \Delta \partial _{t} y_{\varepsilon } = 0$ on $\partial \Omega$ for a.e. $t \in I$ (see (4.4)), we rewrite $J_4$ as

5.15 $$\begin{aligned} J_4 = - \varepsilon \int _I\int _\Omega \eta \, \partial _{t} \Delta ^{2}y_{\varepsilon } \cdot \big ( \partial _{t} \nabla y_{\varepsilon } \nabla \psi + \textrm{div} (\partial _{t} y_{\varepsilon } \otimes \nabla \psi ) \big ) \textrm{d}x \textrm{d}t, \end{aligned}$$

and, by elementary but tedious computations, $J_3$ can be written as

5.16 $$\begin{aligned} \frac{1}{\varepsilon } J_3&= \int _I\int _\Omega \eta \, \partial _{t} \nabla \Delta y_{\varepsilon } : ( \partial _{t} \Delta y_{\varepsilon } \otimes \nabla \psi ) \textrm{d}x \textrm{d}t \nonumber \\&= - \int _I\int _\Omega \eta \, \partial _{t} \Delta y_{\varepsilon } \cdot \big ( (\partial _{t} \nabla \Delta y_{\varepsilon } \nabla \psi ) + \partial _{t} \Delta y_{\varepsilon } \Delta \psi \big ) \textrm{d}x \textrm{d}t \nonumber \\&= \int _I\int _\Omega \eta \, \partial _{t}\nabla \Delta y_{\varepsilon } : \big ( \partial _{t} \nabla ^{2} y_{\varepsilon } \nabla \psi + \partial _{t} \nabla y_{\varepsilon } \Delta \psi \big ) \textrm{d}x \textrm{d}t \nonumber \\&+ \int _I\int _\Omega \eta \, \partial _{t} \Delta y_{\varepsilon } \cdot \big (\partial _{t} \nabla ^{2} y_{\varepsilon } \nabla ^{2} \psi + \partial _{t} \nabla y_{\varepsilon } \nabla \Delta \psi \big ) \textrm{d}x \textrm{d}t \nonumber \\&= - \int _I\int _\Omega \eta \, \partial _{t} \Delta ^{2}y_{\varepsilon } \cdot ( \partial _{t} \nabla y_{\varepsilon } \nabla \psi ) \textrm{d}x \textrm{d}t + \int _I\int _\Omega \eta \, \partial _{t} \nabla \Delta y_{\varepsilon } : \partial _{t} \nabla y_{\varepsilon } \Delta \psi \textrm{d}x \textrm{d}t \nonumber \\&\quad - 2\int _I\int _\Omega \eta \, \partial _{t} \nabla \Delta y_{\varepsilon } : \partial _{t} \nabla y_{\varepsilon } \nabla ^{2}\psi \textrm{d}x \textrm{d}t \,. \end{aligned}$$

Recalling the definition in (5.5) and combining (5.7) with (5.10)–(5.16) we obtain the statement. More precisely, (5.12) and (5.13) contribute to the second line in (5.6), the third line corresponds to $J_1+J_2$ (see (5.10)) and the last three lines correspond to $J_3+J_4+J_5$ (see (5.14)–(5.16)). $\square$

We are now in a position to pass to the limit as $\varepsilon \rightarrow 0$ in the modified heat-transfer equation (5.6). This will conclude the proof of Theorem 2.5.

#### Proposition 5.4

Let $(y, \theta )$ be given by Lemma 5.1. Then, $(y, \theta )$ satisfies (2.27).

#### Proof

In order to show (2.27), by a density argument it suffices to consider test functions of the form $\varphi = \psi \eta$ for $\psi \in C^{\infty } (\overline{\Omega })$ and $\eta \in C^{\infty } (I)$ with $\eta (T) = 0$. We test (5.6) with $\varphi$ and pass to the limit term by term.

Thanks to the convergences stated in Lemma 5.1, the estimates (4.2)–(4.3), and the assumptions (W.1)–(W.3), (H.1)–(H.3), (C.1)–(C.5), (D.1)–(D.2), and (5.1), we have that each of the terms in the first three lines of (5.6) converges to the respective term in the first three lines of (2.27) (with $\eta \psi$ in place of $\varphi$). Here, we againg exploit that $\partial _{\dot{F}} R$ is linear in the second entry, cf. (D.1). Therefore, it suffices to show

5.17 $$\begin{aligned}&\lim _{\varepsilon \rightarrow 0} \Big ( \int _I\int _\Omega \eta \, {D H}(\Delta y_{\varepsilon }) \cdot \partial _{t} y_{\varepsilon } \Delta \psi \, \textrm{d}x \textrm{d}t + 2\int _I\int _\Omega \eta \, \nabla ({D H}(\Delta y_{\varepsilon }) ) : (\partial _{t} y_{\varepsilon } \otimes \nabla \psi )\, \textrm{d}x \textrm{d}t \Big ) \nonumber \\&= \int _I\int _\Omega \eta \, {D H}(\Delta y) \cdot \partial _{t} y \Delta \psi \, \textrm{d}x \textrm{d}t + 2\int _I\int _\Omega \eta \, \nabla ({D H}(\Delta y) ) : (\partial _{t} y \otimes \nabla \psi )\, \textrm{d}x \textrm{d}t , \end{aligned}$$

5.18 $$\begin{aligned} \lim _{\varepsilon \rightarrow 0} \varepsilon \int _I\int _\Omega \eta \, \partial _{t} \Delta ^{2}y_{\varepsilon } \cdot \big ( 2\partial _{t} \nabla y_{\varepsilon } \nabla \psi + \textrm{div} (\partial _{t} y_{\varepsilon } \otimes \nabla \psi ) \big ) \textrm{d}x \textrm{d}t = 0, \end{aligned}$$

5.19 $$\begin{aligned} \lim _{\varepsilon \rightarrow 0} \Big ( \varepsilon \int _I\int _\Omega \eta \, \partial _{t} \nabla \Delta y_{\varepsilon } : \partial _{t} \nabla y_{\varepsilon } \Delta \psi \textrm{d}x \textrm{d}t - 2\varepsilon \int _I\int _\Omega \eta \, \partial _{t} \nabla \Delta y_{\varepsilon } : \partial _{t} \nabla y_{\varepsilon } \nabla ^{2}\psi \textrm{d}x \textrm{d}t \Big ) = 0 . \end{aligned}$$

We start with (5.17). Recalling that $\partial _t y_\varepsilon$ converges strongly in $L^2(I;L^p(\Omega ;\mathbb {R}^d))$ by (5.3c) (recall $p <6$ for $d=3$), the key point is to show the strong (resp. weak) convergence of ${D H}(\Delta y_{\varepsilon })$ (resp. $\nabla ({D H}(\Delta y_{\varepsilon }))$) in $L^2(I; L^{p'} (\Omega ))$. Since $y_{\varepsilon } \rightarrow y$ in $L^{2} (I; W^{2, p} (\Omega ; \mathbb {R}^{d}))$ (cf. (5.3b)), we infer by (H.3) that

5.20 $$\begin{aligned} \eta \,{D H}(\Delta y_{\varepsilon }) \ \rightarrow \ \eta {D H}(\Delta y) \qquad \text {in }L^{2} (I; L^{p'} (\Omega ; \mathbb {R}^{d})). \end{aligned}$$

In view of (4.3b), by weak compactness we also have that

5.21 $$\begin{aligned} \eta \,\nabla ({D H}(\Delta y_{\varepsilon })) \ \rightharpoonup \ \eta \nabla ( {D H}(\Delta y) )\qquad \text {in }L^{2}(I; L^{p'} (\Omega ; \mathbb {R}^{d \times d})). \end{aligned}$$

Now, (5.20)–(5.21) along with (5.3c) show (5.17).

Eventually, by Hölder’s inequality, we get that

$$\begin{aligned} \bigg |\int _I\int _\Omega \eta \, \partial _{t} \Delta ^{2}y_{\varepsilon }&\cdot \big ( 2\partial _{t} \nabla y_{\varepsilon } \nabla \psi + \textrm{div} ( \partial _{t} y_{\varepsilon } \otimes \nabla \psi ) \big ) \textrm{d}x \textrm{d}t \bigg | \\&\le C \Vert \partial _{t} \Delta ^{2}y_{\varepsilon } \Vert _{L^{2}(I \times \Omega )} \big ( \Vert \eta \nabla \psi \Vert _{L^{\infty } (\Omega )} + \Vert \eta \nabla ^2 \psi \Vert _{L^{\infty } (\Omega )} \big ) \\&\quad \big ( \Vert \partial _{t} y_{\varepsilon }\Vert _{L^{2}(I \times \Omega )} + \Vert \, \partial _{t} \nabla y_{\varepsilon }\Vert _{L^{2}(I \times \Omega )} \big ) \,, \\ \bigg |\int _I\int _\Omega \eta \, \partial _{t} \nabla \Delta y_{\varepsilon }&: \big ( 2 \partial _{t} \nabla y_{\varepsilon } \nabla ^{2} \psi - \partial _{t} \nabla y_{\varepsilon } \Delta \psi ) \textrm{d}x \textrm{d}t \bigg | \\&\le C \Vert \partial _{t} \nabla \Delta y_{\varepsilon } \Vert _{L^{2}(I \times \Omega )} \Vert \eta \nabla ^{2} \psi \Vert _{L^{\infty } (\Omega )} \Vert \partial _{t} \nabla y_{\varepsilon }\Vert _{L^{2}(I \times \Omega )}\,. \end{aligned}$$

Thus, we infer from (4.2b) and (4.3c) (recall $\varrho <1$) that (5.18)–(5.19) hold. This concludes the proof. $\square$

#### Proof of Theorem 2.5

The weak formulation follows from Propositions 5.2 and 5.4. We deduce the regularity $y\in L^2(I; H^3(\Omega ;\mathbb {R}^d))$ and $(1+|\Delta y|)^\frac{p-2}{2}|\nabla \Delta y|^2\in L^2(I\times \Omega )$ by the bound (4.3a) and lower semicontinuity of norms as $\varepsilon \rightarrow 0$, again applying an elliptic regularity estimate. $\square$

## Data Availability

The manuscript contains no associated data.

## A special case of elliptic regularity

We formulate and prove the lemma used in Subsection 3.3.

### Lemma A.1

(A special case of elliptic regularity) Consider the Banach space $X :=W^{2,q}(\Omega ; \mathbb {R}^d) \cap H^1_0(\Omega ; \mathbb {R}^d)$ for some $q>1$. Moreover, let $u \in H^3(\Omega ; \mathbb {R}^d) \cap H^1_0(\Omega ; \mathbb {R}^d)$ and $g \in X^*$ be such that for all $z \in C^\infty (\overline{\Omega }; \mathbb {R}^d)$ with $z = 0$ on $\partial \Omega$ it holds that

A.1 $$\begin{aligned} \int _\Omega \nabla \Delta u : \nabla \Delta z\, dx = \langle g, z \rangle , \end{aligned}$$

where $\langle \cdot , \cdot \rangle$ denotes the duality pairing between *X* and $X^*$. Then, the following holds true:

- We have that $u \in W^{4,q'} (\Omega ; \mathbb {R}^d)$ with
  A.2 $$\begin{aligned} \Vert u \Vert _{ W^{4,q'}(\Omega )} \le C \Vert g\Vert _{X^*} + C|\mu | \end{aligned}$$
  for a constant $$C > 0 $$ only depending on $$\Omega $$, where . Moreover, the following boundary condition holds true:
  A.3 $$\begin{aligned} \partial _\nu \Delta u = 0 \qquad \mathcal {H}^{d-1}\text {-a.e. on } \partial \Omega . \end{aligned}$$
- If we additionally have $g \in H^{-1}(\Omega ; \mathbb {R}^d)$, then $u \in H^5(\Omega ; \mathbb {R}^d)$ with
  A.4 $$\begin{aligned} \Vert u \Vert _{H^5(\Omega )} \le C \Vert g\Vert _{H^{-1}(\Omega )} + C|\mu |, \end{aligned}$$
  satisfying the boundary condition
  A.5 $$\begin{aligned} \Delta ^{2}u = 0 \qquad \mathcal {H}^{d-1}-\text {a.e. on } \partial \Omega . \end{aligned}$$

### Proof

*Step 1* ($W^{4,q'}$-*regularity):* Using (A.1) and integrating by parts we see that for all $z \in C^\infty (\overline{\Omega }; \mathbb {R}^{d})$ with $z=\partial _\nu \Delta z = 0$ on $\partial \Omega$ it holds that

A.6 $$\begin{aligned}  &   \langle g, z \rangle = \int _\Omega \nabla \Delta u : \nabla \Delta z \textrm{d}x = - \int _\Omega \Delta u \cdot \Delta ^{2}z \textrm{d}x + \int _{\partial \Omega } \Delta u \cdot \partial _\nu \Delta z \textrm{d}\mathcal {H}^{d-1} \nonumber \\  &   \quad = - \int _\Omega \Delta u \cdot \Delta ^{2}z \textrm{d}x. \end{aligned}$$

By representation of the dual space we find $G,G_j,G_{ij} \in L^{q'}(\Omega ;\mathbb {R}^d)$ such that

A.7 $$\begin{aligned} \langle g ,z \rangle = \int _\Omega G\cdot z \textrm{d}x + \sum _{j=1}^d G_j\cdot \partial _jz \textrm{d}x + \sum _{i,j=1}^d G_{ij}\cdot \partial ^2_{ij}z \textrm{d}x \end{aligned}$$

with $\Vert G \Vert _{L^{q'}(\Omega )} + \sum _{j=1}^d\Vert G_j \Vert _{L^{q'}(\Omega )}+ \sum _{i,j=1}^d\Vert G_{ij} \Vert _{L^{q'}(\Omega )} \le C \Vert g \Vert _{X^*}$. We approximate *G*, $G_j$, and $G_{ij}$ by sequences $G^k, G_j^k, G_{ij}^k \in C^\infty _c(\Omega ;\mathbb {R}^d)$ converging to the respective functions in $L^{q'}(\Omega ;\mathbb {R}^d)$. We set

$$\begin{aligned} g_k :=G_k - \sum _{j=1}^d \partial _j G_j^k + \sum _{i,j=1}^d \partial _{ij}^2 G_{ij}^k, \end{aligned}$$

and let $v_k \in H^1_0(\Omega ; \mathbb {R}^d)$ be the weak solution to

$$\begin{aligned} \left\{ \begin{aligned} - \Delta v_k&= g_k  &   \text {in } \Omega , \\ v_k&= 0  &   \text {on } \partial \Omega . \end{aligned} \right. \end{aligned}$$

In particular, by integration by parts, for all $z \in C^\infty (\overline{\Omega }; \mathbb {R}^d)$ with $z = 0$ on $\partial \Omega$ it holds that

A.8 $$\begin{aligned} \int _\Omega v_k \cdot \Delta z \textrm{d}x = - \int _\Omega g_k \cdot z \, \textrm{d}x = - \int _\Omega \Big ( G_k \cdot z + \sum _{j=1}^d G_j^k \cdot \partial _j z + \sum _{i,j=1}^d G_{ij}^k \cdot \partial ^2_{ij} z \Big ) \, \textrm{d}x. \end{aligned}$$

Moreover, let $w_k \in H^1_0(\Omega ; \mathbb {R}^d)$ be the weak solution to

$$\begin{aligned} \left\{ \begin{aligned} \Delta w_k&= |v_k|^{\frac{2-q}{q-1}} v_k  &   \text {in } \Omega , \\ w_k&= 0  &   \text {on } \partial \Omega . \end{aligned} \right. \end{aligned}$$

As $\Omega$ has $C^5$-boundary, by elliptic regularity [36, Chapter 2] we see that $w_k \in W^{2,q}(\Omega ; \mathbb {R}^d)$ with

$$\begin{aligned} \Vert w_k\Vert _{W^{2,q}(\Omega )} \le C \Vert |v_k|^{\frac{2-q}{q-1}} v_k\Vert _{L^{q}(\Omega )} = C \Vert v_k\Vert ^{1/(q-1)}_{L^{q'}(\Omega )}, \end{aligned}$$

where the constant *C* only depends on $\Omega$. With (A.8), this shows

A.9 $$\begin{aligned} \Vert v_k\Vert _{L^{q'}(\Omega )}^{q'}&= \int _\Omega v_k \cdot \Delta w_k \textrm{d}x =- \int _\Omega g_k \cdot w_k \textrm{d}x \nonumber \\&\le C \Big ( \Vert G_k \Vert _{L^{q'}(\Omega )} + \sum _{j=1}^d \Vert G_j^k \Vert _{L^{q'}(\Omega )} + \sum _{i,j=1}^d \Vert G_{ij}^k \Vert _{L^{q'}(\Omega )} \Big ) \Vert w_k \Vert _{W^{2,q}(\Omega )} \nonumber \\  &\le C \Vert g \Vert _{X^*}\Vert v_k \Vert ^{1/(q-1)}_{L^{q'}(\Omega )}. \end{aligned}$$

Consequently, there exists $v \in L^{q'} (\Omega ; \mathbb {R}^d)$ such that, up to selecting a subsequence, $v_k \rightharpoonup v$ weakly in $L^{q'} (\Omega ; \mathbb {R}^d)$. Passing to the limit $k \rightarrow \infty$ in (A.8) and (A.9), and recalling (A.7), we discover that *v* satisfies

A.10 $$\begin{aligned} \int _\Omega v \cdot \Delta z \textrm{d}x&= -\langle g, z \rangle , \end{aligned}$$

A.11 $$\begin{aligned} \Vert v\Vert _{L^{q'}(\Omega )}&\le C \Vert g\Vert _{X^*}, \end{aligned}$$

for all $z \in C^\infty (\overline{\Omega }; \mathbb {R}^d)$ with $z = 0$ on $\partial \Omega$. Now, let $w \in H^1(\Omega ; \mathbb {R}^d)$ with  be the weak solution to

A.12 $$\begin{aligned} \left\{ \begin{aligned} \Delta w&= v - m  &   \text {in } \Omega , \\ \partial _\nu w&= 0  &   \text {on } \partial \Omega , \end{aligned} \right. \end{aligned}$$

with , i.e., $\int _\Omega w \textrm{d}x = 0$ and $- \int _\Omega \nabla w : \nabla z \textrm{d}x = \int _\Omega (v-m) \cdot z \textrm{d}x$ for all $z \in H^1(\Omega ; \mathbb {R}^d)$. As $\Omega$ has $C^5$-boundary and $v \in L^{q'} (\Omega ; \mathbb {R}^d)$, by elliptic regularity [36, Chapter 2] and (A.11) we derive that $w \in W^{2,q'}(\Omega ; \mathbb {R}^d)$ (i.e., (A.12) holds in a pointwise sense) and

A.13 $$\begin{aligned} \Vert w\Vert _{W^{2,q'}(\Omega )} \le C \Vert v - m \Vert _{L^{q'}(\Omega )} \le C \Vert g\Vert _{X^*}. \end{aligned}$$

Consequently, for *z* with $z = \partial _\nu \Delta z = 0$ on $\partial \Omega$ we derive, due to $\partial _\nu w = 0$ for $\mathcal {H}^{d-1}$-a.e. point in $\partial \Omega$, (A.10), and (A.12) that

$$\begin{aligned} \int _\Omega w \cdot \Delta ^{2}z \textrm{d}x&= -\int _\Omega \nabla w : \nabla \Delta z \textrm{d}x + \int _{\partial \Omega } w \cdot \partial _\nu \Delta z \textrm{d}\mathcal {H}^{d-1} \\  &= \int _\Omega \Delta w \cdot \Delta z \textrm{d}x - \int _{\partial \Omega } \partial _\nu w \cdot \Delta z \textrm{d}\mathcal {H}^{d-1} \\&= \int _\Omega v \cdot \Delta z \textrm{d}x - \int _\Omega m \cdot \Delta z \textrm{d}x = - \langle g, z \rangle - \int _{\Omega } m \cdot \Delta z \textrm{d}x\,. \end{aligned}$$

Using (A.6) we get

A.14 $$\begin{aligned} \int _\Omega (\Delta u - w) \cdot \Delta ^{2}z \textrm{d}x = 0 \end{aligned}$$

for all $z \in C^\infty (\Omega ; \mathbb {R}^d)$ with $z = \partial _\nu \Delta z = 0$ at $\mathcal {H}^{d-1}$-a.e. point in $\partial \Omega$ and . We now show that $\Delta u - w$ constant a.e. in $\Omega$. To this end, let $\varphi \in C^\infty (\Omega ; \mathbb {R}^d)$ with  be arbitrary. As $\Omega$ has $C^5$-boundary, by elliptic regularity [36, Chapter 2, Section 5] we can find $\tilde{z} \in H^2(\Omega ; \mathbb {R}^d)$ such that  and

$$\begin{aligned} \left\{ \begin{aligned} - \Delta \tilde{z}&= \varphi  &   \text {in } \Omega , \\ \partial _\nu \tilde{z}&= 0  &   \text {on } \partial \Omega , \end{aligned} \right. \end{aligned}$$

and, subsequently, we can find $z \in H^4(\Omega ; \mathbb {R}^d)$ satisfying

$$\begin{aligned} \left\{ \begin{aligned} - \Delta z&= \tilde{z}  &   \text {in } \Omega , \\ z&= 0  &   \text {on } \partial \Omega . \end{aligned} \right. \end{aligned}$$

Consequently, with (A.14) this leads to $\int _\Omega (\Delta u - w) \cdot \varphi \textrm{d}x = 0$, and by the arbitrariness of $\varphi$ to $\Delta u - w$ constant a.e. in $\Omega$. As , we get . We let $\nu \in H^1_0(\Omega ;\mathbb {R}^d)$ such that $\Delta \nu = \mu$. As by assumption $u \in H^1_0(\Omega ; \mathbb {R}^d)$ and $\Omega$ has $C^5$-boundary, and since $w \in W^{2,q'}(\Omega ; \mathbb {R}^d)$, by the above mentioned elliptic regularity we see that $u \in W^{4,q'} (\Omega ; \mathbb {R}^d)$ and

$$\begin{aligned} \Vert u -\nu \Vert _{W^{4,q'}(\Omega )} \le C \Vert \Delta u - \mu \Vert _{W^{2,q'}(\Omega )} = C \Vert w\Vert _{W^{2,q'}(\Omega )}. \end{aligned}$$

This along with (A.13) and the elliptic regularity estimate $\Vert \nu \Vert _{W^{4,q'}(\Omega )} \le C \Vert \Delta \nu \Vert _{W^{2,q'}(\Omega )} \le C|\mu |$ shows (A.2). Finally, (A.3) directly follows from $\Delta u - w$ constant and (A.12). This concludes the proof of (a).

*Step 2* ($H^5$-*regularity):* From now on, we assume that $g \in H^{-1}(\Omega ; \mathbb {R}^d)$. Since then also $g \in H^*$, Step 1 yields $u \in W^{4,q'} (\Omega ; \mathbb {R}^d)$. Thus, we can integrate by parts in (A.1) and use (A.3) to derive

A.15 $$\begin{aligned}  &   -\langle g, z \rangle = -\int _\Omega \nabla \Delta u : \nabla \Delta z \textrm{d}x = \int _\Omega \Delta ^{2}u : \Delta z \textrm{d}x - \int _{\partial \Omega } \partial _\nu \Delta u \cdot \Delta z \textrm{d}\mathcal {H}^{d-1} \nonumber \\  &   \quad = \int _\Omega \Delta ^{2}u : \Delta z \textrm{d}x \end{aligned}$$

for all $z \in C^\infty (\overline{\Omega }; \mathbb {R}^d)$ with $z = 0$ on $\partial \Omega$, where $\langle \cdot , \cdot \rangle$ now denotes the duality pairing between $H^1_0(\Omega ; \mathbb {R}^d)$ and $H^{-1}(\Omega ; \mathbb {R}^d)$. Let $v \in H^1_0(\Omega ; \mathbb {R}^d)$ be the weak solution to

A.16 $$\begin{aligned} \left\{ \begin{aligned} - \Delta v&= g  &   \text {in } \Omega , \\ v&= 0  &   \text {on } \partial \Omega . \end{aligned} \right. \end{aligned}$$

In particular, we have with Poincaré’s inequality that

A.17 $$\begin{aligned} \Vert v\Vert _{H^1(\Omega )}^2 \le C \Vert \nabla v\Vert _{L^2(\Omega )}^2 = \langle g, v \rangle \le \Vert g\Vert _{H^{-1}(\Omega )} \Vert v\Vert _{H^1(\Omega )}. \end{aligned}$$

Furthermore, for all $z \in C^\infty (\overline{\Omega }; \mathbb {R}^d)$ with $z = 0$ on $\partial \Omega$ it holds that

A.18 $$\begin{aligned} -\langle g, z \rangle = -\int _\Omega \nabla v : \nabla z \textrm{d}x = \int _\Omega v \cdot \Delta z - \int _{\partial \Omega } v \cdot \partial _{\nu } z \textrm{d}\mathcal {H}^{d-1} = \int _\Omega v\cdot \Delta z. \end{aligned}$$

Subtracting this from (A.15), we arrive at

$$\begin{aligned} \int _\Omega (\Delta ^{2}u - v) \cdot \Delta z \textrm{d}x = 0 \end{aligned}$$

for all $z \in C^\infty (\overline{\Omega }; \mathbb {R}^d)$ with $z = 0$ on $\partial \Omega$. By an argument similar to the one from Step 1 this leads to $\Delta ^{2}u = v$ a.e. in $\Omega$. This also shows $\int _\Omega v \textrm{d}x = \int _{\partial \Omega } \partial _\nu \Delta u \textrm{d}\mathcal {H}^{d-1} = 0$ by (A.3). Then, in view of (A.3) and (A.17), we derive by elliptic regularity for Neumann problems (see again [36, Chapter 2, Section 5]) that $\Delta u \in H^3(\Omega ; \mathbb {R}^d)$ such that

$$\begin{aligned} \Vert \Delta u -\mu \Vert _{H^3(\Omega )} \le C \Vert v\Vert _{H^1(\Omega )}\le C \Vert g\Vert _{H^{-1}(\Omega )}, \end{aligned}$$

where as before . Hence, as $u = 0$ for $\mathcal {H}^{d-1}$-a.e. point on $\partial \Omega$ and $\Omega$ has $C^5$-boundary, yet another application of elliptic regularity leads to $u \in H^5(\Omega ; \mathbb {R}^d)$ and the bound (A.4). Then, (A.5) follows from $\Delta ^{2}u = v$ a.e. in $\Omega$ and (A.16). $\square$
